# Physical properties and cytotoxicity of Cu(ii) and Zn(ii) complexes with a TMS-substituted indolo[2,3-*c*]quinoline-derived Schiff base†

**DOI:** 10.1039/d5dt00314h

**Published:** 2025-04-02

**Authors:** Christopher Wittmann, Iuliana Besleaga, Soheil Mahmoudi, Oleg Palamarciuc, Mihaela Balan-Porcarasu, Mihaela Dascalu, Sergiu Shova, Maria Cazacu, Mónika Kiricsi, Nóra Igaz, Orsolya Dömötör, Eva A. Enyedy, Dana Dvoranová, Peter Rapta, Vladimir B. Arion

**Affiliations:** a University of Vienna, Institute of Inorganic Chemistry Währinger Strasse 42 1090 Vienna Austria vladimir.arion@univie.ac.at; b University of Vienna, Vienna Doctoral School in Chemistry (DoSChem) Währinger Strasse 42 1090 Vienna Austria; c Inorganic Polymers Department, “Petru Poni” Institute of Macromolecular Chemistry Aleea Gr. Ghica Voda 41 A Iasi 700487 Romania; d Physics of Semiconductors and Devices Laboratory, Faculty of Physics and Engineering and Institute of Applied Physics, Moldova State University MD-2009 Chişinău Republic of Moldova; e NMR Laboratory, “Petru Poni” Institute of Macromolecular Chemistry Aleea Gr. Ghica Voda 41 A Iasi 700487 Romania; f Department of Biochemistry and Molecular Biology, University of Szeged Közép fasor 52 H-6726 Szeged Hungary; g Department of Molecular and Analytical Chemistry, Interdisciplinary Excellence Centre, University of Szeged Dóm tér 7-8, H-6720 Szeged Hungary enyedy@chem.u-szeged.hu; h Institute of Physical Chemistry and Chemical Physics, Faculty of Chemical and Food Technology, Slovak University of Technology in Bratislava SK-81237 Bratislava Slovakia peter.rapta@stuba.sk

## Abstract

The incorporation of non-native chemical elements, such as silicon, into drug molecules has gained significant attention as a strategy to broaden the chemical space in medicinal chemistry and develop novel drug candidates. Traditionally, research has focused on the isosteric replacement of a carbon atom with silicon (“silicon switch”) in known drug structures or the attachment of a trimethylsilyl (TMS) group to biologically active scaffolds. In this study, a TMS-substituted indoloquinoline-based Schiff base (HL^TMS^) and its corresponding metal complexes, **Cu(HL**^**TMS**^**)Cl**_**2**_ (1) and **Zn(HL**^**TMS**^**)Cl**_**2**_ (2), were synthesized and comprehensively characterized using elemental analysis, spectroscopic techniques (IR, UV-vis, ^1^H and ^13^C NMR for HL^TMS^ and 2), ESI mass spectrometry and single-crystal X-ray diffraction (SC-XRD) for 1 and electron diffraction (ED) for 2. The attachment of the TMS group enhanced the lipophilicity of HL^TMS^, while complex formation with Cu(ii) substantially improved the antiproliferative activity. Exploitation of their intrinsic fluorescence to investigate cellular uptake and intracellular localization in cancer cells was impeded by limited solubility. Both HL^TMS^ and 2 were found to generate reactive oxygen species under cell-free conditions in accord with their redox activity established by cyclic voltammetry. The photochemical activity of the indolo[2,3-*c*]quinoline-based proligand HL^TMS^ and its complexes 1 and 2 has been disclosed. The compounds exhibited significant toxicity on various human cancer cells and disrupted the mitochondrial membrane potential, suggesting the contribution of mitochondrial dysfunction, triggered by HL^TMS^ and its metal complexes, to their toxic effects. These findings highlight the potential of TMS-substituted Schiff bases as promising anticancer drug candidates.

## Abstract 2

The HL^TMS^, **Cu(HL**^**TMS**^**)Cl**_**2**_ (1) and **Zn(HL**^**TMS**^**)Cl**_**2**_ (2) induce mitochondrial disturbance contributing to their cytotoxicity.

## Graphical abstract

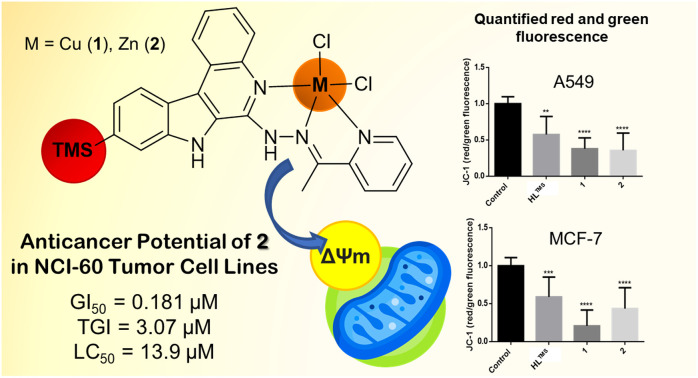

## Introduction

As carbon-based life forms, humans and living organisms in general are treated with medicines based on carbon skeletons and common elements such as nitrogen, oxygen, sulfur and halogens.^1^ The discovery and FDA approval of bortezomib, a boronic acid derivative, for multiple myeloma in 1995 and 2003, respectively,^2^ sparked interest in incorporation of non-native elements directly into the framework of organic compounds for drug design. This may be a promising strategy for the development of new drug candidates, thus allowing the expansion of the medicinal chemistry space. The isosteric exchange of carbon with silicon – known as the “carbon–silicon switch” – has emerged as one of the most frequently exploited pathways.^3–6^ The similarity of silicon to carbon makes it the ideal choice as a carbon bioisostere, while some differences between these elements lead to marked changes of the physico-chemical and pharmacological properties of the resulting sila-drugs.^7^ The differences are related to atomic size (covalent radii of 111 pm for Si and 67 pm for C), bond length (1.87 Å for the Si–C bond compared to 1.54 Å for the C–C bond), electronegativity (1.7 for Si *vs.* 2.5 for C) and silicon's ability to adopt a higher coordination number (up to 6) compared to carbon that can lead to changes in structural stability, bioavailability, interactions with biological targets, and ultimately, therapeutic efficacy.^8,9^ The increased lipophilicity, which is closely related to the specific molecular structure of silicon-containing compounds, enhances their cellular penetration capacity. Moreover, no “element-specific” intrinsic toxicity has been documented in the literature for small organosilicon molecules.^10,11^

An easy strategy to include Si in the drug structure consists of the attachment of the trimethylsilyl (TMS) group to a biologically active scaffold.^3^ Some systematic studies to elucidate the impact of different silyl groups on the anticancer activity of mucobromic acid (MBA) bearing a furan-2(5*H*)-one core^12^ have shown that the attachment of Si-containing fragments can increase the stability of the compounds, improve their pharmacokinetic profile and decrease their toxicity.^13^ Similarly, the introduction of the TMS group into camptothecin, a DNA topoisomerase inhibitor, significantly enhanced its anticancer activity, even against multi-drug resistant cells.^14^

Recently, we reported on the antiproliferative activity and insights into the underlying mechanisms of action of a series of Schiff bases derived from four-ring heterocyclic scaffolds, namely indoloquinolines, indolobenzazepines, indolobenzazocines and indolobenzazonines, which differ by the size of the central N-containing ring, varying from 6-membered to 9-membered.^15–25^ In continuation of these studies, we aimed to perform (i) the synthesis of a TMS-substituted Schiff base proligand derived from a four-ring heterocyclic scaffold, (ii) the preparation of Cu(ii) and Zn(ii) complexes with these biologically active proligands and (iii) the investigation of physical and spectroscopic properties, solution stability, lipophilicity and anticancer potential of the compounds prepared.

**Scheme 1:**
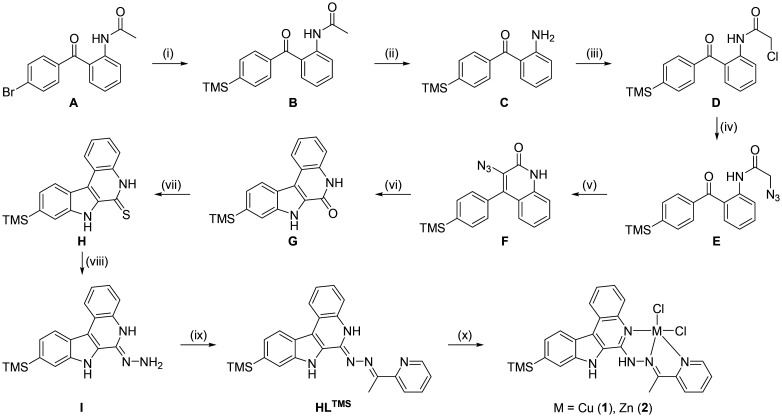
Synthetic pathway to HL^TMS^ and Cu(ii) and Zn(ii) complexes with HL^TMS^. Reagents and conditions: (i) Pd_2_dba_3_, JohnPhos, KF_dry_, (TMS)_2 degassed_, DMPU_degassed_, H_2_O_degassed_, 100 °C, 2 h; (ii) acetone, 6 M HCl, 70 °C, 5 h; (iii) chloroacetyl chloride, chloroform, reflux, 1 h; (iv) sodium azide, dimethyl formamide, 60 °C, 1 h; (v) ethanol, aq. 40% NaOH, 60 °C, 5 h; (vi) toluene, 130 °C, 2 h; (vii) Lawesson's reagent, toluene, 110 °C, 16 h; (viii) hydrazine hydrate, 135 °C, 20 h; (ix) 2-acetylpyridine, methanol, reflux, 16 h; (x) CuCl_2_·2H_2_O or ZnCl_2_·2H_2_O, methanol, reflux, 30 min.

Herein we report on the multistep synthesis of the 9-trimethylsilylindolo[2,3-*c*]quinoline-derived Schiff base HL^TMS^ and on the preparation of copper(ii) and zinc(ii) complexes **Cu(HL**^**TMS**^**)Cl**_**2**_ (1) and **Zn(HL**^**TMS**^**)Cl**_**2**_ (2) (see Scheme 1). The compounds were characterized by elemental analysis, ESI mass spectrometry, IR, UV-vis, ^1^H and ^13^C NMR (for HL^TMS^ and G) spectroscopy, single crystal X-ray diffraction for 1 and electron diffraction (ED) for 2 and stability in solution. In addition, their cytotoxic activity was screened in a panel of 60 cancer cell lines at the National Cancer Institute (NCI) of the National Institutes of Health (NIH), and also tested on A549 and MCF-7 tumor cells in one of our laboratories to determine their IC_50_ values and elucidate their effect on the mitochondrial membrane potential.

## Results and discussion

### Synthesis of HL^TMS^

The 9-trimethylsilylindolo[2,3-*c*]quinoline-derived Schiff base HL^TMS^ was prepared by following the multistep synthetic pathway, as shown in Scheme 1, by adapting protocols reported previously.^26–28^ Species A was obtained in 88% yield by the reaction of acetanilide, Pd(OAc)_2_ and sodium dodecyl sulfate in water with *t*-butyl peroxide, 4-bromobenzaldehyde and trifluoroacetic acid at room temperature for 24 h. Silylation of species A using tris-(dibenzylideneacetone)dipalladium(0) (Pd_2_dba_3_) *via* a 2-(di-*t*-butylphosphino)biphenyl (JohnPhos)/KF catalyzed reaction in *N*,*N*′-dimethylpropyleneurea (DMPU) with small amounts of water and hexamethyldisilane at 100 °C for 2 h afforded species B in 40% yield. The TMS-substituted compound B was further deprotected with the liberation of compound C*via* reaction with 6 M HCl in acetone. 2-Amino-4′-trimethylsilylbenzophenone C was reacted further with chloroacetyl chloride to give the chloroacetylated species D in 58% yield. The chloro substituent in D was then replaced almost quantitatively by an azido group with the formation of species E. The base-catalyzed cyclization reaction of E resulted in azido-phenylquinolinone F in 70% yield. In a second cyclization step, species F, which was not stable at high temperature, was converted into the expected 9-trimethylsilylindolo[2,3-*c*]quinoline-2(1*H*)-one G with the release of nitrogen in 79% yield. Quantitative thionation of lactam species G in thiolactam H was realized by prolonged heating with Lawesson's reagent in toluene. Hydrazone I was obtained by reaction of H with hydrazine hydrate used as the reagent and solvent with 88% yield. Finally, Schiff base condensation of hydrazone I with 2-acetylpyridine in boiling methanol overnight afforded HL^TMS^ in 77% yield. The NMR atom numbering scheme is shown in Fig. S1 in the ESI.† The ^1^H NMR spectra of species A, B, D, E, F and G are shown in Fig. S2–S7,† and the ^13^C and ^29^Si NMR spectra of species G are shown in Fig. S8 and S9.† The ^1^H and ^13^C NMR spectra of HL^TMS^, shown in Fig. S10 and S11,† were in accord with the expected structure. The 25 proton resonances registered in the spectrum are in accordance with the *C*_1_ molecular symmetry of HL^TMS^. There are no other isomeric species in solution. The presence of the TMS group is seen at 0.35 ppm, the methyl group at the Schiff base C=N group at 2.68 ppm, while the two NH protons, one at the indole nitrogen and another at the nitrogen atom of the quinoline moiety resonate at 11.93 and 10.77 ppm, respectively. The ESI mass spectra of species B, C, D, F, G and HL^TMS^ are shown in Fig. S12–S18.† The positive ion ESI mass spectrum of HL^TMS^ showed two peaks with an *m*/*z* of 424.25 and 847.29, which could be assigned to [M + H]^+^ (calcd *m*/*z* 424.20) and to the dimeric associate [2M + H]^+^ (calcd *m*/*z* 847.38). In the negative ion ESI mass spectrum of HL^TMS^, a peak with an *m*/*z* of 422.08 was attributed to [M − H^+^]^−^ (calcd *m*/*z* 422.18).

### Synthesis of copper(ii) and zinc(ii) complexes

The complexes **Cu(HL**^**TMS**^**)Cl**_**2**_ (1) and **Zn(HL**^**TMS**^**)Cl**_**2**_·**2H**_**2**_**O** (2) were prepared in quantitative yields by reactions of the corresponding metal chlorides with HL^TMS^ in boiling methanol. The ^1^H and ^13^C NMR spectra of Zn(ii) complex 2 became interpretable only after keeping for one week at room temperature in an NMR tube and were in agreement with its *C*_1_ molecular structure (Fig. S19 and S20†). The ESI(+) mass spectrum of 1 (Fig. S21†) showed a strong peak with an *m*/*z* of 521.13 and another one with an *m*/*z* of 1043.13, which were assigned to [M − Cl]^+^ (calcd *m*/*z* 521.10) and to a dimeric associate [(M − Cl)_2_ − H^+^]^+^ (calcd *m*/*z* 1043.18). A lower intensity peak with an *m*/*z* of 485.16 was attributed to [M − 2Cl − H]^+^. In the ESI(–) mass spectrum of 1 (Fig. S22†), the peak with an *m*/*z* of 519.05 was assigned to [M − Cl − 2H]^−^ (calcd *m*/*z* 519.08). The ESI(+) mass spectrum of complex 2 (Fig. S23†) showed a peak with an *m*/*z* of 522.12 which was assigned to [M − Cl]^+^ (calcd *m*/*z* 522.09) and two remarkable peaks, one very strong with an *m*/*z* of 909.34 and the second with an *m*/*z* of 455.18. The first one could be assigned to [Zn(HL^TMS^)(L^TMS^)]^+^ (calcd *m*/*z* 909.30) and the second to the doubly charged ion [Zn(HL^TMS^)_2_]^2+^ (calcd *m*/*z* 455.15). Zn(ii) to HL^TMS^ species at a ratio of 1 : 2 were formed in the mass spectrometer. The ESI(–) mass spectrum of 2 (Fig. S24†) contains two peaks with an *m*/*z* of 558.02 attributed to [Zn(L^TMS^)Cl_2_]^−^ (calcd *m*/*z* 558.05) and an *m*/*z* of 1116.91 assigned to the dimeric associate [{Zn(HL^TMS^)Cl_2_}{Zn(L^TMS^)Cl_2_}]^−^ (calcd *m*/*z* 1117.10). It should also be noted that all experimental isotopic patterns were in accordance with the calculated isotopic distributions for the assigned ions both in positive and negative ion modes.

### Molecular structures of complexes 1 and 2

**Fig. 1:**
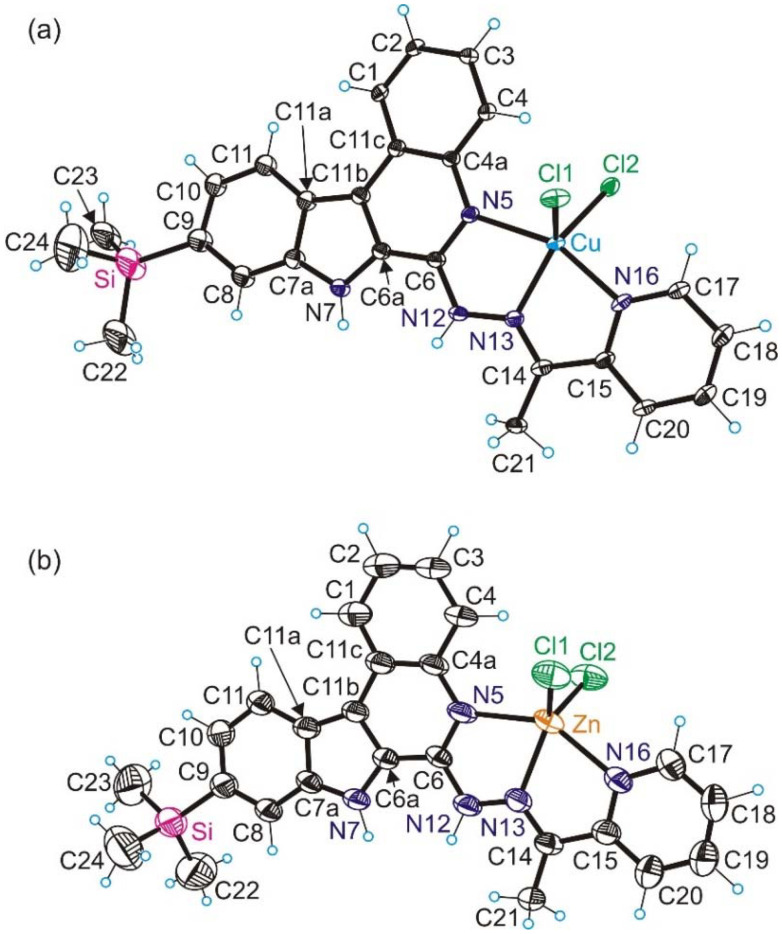
(a) ORTEP view of the X-ray diffraction structure of 1 with thermal ellipsoids at the 50% probability level (only one position of the disordered molecule is shown); (b) electron diffraction (ED) structure of complex 2 with thermal ellipsoids at the 40% probability level.

The results of single crystal X-ray diffraction (SC-XRD) measurements of complex 1 and electron diffraction (ED) measurements of 2 are shown in Fig. 1. The details of data collection and refinement are summarized in Table S1 in the ESI.† Complex 1 crystallized in the monoclinic centrosymmetric space group *P*2_1_/*c*, while 2 crystallized in the centrosymmetric triclinic space group *P*1̄. Pertinent metric parameters for complexes 1 and 2 are shown in Table 1.

**Table 1:** Selected bond distances (Å) in 1 and 2

Bond lengths (Å)	1	2
M–N5	2.020(14)	2.240(8)
M–N13	1.984(5)	2.144(7)
M–N16	2.026(5)	2.258(10)
M–Cl1	2.4493(2)	2.271(4)
M–Cl2	2.251(2)	2.264(4)
*τ*_5_ a	0.34/0.35^b^	0.35

a *τ*_5_ = 0 for square-pyramidal and 1 for trigonal–bipyramidal coordination geometry.

b Two positions of N5 due to disorder were taken into account.

Complexes 1 and 2 adopt a strongly distorted square-pyramidal coordination geometry (see the *τ*_5_-parameter in Table 1).^29^ The Schiff base HL^TMS^ acts as a neutral tridentate ligand binding to copper(ii) and zinc(ii) *via* the quinoline moiety nitrogen atom N5, Schiff base nitrogen atom N13 and pyridine nitrogen atom N16, and together with the two chlorido co-ligands Cl1 and Cl2 forms the coordination polyhedron.

### Stability, solubility and lipophilicity of HL^TMS^ and complexes 1 and 2 in aqueous solution and their fluorescence properties

**Fig. 2:**
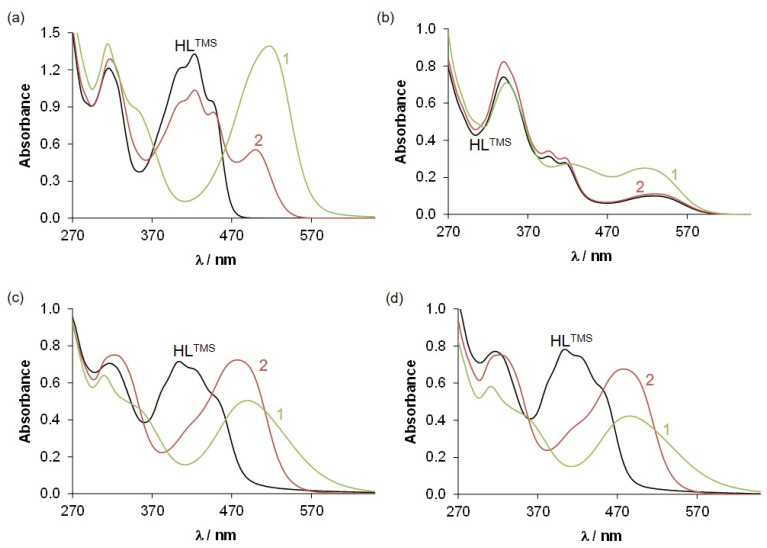
UV-vis absorption spectra recorded for HL^TMS^ and its complexes (1, 2) after dissolution (a) in DMSO and at pH (b) 2.0, (c) 7.4 and (d) 11.7 {*c* = 50 μM; *ℓ* = 1 cm; *T* = 25.0 °C}.

The aqueous stability of HL^TMS^ and its metal complexes 1 and 2 was explored by UV-vis spectroscopy at pH 2.0, 7.4 and 11.7. The stability in DMSO was also studied since the stock solutions were prepared in this solvent due to their limited aqueous solubility. HL^TMS^ was found to be stable in DMSO at least for 19 h (Fig. S25†). In aqueous solutions, slight changes of the UV-vis absorption spectra could be observed within a few hours, most probably due to the partial precipitation of HL^TMS^ since the shape of the spectra did not change but only the absorption decreased with time. It is worth noting that no photostability issues were observed during measurements with a fiber optic equipped spectrophotometer operating with a Xe flash lamp. However, irradiation with other light sources, such as a diode array UV-vis photometer or a Xe lamp fluorimeter, induced remarkable spectral changes (Fig. S25†). To avoid photochemical reactions, the fiber optic UV-vis instrumental setup was used and particular attention was paid to the implementation of subsequent fluorometric measurements (see details in the Experimental section). As shown in Fig. 2, the UV-vis spectra recorded for HL^TMS^ at pH 7.4 and 11.7 are practically the same, while this was not the case for the spectrum measured at pH 2.

Complexes 1 and 2 were also proved to be stable in DMSO for at least 19 h (Fig. S26 and S27†). In addition, the UV-vis spectra were recorded in tetrahydrofuran to confirm that DMSO, as the solvent, does not affect the inner coordination sphere of the metal complexes (Fig. S28†). Complex 1 was also stable in aqueous solution at pH 7.4 (10 mM HEPES) but showed spectral changes under acidic and basic conditions within 19.4 and 5.8 h, respectively. Schiff bases are sensitive to hydrolysis, especially in the presence of certain metal ions. We suppose that slow hydrolytic processes occur with 1. In contrast, complex 2 displayed only minor spectral changes in aqueous solutions (Fig. S27†). Taking a closer look at Fig. 2b, the UV-vis spectrum of the Zn(ii) complex at pH 2 is shown to be practically the same as that of HL^TMS^, namely complex 2 dissociates completely under such acidic conditions. At pH 7.4, 1 and 2 retain their integrity and show no considerable dissociation even at pH 11.7. Thus, the predominant species at pH 7.4 for complex 1 is [**Cu(L**^**TMS**^**)**]^+^.

Due to the low aqueous solubility and high lipophilicity of the compounds, the partition constant (*P*) could not be determined using the traditional *n*-octanol/water partitioning shake-flask method. The SwissADME tool^30^ was applied to predict the log *P* values for HL^TMS^ and its non-substituted analogue HL^H^, as well as for complexes 1 and 2 and Cu(ii) and Zn(ii) complexes with **HL**^**H**^ (Table 2). The predictions suggest high log *P* values for all compounds, and the TMS group enhances the lipophilicity of both the proligand and metal complexes.

**Table 2:** Predicted log *P* values for proligands and metal complexes

Compound	HL^TMS^	HL^H^	Cu(L^TMS^)Cl_2_	Zn(L^TMS^)Cl_2_	Cu(L^H^)Cl_2_	Zn(L^H^)Cl_2_
log *P*	+3.51	+2.94	+2.95	+2.96	+2.40	+2.40

**Fig. 3:**
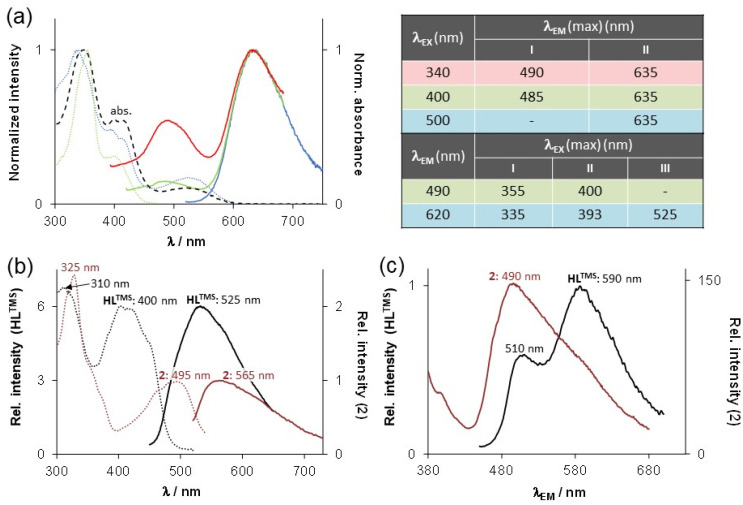
Fluorescence emission (solid line) and excitation (dotted line) spectra of (a) HL^TMS^ at pH 2.0 with the corresponding parameters listed in the table; (b) HL^TMS^ and 2 at pH 7.4; and (c) solid form HL^TMS^ and 2. The dashed line in (a) is the normalized UV-vis absorption spectrum of HL^TMS^ at pH 2.0. Numbers I–III in the table denote the respective emission or excitation maxima, and color-coding of the lines corresponds to the colors of the spectra in (a); {*c* = 5 μM (a and b), *T* = 25.0 °C}.

The fluorescence properties of HL^TMS^ and the two metal complexes 1 and 2 were studied in aqueous solution at pH 7.4 and pH 2.0. Due to the photosensitivity of HL^TMS^, special precautions listed in the Experimental section were taken in order to obtain reasonable spectral data. The fluorescence properties of the crystalline samples were also studied. Complex 1 is not fluorescent in both aqueous solution and in the solid state due to the quenching effect of the paramagnetic Cu(ii) ion (d^9^, *S* = ½). HL^TMS^ exhibits moderate fluorescence at both pH 7.4 and pH 2.0. The molecule can be excited in the visible wavelength range (*λ*_EX_ (max) = 400 nm (pH 7.4) and 525 nm (pH 2.0)), which is an advantageous feature for fluorescence microscopy. Interestingly, under acidic conditions, HL^TMS^ behaves against the expectations that the same fluorescence emission spectrum is generally observed regardless of the excitation wavelength (Kasha's rule) (Fig. 3a).^31^ This is likely due to the presence of two isomeric fluorescent species in solution. We assume that these might be the exocyclic (C^6^=N^12^) and endocyclic (C^6^=N^5^) tautomers.

Also the excitation spectra were different for the two distinct emission bands and the characteristic excitation bands of the two species are recognisable in the absorption spectrum of HL^TMS^ at the same pH. As expected, at pH 2, the fluorescence profile of 2 closely resembled that of HL^TMS^, as the complex dissociated under strong acidic conditions. At pH 7.4, the behaviors of both HL^TMS^ and 2 are different (Fig. 3b). The excitation spectra of HL^TMS^ and 2 are very similar to the UV-vis absorption spectra of the respective compound at the same pH. The emission maximum of 2 is red shifted compared to HL^TMS^, and both species emit light in the visible range. Both solid samples are also fluorescent. HL^TMS^ shows two emission bands and this compound can be excited over a wide range (*λ*_EX_ = 250–450 nm), while 2 emits at somewhat shorter wavelengths and is excitable between 250–470 nm (Fig. 3c).

### Cell-free ROS generation and redox activity

**Fig. 4:**
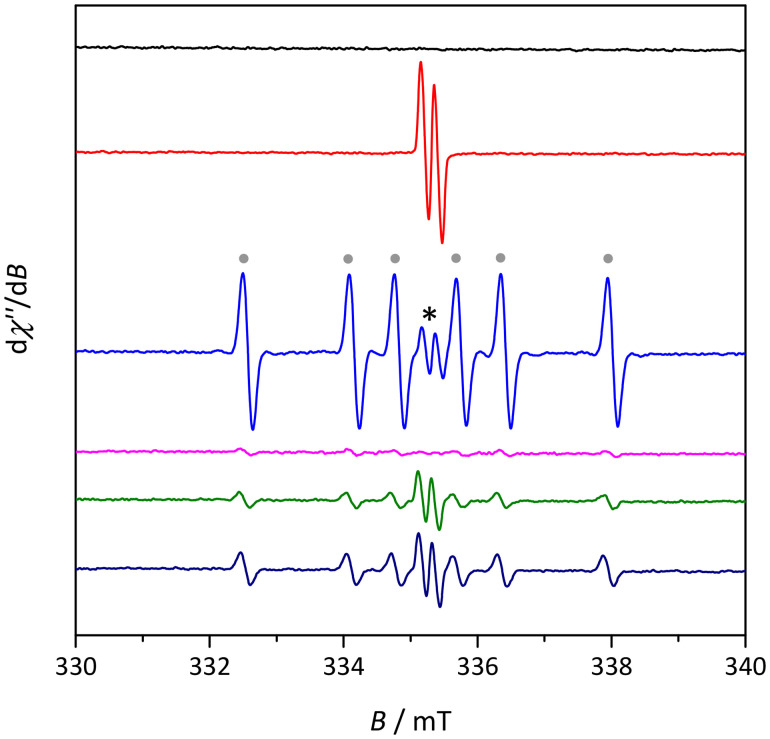
Experimental EPR spectra measured for 50% (v/v) DMSO/H_2_O mixture/DMPO (black trace), 1/Asc (red trace), 1/DMPO/Asc/H_2_O_2_ (blue trace), 1/DMPO/H_2_O_2_ (magenta trace), 2/DMPO/Asc/H_2_O_2_ (green trace) and HL^TMS^/DMPO/Asc/H_2_O_2_ (dark blue trace) systems under air in a 50% (*v*/*v*) DMSO/H_2_O mixture. Initial concentrations: *c*(sample) = 7 μM, *c*(DMPO) = 40 mM, *c*(H_2_O_2_) = 100 mM. The EPR spectrum of ˙DMPO-CH_3_ adducts is marked with circles (as is the case for the blue trace) and of ascorbyl radical Asc˙ with an asterisk.

**Fig. 5:**
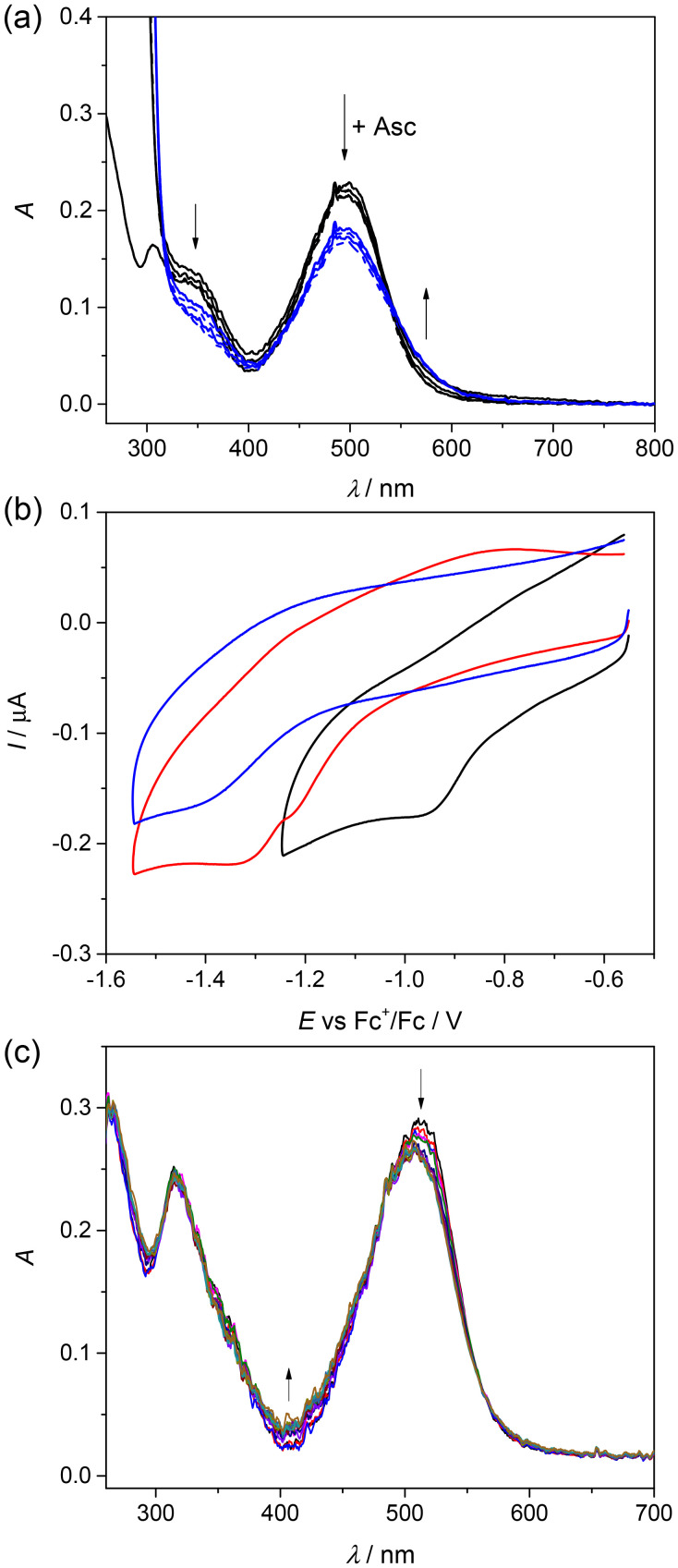
(a) Changes in the UV-vis spectra of 1 in a 50% (v/v) DMSO/H_2_O mixture measured in 10 s intervals after addition of 10 equiv. (black lines) and 30 equiv. (blue lines) of Asc; (b) cyclic voltammograms of 1 (black trace), 2 (red trace) and HL^TMS^ (blue trace) in DMSO/0.1 M *n*Bu_4_NPF_6_ on a Pt disc working electrode, scan rate 100 mV s^−1^; (c) a decrease of the absorption band at 516 nm was observed upon cathodic reduction of 1 in DMSO/0.2 M *n*Bu_4_NPF_6_ at the first reduction peak when a honeycomb microstructured Pt working electrode was used. For the UV-vis spectra of HL^TMS^, 1 and 2, see Fig. 2.

The ability of HL^TMS^, 1 and 2 to generate ROS in solution in the presence of H_2_O_2_ was studied by the indirect technique of electron paramagnetic resonance (EPR) spectroscopy and spin trapping technique. The investigated samples were dissolved in a 50% (v/v) mixture of DMSO/H_2_O and mixed with the spin trapping agent 5,5-dimethyl-1-pyrroline *N*-oxide (DMPO) in air. No EPR signal was observed for the DMSO/H_2_O/DMPO mixture as a reference (black trace in Fig. 4). First, the ability of 1 to generate ROS was investigated in the presence of ascorbate (Asc) as a natural reducing agent, which was added to the reaction mixture in the absence and presence of DMPO. The red trace in Fig. 4 represents the EPR spectrum measured 2 min after the addition of an excess of Asc to the solution of 1 in the absence of DMPO. This signal can be unambiguously attributed to the ascorbyl radical Asc˙ (*a*_H_ = 0.177 mT, *g* = 2.0054) formed by the reduction of 1 with Asc. Changes in the UV-vis spectra of 1 were observed after the addition of Asc to Cu(ii) complex 1 dissolved in the DMSO/H_2_O mixture, providing further evidence for the reaction of Asc with this complex (Fig. 5a).

Since the formation of hydroxyl radicals *via* the Fenton-type reaction requires H_2_O_2_, the DMSO/H_2_O solutions of HL^TMS^, 1 and 2 were mixed with DMPO as the spin trapping agent under aerobic conditions and the EPR spectra were recorded after the addition of Asc and H_2_O_2_. The EPR spectra of the prepared reaction mixture 1/DMPO/Asc were recorded 2 min after the addition of H_2_O_2_ to the system. As seen in Fig. 4, complex 1 induced strong ROS generation along with the formation of reactive radical intermediates, as evidenced by the presence of the dominating six-line EPR signal (blue trace in Fig. 4) in the *in vitro* cell-free measurement. This EPR signal can be assigned to the spin-adduct ˙DMPO-CH_3_ (*a*_N_ = 1.59 mT, *a*_H_^β^ = 2.28 mT; *g* = 2.0055), originating from the reactions of ROS with DMSO. The rapid reaction of hydroxyl radicals with DMSO produced methyl radicals ˙CH_3_, detectable *via* the reaction with DMPO as the corresponding carbon-centered spin-adduct. It should be noted that much smaller amounts of radicals were formed in the absence of Asc (see the magenta trace in Fig. 4) indicating the important role of the natural reducing agents to produce ROS.

No EPR signal was observed for the DMSO/H_2_O/DMPO/H_2_O_2_ mixture as a reference (not shown). For Zn(ii) complex 2 and the corresponding ligand HL^TMS^, the formation of both ascorbyl radicals Asc˙ and ˙DMPO-CH_3_ spin adducts was observed in the presence of Asc and H_2_O_2_ (see the green and dark blue traces, respectively, in Fig. 4). However, the amount of spin adducts is substantially smaller, which indicates the important role of the metal redox-activity for ROS generation.

The redox activity of all samples was tested by cyclic voltammetry in DMSO (due to the low aqueous solubility). All three samples were redox active in the cathodic part, as shown in Fig. 5b. The easiest reduction was confirmed for Cu(ii) complex 1 (*E*_pc_ = –0.96 V *vs.* Fc^+^/Fc), while the most negative reduction potential was observed for the corresponding proligand HL^TMS^ (*E*_pc_ = –1.42 V *vs.* Fc^+^/Fc). The Zn(ii) complex 2 also exhibited peaks at more negative reduction potentials (*E*_pc_ = –1.23 V and −1.33 V *vs.* Fc^+^/Fc) compared to Cu(ii) complex 1; however, this redox activity is most probably ligand-centered. A decrease of the absorption band at 516 nm was observed during the cathodic reduction of 1 in DMSO/0.2 M *n*Bu_4_NPF_6_ (Fig. 5c) similar to that observed in the experiments with sodium ascorbate.

### Photoinduced processes monitored by the EPR spin trapping technique

**Fig. 6:**
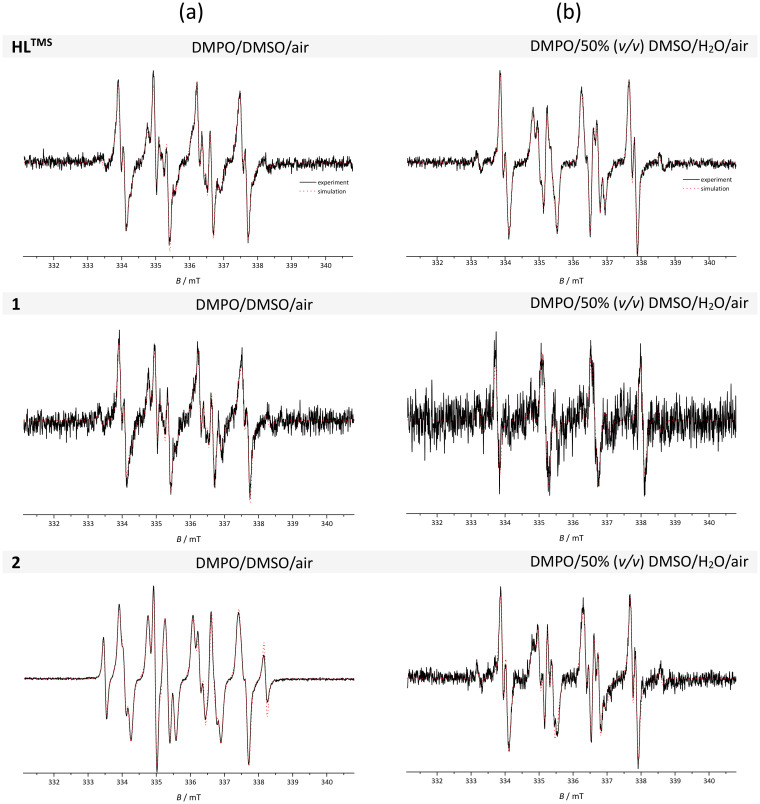
Normalized experimental (black solid line) and simulated (red dotted line) EPR spectra obtained after LED@365 nm exposure (radiation dose 5.4 J cm^−2^) of aerated solutions of HL^TMS^, 1 and 2 in (a) DMSO and (b) 50% (v/v) DMSO/H_2_O mixture in the presence of the DMPO spin trap. Initial concentrations: *c*(sample) = 33 μM and *c*(DMPO) = 35 mM.

Molecules with a quinoline-based scaffold can behave as photosensitizers,^32–34^ so attention was also focused on the examination of the behavior of the compounds reported herein upon UV exposure (LED@365 nm) utilizing the EPR spin trapping technique. Fig. S34a–c† show the time-courses of the EPR spectra monitored upon LED@365 nm exposure for HL^TMS^, 1 and 2 in aerated DMSO solution in the presence of the DMPO spin trap. In all studied systems, the generation of EPR signals of the corresponding spin-adducts of various intensities upon exposure was detected. The photoactivation of the HL^TMS^/DMPO/DMSO/air system led to the generation of several spin-adducts, namely ˙DMPO-O_2_^−^/OOH (relative concentration, *c*_rel_ = 45%), ˙DMPO-OCH_3_ (*c*_rel_ = 34%), ˙DMPO-OR (*c*_rel_ = 15%) and ˙DMPO-CR* (*c*_rel_ = 7%) as shown in Fig. 6a. The presence of ˙DMPO-O_2_^−^/OOH in the system indicates the interaction of the excited states of HL^TMS^ with molecular oxygen *via* the photoinduced electron transfer process (photooxidation I process), forming the superoxide radical anion. The second major spin-adduct, ˙DMPO-OCH_3_, is attributed to the methoxy radical ˙OCH_3_, added on the DMPO moiety, which may be formed *via* several pathways. The photoexcited ligand or superoxide radical anion interacts with the DMSO molecule affording the methyl radical ˙CH_3_, which immediately reacts with molecular oxygen to give the unstable ˙OOCH_3_ radical. This species is highly reactive, and quickly disproportionates to the methoxy radical with the consequent addition on the DMPO spin trap or reaction with DMPO with the formation of transient ˙DMPO-OOCH_3_, which rapidly transforms into ˙DMPO-OCH_3_. The two other spin-adducts ˙DMPO-OR and ˙DMPO-CR* are assigned to paramagnetic species added on DMPO and generated *via* interaction with DMSO.^35,36^

The spin Hamiltonian parameters, the hyperfine coupling constants (*hfcc*) and *g*-values obtained from the simulation analysis are summarized in Table 3.

**Table 3:** Hfcc and *g*-values of the DMPO spin-adducts elucidated from the simulations of experimental spectra

Spin-adduct	*a*_N_ (mT)	*a*_H_^β^ (mT)	*a*_H_^γ^ (mT)	*g*-Value
**DMSO**				
˙DMPO-O_2_^−^	1.283 ± 0.006	1.035 ± 0.010	0.140 ± 0.001	2.0058
˙DMPO-OCH_3_	1.321 ± 0.004	0.785 ± 0.005	0.166 ± 0.006	2.0058
˙DMPO-OR	1.194 ± 0.062	1.465 ± 0.033	—	2.0058
˙DMPO-CR*	1.483 ± 0.019	1.844 ± 0.054	—	2.0057
**50% (v/v) DMSO : H**_**2**_ **O**				
˙DMPO-O_2_^−^	1.137 ± 0.005	1.086 ± 0.001	0.131 ± 0.004	2.0059
˙DMPO-OR	1.412 ± 0.004	0.968 ± 0.020	0.152 ± 0.004	2.0059
˙DMPO-OH	1.460 ± 0.005	1.359 ± 0.005	0.043 ± 0.005	2.0059
˙DMPO-CH_3_	1.570 ± 0.040	2.200 ± 0.011	—	2.0058

The photoexcitation of complex 1 in aerated DMPO/DMSO solution led to the generation of the same species (˙DMPO-O_2_^−^/OOH, *c*_rel_ = 65%; ˙DMPO-OCH_3_, *c*_rel_ = 27%; ˙DMPO-OR, *c*_rel_ = 3%; ˙DMPO-CR*, *c*_rel_ = 7%), but the intensity of the EPR signal is significantly lower. Here the concurrent reaction – reduction of Cu(ii) *via* photoinduced electron – may occur, causing the decrease of the EPR signal.^33^ This is also supported by fluorescence experiments, where due to the quenching effect of the paramagnetic Cu(ii) ion, complex 1 was found to be not fluorescent. The LED@365 nm exposure of the aerated reaction system 2/DMPO/DMSO resulted in the immediate formation of several spin-adducts (Fig. 6 and Fig. S29c†). The dominant species formed is ˙DMPO-OCH_3_ (*c*_rel_ = 61%), and the other species are attributed to ˙DMPO-O_2_^−^/OOH (*c*_rel_ = 30%) and ˙DMPO-CR* (*c*_rel_ = 9%). Here we suggest the main pathway *via* reactions of the photoexcited complex with DMSO to afford the methyl radical ˙CH_3_ (with the following cascade reaction to ˙DMPO-OCH_3_) and the dimsyl radical ˙CH_2_(S=O)CH_3_, which is added to DMPO as ˙DMPO-CR*.^36^ This also correlates well with the relatively high fluorescence activity of 2 as was discussed above. The analogous experiments were performed also in the mixed solvent 50% (v/v) DMSO** **:** **H_2_O. The intensity of EPR signals was significantly lower in comparison with the spectra obtained in pure DMSO (Fig. 6b) and this may be caused by the decomposition of the superoxide radical anion, as well as by the decreased stability of the spin-adduct ˙DMPO-O_2_^−^/OOH in the presence of water.^33,35^ In the HL^TMS^ and complex 2 reaction system upon LED@365 nm exposure, the following radicals are present: ˙DMPO-O_2_^−^/OOH (*c*_rel_ ≈ 50%) and ˙DMPO-OCH_3_ (*c*_rel_ ≈ 47%) and the spin-adduct attributed to ˙DMPO-CH_3_ in a minor concentration (*c*_rel_ = 3%; the *hfcc* and *g*-values are presented in Table 3). The situation was different in the presence of complex 1, where in the reaction system the dominant species was ˙DMPO-OH (*c*_rel_ = 77%). The increased formation of the hydroxyl radical spin-adduct agrees well with the photodecomposition of hydrogen peroxide formed *via* superoxide radical anion dismutation as well as the complex reaction mechanism of Cu(ii) with H_2_O_2_.^34^ Additionally, the reaction of DMSO with hydroxyl radicals generates methyl radicals, detected as the low-intensity six-line signal of the ˙DMPO-CH_3_ spin-adduct.^33^ These observations confirmed the photochemical activity of the indolo[2,3-*c*]quinoline ligand HL^TMS^ and its metal complexes 1 and 2, indicating that they are potential candidates as photosensitizers for photodynamic therapy.^37^

### Cytotoxicity assays

The anticancer potential of the proligand HL^TMS^ and metal complexes 1 and 2 was evaluated along with the selectivity towards specific types of cancers: leukemia, non-small cell lung (NSCLC), colon, central nervous system (CNS), melanoma, ovarian, renal, prostate and breast cancers, by the National Cancer Institute's Developmental Therapeutics Program. This study involved the NCI 60 human tumor cell line panel.^38^ One-dose (10 μM) assays indicate that HL^TMS^, as well as complexes 1 and 2, are highly cytotoxic (see Fig. S30–S32 in the ESI†). Five-dose (0.01 μM–100 μM) concentrations were applied for all three compounds and the data were used to calculate the 50% growth inhibition values of the tested cells (GI_50_), the total growth inhibition (TGI), and lethal dose concentration inducing 50% cell death (LC_50_). The GI_50_ data in μM are presented in Table 4. The 5-dose screen curves and mean graphs for HL^TMS^, 1 and 2 are shown in Fig. S33–S38 in the ESI.†

**Table 4:** GI_50_ concentrations for HL^TMS^, 1 and 2 across the NCI 60 human cancer cell line panel accompanied by heat map data

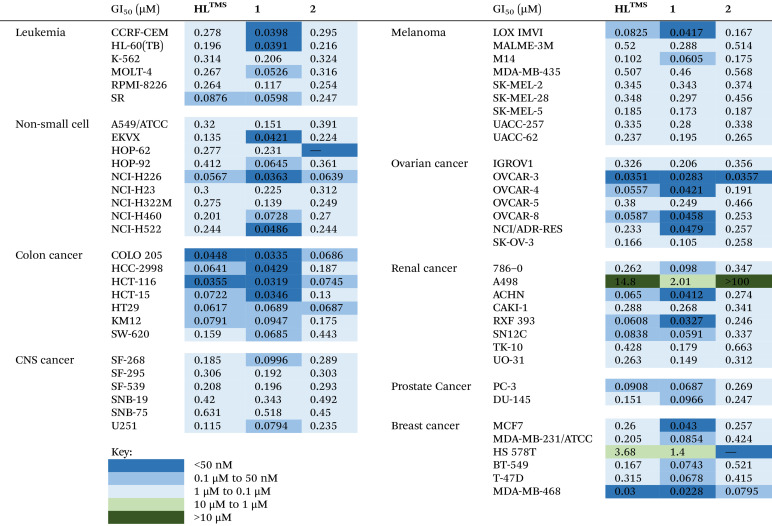

The results indicate that all three compounds HL^TMS^, 1 and 2 exhibit strong inhibitory effects on cancer cell growth against the majority of cell lines within the panel. For the most active compound **Cu(HL**^**TMS**^**)Cl**_**2**_ (1), the average GI_50_, TGI, and LC_50_ concentrations across all cell lines in the panel were 0.181 μM, 3.07 μM, and 13.9 μM, respectively. For comparison, the Schiff base HL^TMS^ was less active with the respective values of 0.518 μM, 12.1 μM and 63.9 μM, indicating that the two species might have distinct mechanisms of action.

A comparison of the GI_50_ values for different types of cancer cells indicates that the colon cancer cell lines, four of seven ovarian cancer cell lines, the non-small-cell lung cancer (NSCLC) cells and the central nervous system (CNS) tumor cells are the most sensitive to drug 1. In comparison, the renal cancer cell line A498 and the breast cancer cell line HS578T are the most resistant to all three compounds HL^TMS^, 1 and 2. In most of the cases, complex formation with Zn(ii) decreased or maintained the cytotoxicity with some exceptions (RPMI-8226, HOP-92, NCI-H322M, SNB-75 and MALME-3M), while complex formation with Cu(ii) enhanced or preserved the antiproliferative activity exhibited by HL^TMS^ in all cell lines tested, except HT29 and KM12.

**Fig. 7:**
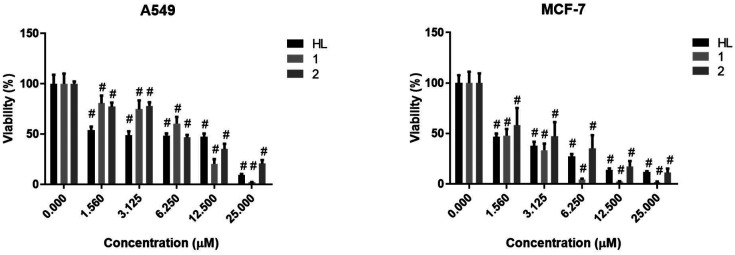
Cell viability of A549 and MCF-7 adenocarcinoma cells upon HL^TMS^, complex 1, and complex 2 treatments. Two-way ANOVA, Dunnett's multiple comparisons test, *P* value: ^#^ <0.0001.

In order to select the appropriate concentration of the Schiff base HL^TMS^, its Cu(ii) complex 1 and Zn(ii) complex 2 for the subsequent biological experiments, first we measured the viability of A549 and MCF-7 human tumor cells using the (3-[4,5-dimethylthiazol-2-yl]-2,5-diphenyl tetrazolium bromide (MTT)) assay upon treatments. Fig. 7 shows that HL^TMS^, Cu(ii) complex 1, and Zn(ii) complex 2 all exhibited high toxicity on both tumor cells at each tested concentration since they decreased significantly the viability of A549 and MCF-7 cells compared to the untreated control samples. At lower concentrations, HL^TMS^ was more or similarly toxic to its metal complexes; however, at higher concentrations, complex 1 showed significantly elevated toxicity when compared to 2 or HL^TMS^ in A549 and MCF-7 cell lines. According to our results, breast adenocarcinoma MCF-7 cells were more sensitive to the toxic effect of all compounds than A549 cells (Fig. 7). The determined IC_50_ values (μM ± SD) of HL^TMS^ and its complexes 1 and 2 on both cell lines are displayed in Table 5. Cisplatin was used as the positive control, and according to our results, the synthesized Zn(ii) and Cu(ii) complexes are as cytotoxic to A549 and MCF-7 cells as this ‘gold standard’ drug.

**Table 5:** IC_50_ values (μM ± SD) of the Schiff base HL^TMS^ and the complexes 1 and 2 determined on A549 and MCF-7 cancer cell lines and on non-cancerous fibroblast cells. Cisplatin was used as positive control

IC_50_ values (μM)				
Cell lines	HL^TMS^	1	2	Cisplatin
A549	3.23 ± 1.28	6.33 ± 1.08	6.77 ± 1.07	5.39 ± 0.84
MCF-7	1.43 ± 1.08	1.59 ± 1.37	2.57 ± 1.29	2.11 ± 0.50
NIH-3T3	>25	12.58 ± 1.04	>25	n.d.

To evaluate the selectivity of HL^TMS^, 1 and 2 for cancer cells, their cytotoxicity on non-cancerous NIH-3T3 fibroblasts was also tested. The results summarized in Table 5 indicate that the healthy NIH-3T3 cells are less sensitive to HL^TMS^ and 2, since the IC_50_ values were significantly higher than in the case of cancerous cells. Only compound 1 showed some toxicity in the NIH-3T3 cells, but the calculated IC_50_ value in this case again was one magnitude higher or twice as high as the IC_50_ values obtained on MCF-7 and A549 cells, respectively. So, the tested compounds, especially HL^TMS^ and 2, demonstrate cancer cell-selectivity.

### The mechanism of action behind the toxicity of compounds

Since the cell-free *in vitro* tests suggested the possibility of increased production of reactive oxygen species by HL^TMS^, 1 and 2, we aimed to evaluate whether the compounds induce disturbance in mitochondrial function.

**Fig. 8:**
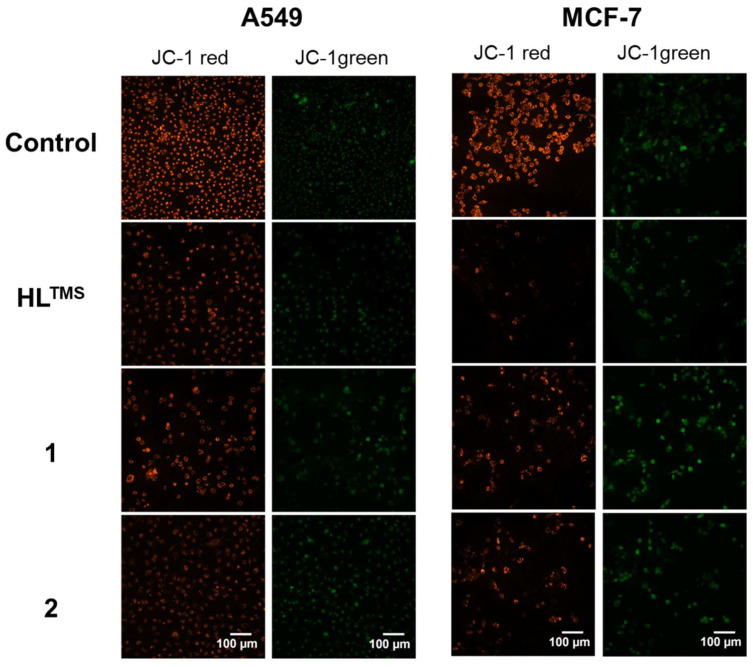
Representative fluorescence images of A549 and MCF-7 cells treated with HL^TMS^, 1 or 2 and stained with JC-1.

**Fig. 9:**
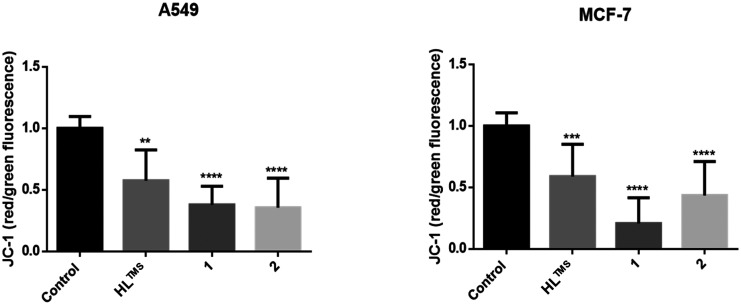
Quantified red and green fluorescence and their ratios indicate the effect of HL^TMS^, 1 and 2 on the mitochondrial membrane potential of tumor cells. One-way ANOVA, Dunnett's multiple comparisons test, *P* value: ** <0.005, *** = 0.001; **** <0.0001.

We monitored the changes in mitochondrial membrane potential by JC-1 staining following treatments with HL^TMS^, 1 and 2 (Fig. 8). The reduction of the red/green fluorescence ratio indicates a compromised mitochondrial membrane potential and thus mitochondrial function damage. As shown in Fig. 9, red/green fluorescence ratios were significantly decreased upon all the treatments compared to the control samples in both A549 and MCF-7 cells. The most significant mitochondrial dysfunction was found in MCF-7 cells exposed to **Cu(HL**^**TMS**^**)Cl**_**2**_ (1).

## Conclusion

We found out that the proligand HL^TMS^ builds strongly distorted square-pyramidal Cu(ii) and Zn(ii) complexes, in which the organic ligands are tridentate, while the other two coordination sites are occupied by two chlorido co-ligands. Even though the SC-XRD and ED structures were of different quality, being in some cases affected by disorder, and measured at different temperatures, one can state that the coordination geometry is actually the same and independent of the metal.

The Zn(ii) complex with HL^TMS^ (2) was found to transform into a complex of 1 : 2 stoichiometry in the mass-spectrometer as evidenced by the presence of peaks attributed to singly charged and doubly charged ions, [Zn(HL^TMS^)(L^TMS^)]^+^ and [Zn(HL^TMS^)_2_]^2+^, respectively. This feature presumably affects also the solution behavior in DMSO, as equilibrium was achieved only in about one week and fully shifted to the complex of 1 : 1 stoichiometry, and the ^1^H and ^13^C NMR spectra became interpretable.

The attachment of TMS to the proligand backbone increased the lipophilicity. The protonation state of HL^TMS^ corresponds to the neutral form at physiological pH, and metal complexes 1 and 2 were also stable at this pH. Both the proligand and complexes were stable in DMSO.

Both HL^TMS^ and 2 were found to generate ROS under cell-free conditions and this was in accordance with their redox activity established by cyclic voltammetry. The disclosed photochemical activity of the indolo[2,3-*c*]quinoline-based proligand HL^TMS^ and its Cu(ii) and Zn(ii) complexes 1 and 2 implies their potential as photosensitizers for photodynamic therapy.

The evaluation of the anticancer potential of HL^TMS^, 1 and 2 in the NCI-60 human tumor cell line panel revealed their strong inhibitory effects against the majority of cell lines. For the most active complex **Cu(HL**^**TMS**^**)Cl**_**2**_ (1), the average GI_50_, TGI and LC_50_ values across all cell lines in the panel (0.181 μM, 3.07 μM, and 13.9 μM, respectively) were superior to those of HL^TMS^, indicating their distinct mechanisms of action and the favorable effect of Cu(ii). With several exceptions, complex formation with Zn(ii) diminished or did not alter the cytotoxicity of HL^TMS^. JC-1 staining following treatments with HL^TMS^, 1 and 2 showed that all compounds induce mitochondrial disturbance of membrane potential contributing to their toxicity.

We can conclude that silylation in combination with the introduction of functional groups enhancing the solubility of both the proligand and metal complexes might allow full exploration of the potential of these kinds of compounds as photosensitizers in photodynamic therapy and investigate their cellular uptake and intracellular localization by fluorescence microscopy.

## Experimental part

### Chemicals

Pd_2_dba_3_, JohnPhos, hexamethyldisilane, DMPU, dimethyl sulfoxide, sodium dodecyl sulfate, CF_3_COOH, Pd(OAc)_2_, CuCl_2_·2H_2_O, and ZnCl_2_·2H_2_O were purchased from commercial suppliers and used as received.

### Synthesis of the Schiff base HL^TMS^ and complexes 1 and 2

#### 2-Acetamido-4′-bromobenzophenone (species A)

Acetanilide (1.1 g, 8.1 mmol), Pd(OAc)_2_ (91 mg, 0.1 mmol) and sodium dodecyl sulfate (117 mg, 0.4 mmol) were suspended in water (25 mL). *t*-Butyl peroxide (70%, 2.24 mL, 16.2 mmol), 4-bromobenzaldehyde (3.0 g, 16.2 mmol) and trifluoroacetic acid (161 μL, 2.03 mmol) were added and the resulting suspension was vigorously stirred at room temperature for 24 h. The mixture was extracted with ethyl acetate (3 × 40 mL) and the combined organic phases were washed with brine (2 × 70 mL). The organic layer was dried over magnesium sulfate and purified on silica using hexane : ethyl acetate 6 : 4 as the eluent to give a white solid, which was further re-crystallized in hexane : ethyl acetate 1 : 1 (45 mL) due to the traces of benzaldehyde as impurity. Yield: 2.29 g, 88%. ^1^H NMR (500 MHz, DMSO-*d*_*6*_), *δ*, ppm: 10.07 (s, 1H), 7.91–7.82 (m, 1H), 7.71 (d, *J* = 8.6 Hz, 3H), 7.61–7.54 (m, 3H), 7.45 (d, *J* = 8.0 Hz, 1H), 7.36 (dd, *J* = 7.7, 1.4 Hz, 1H), 7.26 (td, *J* = 7.6, 1.0 Hz, 1H), 1.76 (s, 3H).

#### 2-Acetamido-4′-trimethylsilylbenzophenone (species B)

A Schlenk flask was charged with 2-acetamido-4′-bromobenzophenone (2.29 g, 7.2 mmol), Pd_2_dba_3_ (198 mg, 0.216 mmol), JohnPhos (387 mg, 1.29 mmol) and dry KF (2.1 g, 36.0 mmol) under an argon atmosphere. Degassed *N*,*N*′-dimethylpropylene urea (freeze and purge, 20 mL) was added and the suspension was stirred for 5 min until it became orange. Then degassed water (freeze and purge, 259 μL, 129.6 mmol) and degassed hexamethyldisilane (freeze and purge, 1.73 mL, 8.64 mmol) were added. The slurry was stirred at 100 °C for 2 h. The suspension was poured onto ethyl acetate (200 mL) and washed thoroughly with water (4 × 100 mL). The organic phase was dried over MgSO_4_ and bound to silica by evaporation of the solvent. The crude product was purified on silica using hexane : ethyl acetate 6 : 4 as the eluent to give a light yellow oil. Yield: 896 mg, 40%. ^1^H NMR (500 MHz, DMSO-*d*_*6*_) *δ*, ppm: 10.05 (s, 1H), 7.65 (d, *J* = 5.3 Hz, 4H), 7.59–7.50 (m, 2H), 7.38–7.34 (m, 1H), 7.27–7.21 (m, 1H), 1.76 (d, *J* = 8.5 Hz, 3H), 0.28 (s, 9H). ESI-MS (acetonitrile/methanol + 1% water), negative: *m*/*z* 309.95 [M–H]^−^ (calcd *m*/*z* for [(C_18_H_21_NO_2_Si − H^+^)]^−^ 310.13).

#### 2-Amino-4′-trimethylsilylbenzophenone (species C)

2-Acetamido-4′-trimethylsilylbenzophenone (896 mg, 2.87 mmol) was dissolved in acetone (18 mL). 6 M HCl (19 mL) was added and the solution was stirred at 70 °C for 5 h. After cooling to room temperature, the solution was neutralized with a saturated aqueous solution of sodium bicarbonate and then a saturated aqueous solution of potassium carbonate (1 mL) was added. The solution was extracted with CH_2_Cl_2_ (3 × 75 mL) and the combined organic phases were dried over magnesium sulfate. After evaporation of the solvent, an orange oil was received, which was used in the next step of the synthesis without further purification. ESI-MS (acetonitrile/methanol + 1% water), positive: *m*/*z* 292.18 [M + Na]^+^ (calcd *m*/*z* for [(C_16_H_19_NOSi + Na)]^+^ 292.11).

#### 2-Chloroacetamido-4′-trimethylsilylbenzophenone (species D)

To a solution of crude 2-amino-4′-trimethylsilylbenzophenone (1.0 g, 2.87 mmol) in chloroform (10 mL) was added chloroacetyl chloride (274 μL, 3.44 mmol) in chloroform (2 mL) and the resulting solution was stirred at 80 °C for 1 h. The solution was cooled to room temperature and washed with water (2 × 30 mL). The organic phase was dried over magnesium sulfate and concentrated *in vacuo*. The orange oil was diluted with hexane (30 mL) and the solution was concentrated *in vacuo* again to obtain a yellowish solid. This step could be repeated if the solid was not obtained after the first cycle. The crude product was taken up in hexane (4 mL), filtered and washed with hexane (1 mL). The product was obtained as an off-white solid (mother liquor in the freezer increased the yield). Yield: 574 mg, 58%. ^1^H NMR (500 MHz, DMSO-*d*_*6*_), *δ*, ppm: 10.61 (s, 1H), 7.77 (d, *J* = 7.7 Hz, 1H), 7.71–7.59 (m, 5H), 7.44 (dd, *J* = 7.7, 1.4 Hz, 1H), 7.30 (td, *J* = 7.6, 1.0 Hz, 1H), 4.13 (s, 2H), 0.31–0.24 (m, 9H). ESI-MS (acetonitrile/methanol + 1% water), negative: *m*/*z* 343.95 [M − H]^−^ (calcd *m*/*z* for [(C_18_H_20_NO_2_SiCl − H^+^)]^−^ 344.09).

#### 2-Azidoacetamido-4′-trimethylsilylbenzophenone (species E)

2-Chloroacetamido-4′-trimethylsilylbenzophenone (574 mg, 1.66 mmol) was dissolved in DMF (4 mL). At 60 °C, sodium azide (124 mg, 1.91 mmol) was added and the resulting suspension was heated at 60 °C for 1 h. Subsequently, water was added dropwise (10 mL) and the cooled solution was extracted with hexane (3 × 80 mL). The residual solid, after removal of hexane, was dissolved in ethyl acetate. The aqueous phase was extracted again with hexane (80 mL). The combined organic phases were dried over magnesium sulfate and concentrated *in vacuo* to give a light beige solid. Yield: 582 mg (99%). ^1^H NMR (500 MHz, DMSO-*d*_*6*_) *δ*, ppm: 10.38 (s, 1H), 7.72 (d, *J* = 7.5 Hz, 1H), 7.70–7.60 (m, 5H), 7.44 (dd, *J* = 7.7, 1.3 Hz, 1H), 7.30 (td, *J* = 7.6, 1.1 Hz, 1H), 3.84 (s, 2H), 0.29 (d, *J* = 3.2 Hz, 9H).

#### 3-Azido-4-(4-(trimethylsilyl)phenyl)quinolin-2(1*H*)-one (species F)

2-Azidoacetamido-4′-trimethylsilylbenzophenone (582 mg, 1.64 mmol) was dissolved in EtOH (20 mL). Then 30% aqueous NaOH (2–3 drops) was added and the resulting solution was stirred at 60 °C for 5 h. The suspension was cooled to −18 °C overnight and filtered. The white solid was washed with hexane (2 mL) and dried *in vacuo*. Yield: 387 mg, 70%. ^1^H NMR (500 MHz, DMSO-*d*_*6*_), *δ*, ppm: 7.67 (d, *J* = 8.0 Hz, 2H), 7.46–7.42 (m, 1H), 7.39 (d, *J* = 7.4 Hz, 1H), 7.32 (d, *J* = 8.0 Hz, 2H), 7.12–7.08 (m, 1H), 6.99 (dd, *J* = 8.2, 1.0 Hz, 1H), 0.32–0.30 (m, 9H), NH not visible. ESI-MS (acetonitrile/methanol + 1% water), positive: *m*/*z* 691.22 [2M + Na]^+^ (calcd *m*/*z* for [(C_36_H_36_N_8_O_2_Si_2_ + Na)]^+^ 691.24).

#### 9-Trimethylsilylindolo[2,3-*c*]quinoline-2(1*H*)-one (species G)

3-Azido-4-(4-(trimethylsilyl)phenyl)quinolin-2(1*H*)-one (387 mg, 1.16 mmol) was dissolved in toluene (20 mL). The solution was stirred at 130 °C for 2 h. The white suspension was cooled to −18 °C for 3 h, filtered and washed with hexane (4 mL) to give a white solid. Yield: 281 mg, 79%. ^1^H NMR (600 MHz, DMSO-*d*_*6*_), *δ*, ppm: 12.27 (s, 1H, H^5^), 11.88 (s, 1H, H^7^), 8.46 (d, *J* = 7.8 Hz, 1H, H^11^), 8.42 (dd, *J* = 7.9, 1.1 Hz, 1H, H^1^), 7.78 (s, 1H, H^8^), 7.50 (dd, *J* = 8.1, 1.0 Hz, 1H, H^4^), 7.44 (dd, *J* = 8.0, 0.8 Hz, 1H, H^10^), 7.42–7.38 (m, 1H, H^3^), 7.36–7.32 (m, 1H, H^2^), 0.36–0.30 (m, 9H, H^12^). ^13^C NMR (151 MHz, DMSO-*d*_*6*_), *δ*, ppm: 155.77 (Cq, C^6^), 138.65 (Cq, C^7a^), 136.77 (Cq, C^9^), 134.93 (Cq, C^4a^), 127.77 (Cq, C^6a^), 125.88 (CH, C^3^), 124.86 (CH, C^10^), 122.99 (CH, C^1^), 122.68 (Cq, C^11a^), 122.31 (CH, C^2^), 121.74 (CH, C^11^), 118.04 (Cq, C^11c^), 118.03 (Cq, C^11b^), 117.86 (CH, C^8^), 116.07 (CH, C^4^), −0.90 (CH_3_, C^12^). ^29^Si NMR (119 MHz, DMSO-*d*_*6*_), *δ*, ppm: −3.44 (s). ESI-MS (acetonitrile/methanol + 1% water), positive: *m*/*z* 307.12 [M + H]^+^ (calcd *m*/*z* for [(C_18_H_18_N_2_OSi + H)]^+^ 307.12).

#### 9-Trimethylsilylindolo[2,3-*c*]quinoline-2(1*H*)-thione (species H)

To a solution of 9-trimethylsilylindolo[2,3-*c*]quinoline-2(1*H*)-one (152 mg, 0.5 mmol) in dry toluene (15 mL), Lawesson's reagent (80 mg, 0.2 mmol) was added under an argon atmosphere. The resulting solution was stirred at 110 °C overnight. After cooling to room temperature, the solvent was removed under reduced pressure and the residue taken in a mixture of diethylether and water 1 : 1 (30 mL). The organic phase was dried over magnesium sulfate and concentrated *in vacuo* to give a yellow solid. Yield: 180 mg, 99%. ^1^H NMR (500 MHz, DMSO-*d*_*6*_), *δ*, ppm: 13.72 (s, 1H), 12.05 (s, 1H), 8.63–8.51 (m, 2H), 7.96 (s, 1H), 7.89–7.83 (m, 1H), 7.53 (qd, *J* = 7.2, 5.6 Hz, 2H), 7.48 (d, *J* = 8.0 Hz, 1H), 0.34 (s, 9H).

#### 7-Hydrazinyl-9-trimethylsilylindolo[2,3-*c*]quinoline (species I)

9-Trimethylsilylindolo[2,3-*c*]quinoline-2(1*H*)-thione (136 mg, 0.42 mmol) was suspended in hydrazine monohydrate (5 mL). The resulting suspension was stirred at 135 °C overnight. After cooling to 4 °C for 4 h, the solid was filtered off, washed with diethylether (5 mL) and dried *in vacuo* at 80 °C for 2 h. The product was obtained as a light beige solid. Yield: 119 mg, 88%. ^1^H NMR (500 MHz, DMSO-*d*_*6*_), *δ*, ppm: 11.47 (s, 1H), 8.48 (d, *J* = 25.5 Hz, 2H), 8.14 (s, 1H), 7.86 (s, 1H), 7.78 (d, *J* = 7.8 Hz, 1H), 7.45 (t, *J* = 8.5 Hz, 2H), 7.39 (d, *J* = 6.8 Hz, 1H), 4.66 (s, 2H), 0.35 (s, 9H). ESI-MS (acetonitrile/methanol + 1% water), positive: *m*/*z* 321.19 [M + H]^+^ (calcd *m*/*z* for [(C_18_H_20_N_4_Si + H)]^+^ 321.15).

#### HL^TMS^

To a suspension of species I (119 mg, 0.4 mmol) in anoxic methanol (2 mL), 2-acetylpyridine (46 μL, 0.41 mmol) was added. The resulting suspension was stirred at 85 °C overnight. The yellow precipitate was filtered off, washed with methanol (1 mL) and dried at 50 °C *in vacuo* overnight to give an orange solid. Yield: 135 mg, 77%. Anal. calcd for C_25_H_25_N_5_Si·0.75H_2_O (*M*_r_ = 436.69), %: C, 68.69; H, 6.11; N, 16.02. Found, %: C, 68.50; H, 5.84; N, 15.87. ^1^H NMR (700 MHz, DMSO-*d*_*6*_), *δ*, ppm: 11.93 (s, 1H, H^7^), 10.77 (s, 1H, H^5^), 8.72 (d, *J* = 7.9 Hz, 1H, H^20^), 8.63 (d, *J* = 3.7 Hz, 1H, H^17^), 8.38 (d, *J* = 7.9 Hz, 1H, H^11^), 8.32 (d, *J* = 7.6 Hz, 1H, H^1^), 7.91–7.84 (m, 3H, H^19^, H^2^, H^8^), 7.42 (d, *J* = 7.8 Hz, 1H, H^10^), 7.41–7.38 (m, 1H, H^18^), 7.36 (t, *J* = 7.4 Hz, 1H, H^3^), 7.27 (t, *J* = 7.2 Hz, 1H, H^4^), 2.68 (s, 3H, H^21^), 0.35 (s, 9H, H^22^). ^13^C NMR (176 MHz, DMSO-*d*_*6*_), *δ*, ppm: 156,62 (Cq, C^15^), 148.45 (CH, C^17^), 144.77 (Cq, C^6^), 138.92 (Cq, C^7a^), 136.02 (Cq, C^9^), 135.90 (CH, C^19^), 134.88 (Cq, C^4a^), 127.60 (Cq, C^6a^), 125.80 (CH, C^3^), 124.87 (CH, C^10^), 123.41 (CH, C^18^), 122.95 (Cq, C^11a^), 122.83 (CH, C^1^), 121.97 (CH, C^4^), 121.31 (CH, C^11^), 121.03 (CH, C^20^), 118.82 (Cq, C^11c^), 117.70 (CH, C^8^), 116.50 (CH, C^2^), 115.31 (Cq, C^11b^), 13.13 (CH_3_, C^21^), −0.84 (CH_3_, C^22^). ESI-MS (acetonitrile/methanol + 1% water), positive: *m*/*z* 424.25 [M + H]^+^ (calcd *m*/*z* for [(C_25_H_25_N_5_Si + H)]^+^ 424.20).

#### Cu(HL^TMS^)Cl_2_ (1)

To a suspension of HL^TMS^ (60 mg, 0.14 mmol) in methanol (14 mL), a solution of CuCl_2_·2H_2_O (24 mg, 0.14 mmol) in methanol (0.2 mL) was added. The resulting suspension was stirred at 85 °C for 30 min. The precipitate was filtered off, washed with methanol (2 mL) and dried at 50 °C *in vacuo* to give an ochre solid. Yield: 80 mg, 99%. Anal. calcd for C_25_H_25_Cl_2_CuN_5_Si·2.5H_2_O (*M*_r_ = 603.07), %: C, 49.79; H, 5.01; N, 11.61. Found, %: C, 49.65; H, 4.44; N, 11.50. ESI-MS (acetonitrile/methanol + 1% water), positive: *m*/*z* 521.13 [M − Cl]^+^ (calcd *m*/*z* for [(C_25_H_25_N_5_Cl_2_CuSi − Cl)]^+^ 521.09).

#### Zn(HL^TMS^)Cl_2_ (2)

To a suspension of HL^TMS^ (60 mg, 0.14 mmol) in methanol (14 mL), a solution of ZnCl_2_·2H_2_O (24 mg, 0.14 mmol) in methanol (0.2 mL) was added. The resulting suspension was stirred at 85 °C for 30 min. The precipitate was filtered off, washed with methanol (2 mL) and dried at 50 °C *in vacuo* to give a yellow solid. Yield: 80 mg, 99%. Anal. calcd for C_25_H_25_Cl_2_N_5_SiZn·H_2_O (*M*_r_ = 575.06), %: C, 51.96; H, 4.70; N, 12.11. Found, %: C, 51.92; H, 4.42; N, 12.09. ^1^H NMR (400 MHz, DMSO-*d*_*6*_), *δ*, ppm: 12.38 (s, 1H, H^7^), 11.57 (s, 1H, H^12^), 8.88 (d, *J* = 4.7 Hz, 1H, H^1^), 8.79 (d, *J* = 4.1 Hz, 1H, H^17^), 8.74 (d, *J* = 4.9 Hz, 1H, H^4^), 7.99 (d, *J* = 8.0 Hz, 1H, H^11^), 8.26 (t, *J* = 7.4 Hz, 1H, H^19^), 8.19 (d, *J* = 7.5 Hz, 1H, H^20^), 7.98 (s, 1H, H^8^), 7.82 (t, *J* = 5.7 Hz, 1H, H^18^), 7.66–7.65 (m, 2H, H^2^, H^3^), 7.59 (d, *J* = 7.8 Hz, 1H, H^10^), 2.86 (s, 3H, H^21^), 0.39 (s, 9H, H^22^). ^13^C NMR (100 MHz, DMSO-*d*_*6*_), *δ*, ppm: 148.70 (CH, C^17^), 147.14 (Cq, C^14^), 146.96 (Cq, C^15^), 140.92 (Cq, C^6^), 140.36 (CH, C^19^), 139.44 (Cq, C^9^), 139.24 (Cq, C^7a^), 138.43 (Cq, C^4a^), 127.57 (CH, C^1^), 126.57 (CH, C^18^), 125.91 (CH, C^2^), 125.39 (CH, C^10^), 125.15 (CH, C^3^), 122.83 (2xCH, C^4^, C^20^), 122.49 (Cq, C^11c^), 122.28 (CH, C^11^), 121.74 (Cq, C^6a^), 121.04 (2xCq, C^11a^, C^11b^), 117.74 (CH, C^8^), 13.42 (CH_3_, C^21^), −0.99 (CH_3_, C^22^). ESI-MS (acetonitrile/methanol + 1% water), positive: *m*/*z* 522.12 [M−Cl]^+^ (calcd *m*/*z* for [(C_25_H_25_N_5_SiZnCl)]^+^ 522.09); negative: *m*/*z* 558.02 [M − H^+^]^−^ (calcd *m*/*z* for [(C_25_H_24_N_5_Cl_2_SiZn)]^−^ 558.04);

### Physical measurements

Elemental analysis was carried out with a Carlo–Erba microanalyzer at the Microanalytical Laboratory at the Faculty of Chemistry, University of Vienna. The samples for electrospray ionization mass spectrometry (ESI-MS) were measured using an amaZon speed ETD Bruker instrument. The expected and experimental isotope distributions were compared. 1D (^1^H, ^13^C) and 2D (^1^H-^1^H COSY, ^1^H-^13^C HSQC, ^1^H-^13^C HMBC) NMR spectra were acquired on a Bruker AV NEO 500 or AV III 600 spectrometer in DMSO-*d*_*6*_ at 25 °C. The NMR spectra for Zn(ii) complex 2 were recorded on a Bruker Avance NEO spectrometer operating at 400 MHz, with a 5 mm probe for direct detection of ^1^H, ^13^C, ^19^F, and ^29^Si. DMSO-*d*_*6*_ was used as the solvent and the spectra were calibrated on the residual peak of the solvent (*δ* 2.50 ppm for ^1^H and 39.50 ppm for ^13^C). The spectra were recorded at room temperature using the standard parameter sets provided by Bruker.

### Crystallographic structure determination

SC-XRD data for 1 were collected with a Stadivari Diffractometer (STOE & Cie GmbH, Germany) equipped with an EIGER2 R500 detector (Dectris Ltd, Switzerland). Data were processed and scaled with the STOE software suite X-Area (STOE & Cie GmbH). Crystal data, data collection parameters, and structure refinement details are given in Table S2.† The structure was solved by direct methods and refined by full-matrix least-squares techniques. Non-H atoms were refined with anisotropic displacement parameters. H atoms were inserted in calculated positions and refined with a riding model. The following computer programs and hardware were used: structure solution, SHELXT^39^ and refinement, SHELXL;^40^ molecular diagrams, ORTEP;^41^ computer, Intel CoreDuo. CCDC 2378782 (1).†

### Sample preparation and data collection for complex 2 by ED

Crystals were provided as a suspension in MeCN. The vial was sonicated for 10 min in order to generate a suspension of small, evenly sized crystals. A droplet of the resulting suspension was placed onto a TEM grid featuring a lacey carbon film (copper, Ted Pella, 200-mesh). The evaporation of acetonitrile was observed with a light microscope. The grid was then transferred to an ELSA698 tomography holder (Gatan INC) at room temperature and inserted into a JEM2100Plus TEM (200 keV LaB_6_, JEOL). The TEM was equipped with a 1024 × 1024 pixel SINGLA detector, featuring a 320 μm thick silicon sensor and a pixel size of 75 μm. Diffraction patterns were recorded by irradiating crystalline grains of ∼1–2 μm in length with the electron beam with a rotation from ∼−70° to 70°, *i.e.* the maximum possible rotation range of the goniometer of the JEM2100Plus. Data were processed with XDS.^42^ Five datasets measured with different crystal grains were merged to improve the completeness. The detector distance for each dataset was refined during the CORRECT step of the XDS program to improve the accuracy of the unit cell parameters. The structures were solved by employing SHELXT^39^ for *ab initio* phasing, followed by refinement and model building using SHELXL and ShelXle, respectively.^40,43^ Scattering factors were fitted in Cromer–Mann parametrisation *f*(*s*) = sum (*i* = 1…4) *a*_*i* exp(−*b*_*i* * *s*^2^) + *c* against the values tabulated in Table 4.2.6.8 of the International Tables of Crystallography, Volume C,^44^ starting from the parameters published by Peng 1999.^45^ Hydrogen atoms were placed in riding positions at inter-nuclear distances.^46^ CCDC 2378723 (2).†

### Spectrophotometric titrations and solubility tests

The UV-vis spectra for the proligand and complexes were recorded using an Agilent Cary 3500 8-path, fibre optic photometer equipped with a Xe-flash lamp light source. Additionally, an Agilent Cary 8454 diode array spectrophotometer was also applied for photostability assay. The path length was 1 cm. Stock solutions were prepared in DMSO (VWR International, Hungary) in *ca.* 5 mM concentration. Samples for spectroscopic measurement were diluted with aqueous HCl (pH 2.0); NaOH solution (pH 11.7 ) or 10 mM solution of 4-(2-hydroxyethyl)piperazine-1-ethanesulfonic acid (HEPES) (pH 7.4). The final DMSO content was ≤1% (v/v) in the samples.

Fluorescence measurements were implemented with a Fluoromax Plus fluorimeter (Horiba, Jobin Yvon). Since the aqueous solution of the ligand (and the Zn complex) showed photodegradation under the usual fluorometric conditions (excitation slit: 3–5 nm), we have therefore taken the following precautions: (i) every sample was scanned in the fluorimeter only once; for a new recording, a new portion was poured into the cuvette; (ii) a narrow circular window (diameter: 1 mm) and (iii) a neutral density filter (*A* = 1) were placed between the light source and the cuvette; and (iv) concomitantly the emission slit was opened wider (7–12 nm) in order to obtain reasonable spectra. This way, the loss of intensity was less than 15% during repeated measurements. Solid samples (fine crystalline compound) were measured in the solid sample holder, suitable for front-face luminescence assays, at 120° angle to the light source. The respective long pass filters (370, 400, 450 or 500 nm) were applied on the emission side in order to reduce light scattering.

### Cell-free ROS generation and redox activity

EPR measurements were performed using an X-band Bruker EMX spectrometer (Germany). The spin trapping agent DMPO (Sigma-Aldrich) was distilled before use (70 Pa; 80–90 °C). Standard settings during EPR spin trapping experiments were microwave frequency ∼9.448 GHz; power of the microwave radiation 2 mW; sweep width 200 G; centre field 3350 G; modulation amplitude 2 G; time constant 20.5 ms; and sweep time 42 s, and a series of 10 spectra with 3 scans for each measurement. Optical spectra in dimethyl sulfoxide (DMSO, SeccoSolv max. 0.025% H_2_O, Merck) were collected on a Shimadzu 3600 UV-vis-NIR spectrometer (Japan). Experiments with sodium ascorbate and spectroelectrochemical studies were performed on a UV-vis-NIR Avantes spectrometer (Model AvaSpec-2048_14-USB2). The cyclic voltammograms (CV) of the investigated samples were recorded by using 0.1 M tetrabutylammonium hexafluorophosphate (*n*Bu_4_NPF_6_, puriss quality from Fluka) as a supporting electrolyte and a single compartment electrochemical cell with a working Pt-disk electrode (from Ionode, Australia), a Pt wire as a counter electrode and an Ag wire as a pseudoreference electrode at a scan rate of 100 mV s^−1^. All potentials in voltammetric studies were quoted *vs.* ferricenium/ferrocene (Fc^+^/Fc) redox couple (purchased from Sigma-Aldrich). A Heka PG310USB (Lambrecht, Germany) potentiostat with the PotMaster 2.73 software package served as the potential control in voltammetric studies.

### Photoinduced processes monitored by EPR spin trapping

The paramagnetic intermediates generated upon UV-light exposure of the studied samples were obtained at room temperature (295 K) under air employing an EPR spectrometer EMX*Plus* (Bruker, Germany) operating at 100 kHz field modulation using the high sensitivity probe-head with a small quartz flat cell (WG 808-Q, Wilmad-LabGlass, optical cell length 0.045 cm). Standard settings during EPR spin trapping experiments were: microwave frequency ∼9.43 GHz; power of the microwave radiation 10.7 mW; magnetic field sweep width 10.0 mT; centre field 336.0 mT; modulation amplitude 0.1 mT; time constant 5.12 ms; sweep time 30 s, and number of scans NS = 10. The solutions of the studied samples, prepared in DMSO (anhydrous, ≥99% H_2_O, Merck) or mixed solvent with ultra-pure water, containing the spin trap DMPO were mixed directly before the EPR measurements, carefully aerated by a gentle air stream, and immediately transferred to the flat cell. The samples were irradiated directly in the EPR resonator (High Sensitivity Probe-head, Bruker), and the EPR spectra were recorded *in situ*. The *g*-values were determined using the built-in magnetometer. The irradiation source was a UV LED monochromatic radiator (LED@365 nm, Hönle, Germany). The value of the UVA irradiance (LED@365 nm), determined using a UVX radiometer (UVP, USA) within the EPR cavity, was 18 mW cm^−2^. All EPR experiments were performed at least in duplicate. EPR spectra acquisition started 2 min after experimental system mixing. The experimental EPR spectra were analyzed using WinEPR software (Bruker), while the calculations of spin-Hamiltonian parameters and relative concentrations of individual spin-adducts were performed with the EasySpin toolbox working on the MatLab® platform.^47^

### NCI-60 screening

The NCI-60 SRB assay was performed as described elsewhere.^48^ The GI_50_ values (the concentration of the metal complex causing 50% growth inhibition), TGI value (the concentration of the complex causing 0% cell growth), and LC_50_ (the concentration of the complex causing 50% cell death) were interpolated from dose–response curves that were plots of percentage cell growth *vs.* concentration of compounds assayed.

### Cell culture

The biological effects of the metal-free Schiff base HL^TMS^, and Cu(ii) and Zn(ii) complexes 1 and 2 were tested on two human adenocarcinoma cell lines, A549 lung and MCF-7 breast carcinoma cells. Both cell lines were obtained from ATCC and maintained in RPMI 1640 cell culture media (Biosera, Cholet, France) complemented with 10% fetal bovine serum (FBS, Biosera, Cholet, France), 2 mM l-glutamine (Biosera, Cholet, France), 0.01% streptomycin and 0.005% penicillin (Biosera, Cholet, France). They were cultured under standard conditions in a 37 °C incubator under 5% CO_2_ and 95% humidity. In addition, non-cancerous mouse fibroblast cells (NIH/3T3) were also used and maintained under similar conditions to the cancer cells.

### MTT assay

To test the effect of HL^TMS^, and complexes 1 and 2 on the viability of A549 and MCF-7 tumor cells and the NIH/3T3 non-cancerous cells, the MTT assay was performed. For this, 1 × 10^4^ cells per well were seeded into 96-well plates and left to grow. On the following day, the cells were exposed to increasing concentrations (0; 1.56; 3.125; 6.25; 12.5, and 25 μM) of compounds for 24 h. After the treatments, cells were washed with PBS and then incubated with 0.5 mg mL^−1^ MTT reagent (Sigma-Aldrich, St Louis, Missouri, USA) at 37 °C for 1 h. After incubation, 100–100 μL of DMSO (Molar Chemicals, Halásztelek, Hungary) was added to the wells, and the absorbance of the samples was measured at 570 nm using a Synergy HTX plate reader (BioTek, Winooski, Vermont, USA). The viability measurements were repeated three times using 3 independent biological replicates.

### Analysis of mitochondrial membrane potential by JC-1 staining

To test whether the treatments cause mitochondrial damage and changes in mitochondrial membrane potential, JC-1 staining was performed. For the assay, 1 × 10^5^ cells per well were seeded onto glass coverslips (VWR International, Radnor, Pennsylvania, USA) placed in 24-well plates. On the following day, cells were treated with 5 μM metal-free ligand HL^TMS^ or metal complexes 1 or 2 for 24 h. After the treatments, the cells were washed twice in PBS, and JC-1 dye (Thermo Fisher Scientific, Waltham, Massachusetts, USA) was added in 10 μg mL^−1^ final concentration diluted in cell culture media. After 15 min of incubation, the cells were washed with PBS three times, then the fluorescence of the samples was detected using an OLYMPUS BX51 microscope with an Olympus DP70 camera, and the red and green fluorescence intensity was quantified using ImageJ software. These experiments were also repeated three times with 3 biological replicates.

## Author contributions

Christopher Wittmann – data curation; formal analysis; investigation; and methodology. Iuliana Besleaga – data curation; formal analysis; and methodology. Soheil Mahmoudi – software; data curation; and investigation. Oleg Palamarciuc − investigation. Mihaela Balan-Porcarasu − formal analysis and investigation. Mihaela Dascalu − formal analysis and data curation. Sergiu Shova − visualization and investigation. Maria Cazacu − formal analysis and writing – original draft. Mónika Kiricsi − investigation and writing – original draft. Nóra Igaz − investigation. Orsolya Dömötör − investigation and writing – original draft. Éva A. Enyedy − writing – original draft; writing – review and editing; funding acquisition and resources. Dana Dvoranová − investigation and writing – original draft. Peter Rapta – investigation; methodology and writing – original draft. Vladimir B. Arion – conceptualization; funding acquisition; investigation; project administration and writing – review and editing.

## Data availability

The data supporting this article have been included as part of the ESI.†

Crystallographic data for compounds 1 and 2 have been deposited at the CCDC under accession numbers 2378782 and 2378723.

## Conflicts of interest

There are no conflicts to declare.

## Supplementary Material

DT-054-D5DT00314H-s001

DT-054-D5DT00314H-s002
