# Supplementary material for: Physical properties and cytotoxicity of Cu(ii) and Zn(ii) complexes with a TMS-substituted indolo[2,3-c]quinoline-derived Schiff base
Source: Dalton Trans. 2025 Apr 2;54(19):7882–98. doi: 10.1039/d5dt00314h (PMC12022746; doi:10.1039/d5dt00314h)
Supplement: DT-054-D5DT00314H-s001 [file DT-054-D5DT00314H-s001.pdf]

Supplementary Information (SI) for Dalton Transactions.
This journal is © The Royal Society of Chemistry 2025

Electronic Supplementary Information

for

Physical properties and cytotoxicity of the Cu(II) and Zn(II) complexes with TMS-substituted indolo[2,3-*c*]quinoline-derived Schiff base

Christopher Wittmann,[a] Iuliana Besleaga,[a] Soheil Mahmoudi,[a,b] Oleg Palamarciuc,[c,d] Mihaela Balan-Porcarasu,[e] Mihaela Dascalu,[c] Sergiu Shova,[c] Maria Cazacu,[c] Mónika Kiricsi,[f] Nóra Igaz,[f] Orsolya Dömötör,[g] Eva A. Enyedy,[g,*] Dana Dvoranová,[h] Peter Rapta,[h,*] Vladimir B. Arion[a,c,*]

[a]*University of Vienna, Institute of Inorganic Chemistry, Währinger Strasse 42, 1090 Vienna, Austria*

[b]*University of Vienna, Vienna Doctoral School in Chemistry (DoSChem), Währinger Strasse 42, 1090 Vienna, Austria*

[c]*Inorganic Polymers Department, "Petru Poni" Institute of Macromolecular Chemistry, Aleea Gr. Ghica Voda 41 A, Iasi 700487, Romania*

[d]*Physics of Semiconductors and Devices Laboratory, Faculty of Physics and Engineering and Institute of Applied Physics, Moldova State University, MD-2009 Chișinău, Republic of Moldova*

[e]*NMR Laboratory, "Petru Poni" Institute of Macromolecular Chemistry, Aleea Gr. Ghica Voda 41 A, Iasi 700487, Romania*

[f]*Department of Biochemistry and Molecular Biology, University of Szeged, Közép fasor 52, H-6726 Szeged, Hungary*

[g]*Department of Molecular and Analytical Chemistry, Interdisciplinary Excellence Centre, University of Szeged, Dóm tér 7-8, H-6720 Szeged, Hungary*

[h]*Institute of Physical Chemistry and Chemical Physics, Faculty of Chemical and Food Technology, Slovak University of Technology in Bratislava, SK-81237 Bratislava, Slovakia*

Table of Contents

NMR Numbering Scheme S3
NMR data for organic compounds S4
ESI-MS for organic compounds S14
NMR spectra of Zn(II) complex 2 .... S21
ESI mass spectra of complexes 1 and 2 .... S23
Crystallographic data S27
Stability in solution S28
NCI-60 One-Dose Screening S32
NCI-60 Five-Dose Screening S35

NMR Numbering Scheme

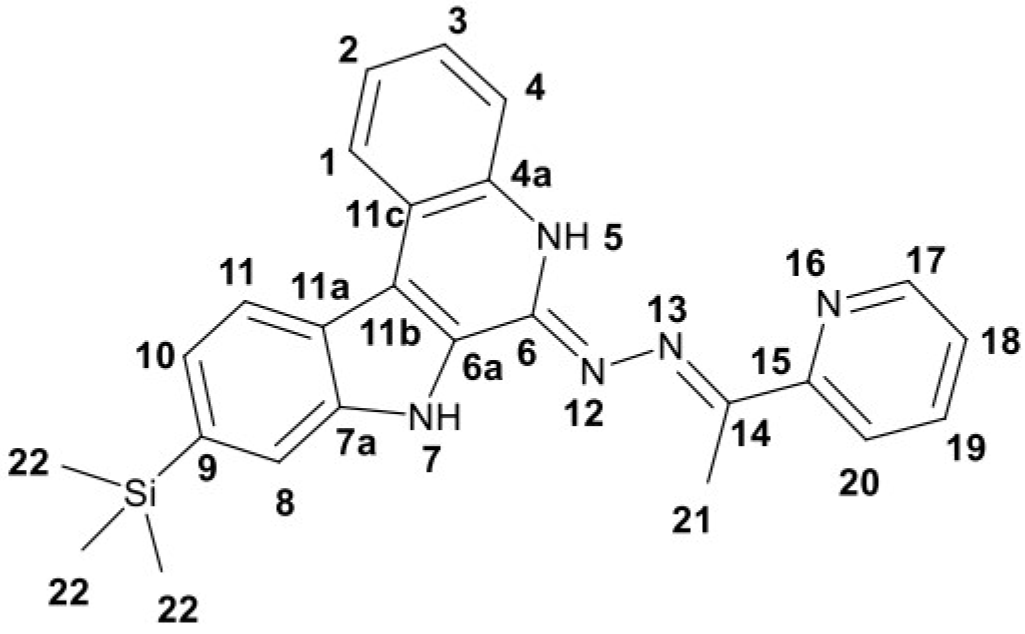

Figure S1. NMR atom numbering scheme for $\mathbf{HL^{TMS}}$.

NMR data for organic compounds

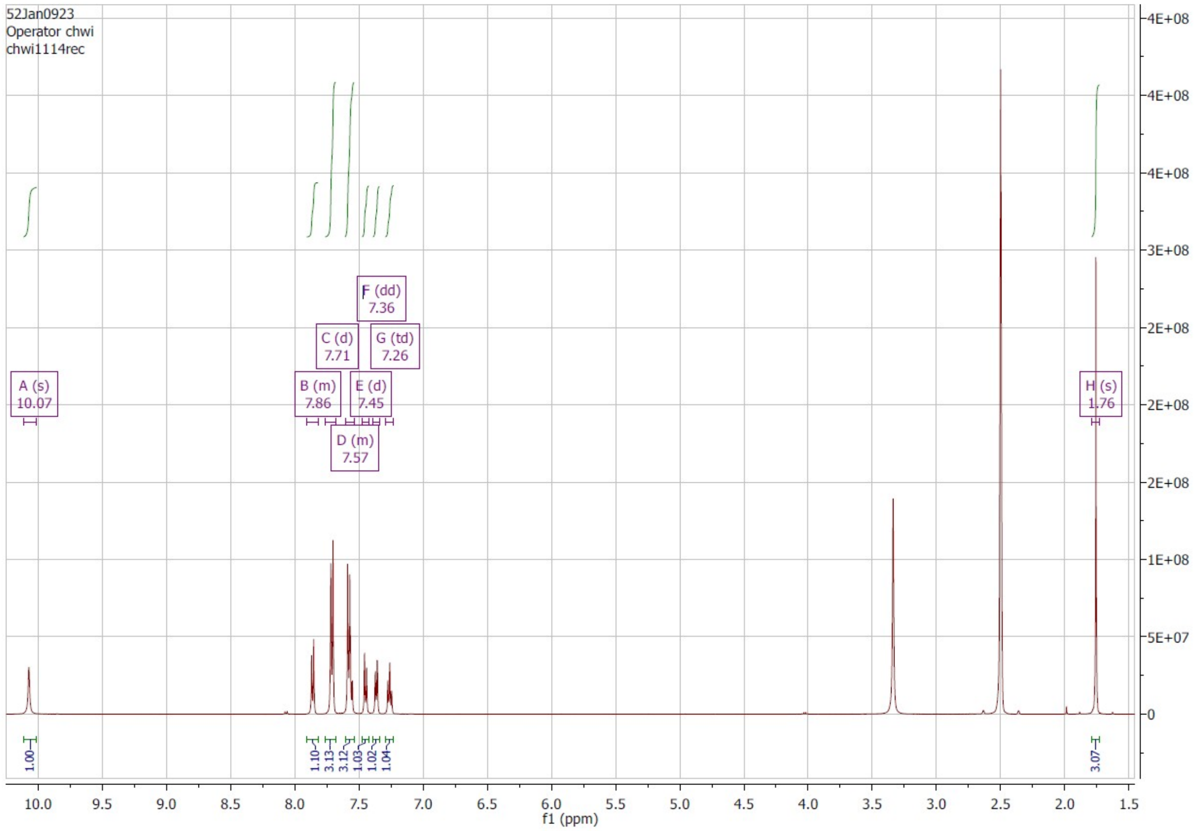

Figure S2. ^{1}H NMR spectrum of species **A** in DMSO-d_6.

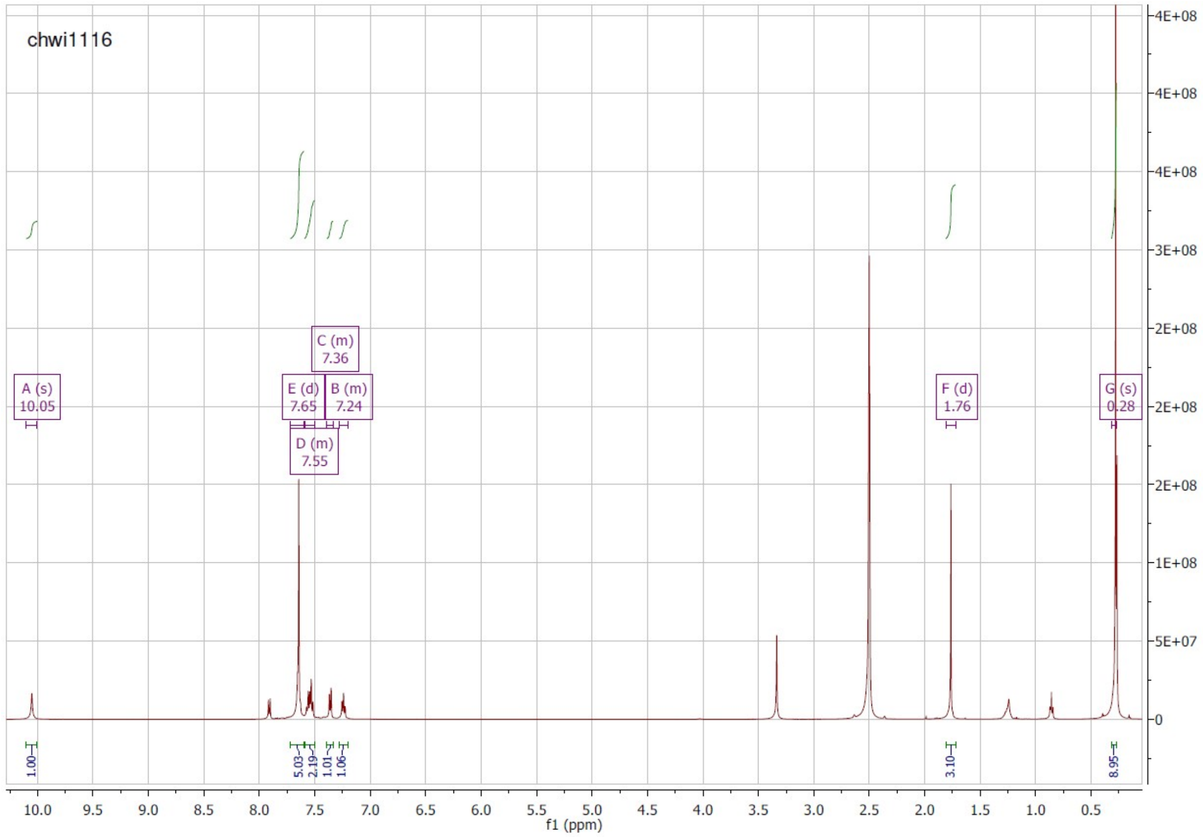

Figure S3. ^{1}H NMR spectrum of species **B** in DMSO-d_6.

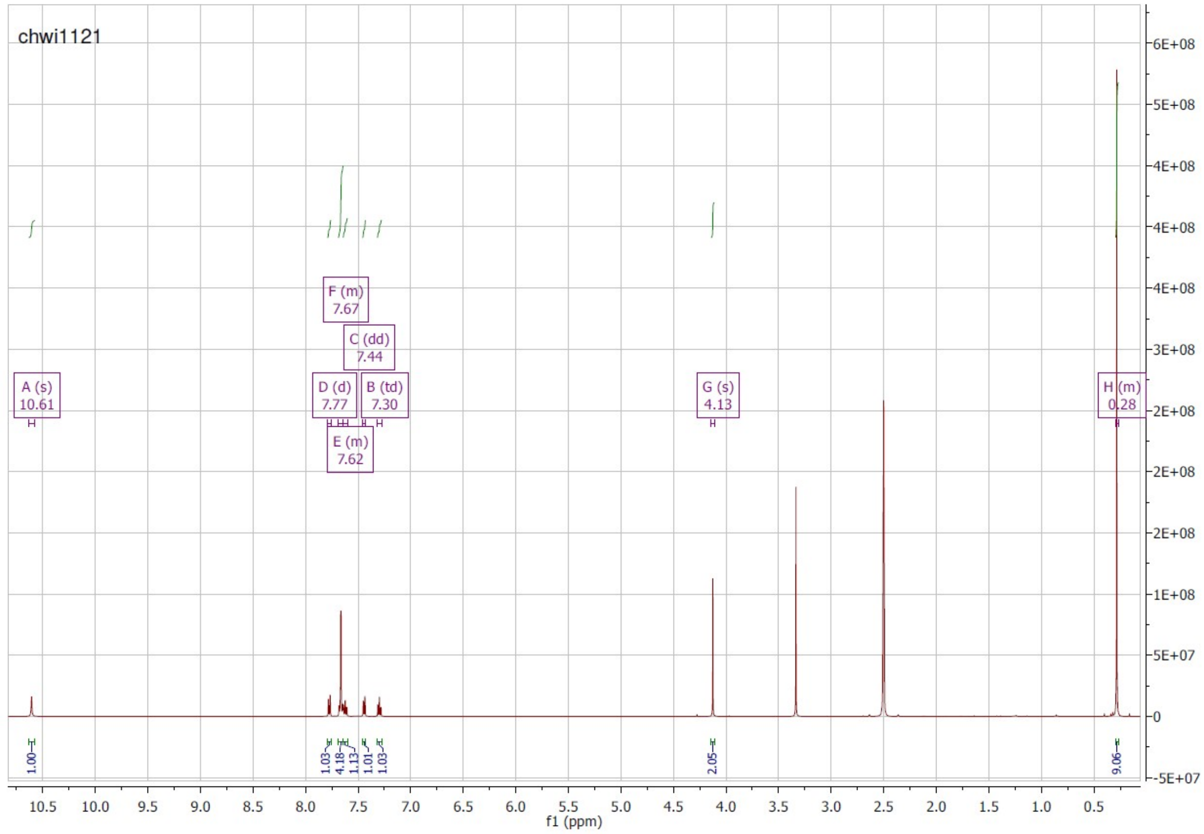

Figure S4. ^{1}H NMR spectrum of species **D** in DMSO-d_6.

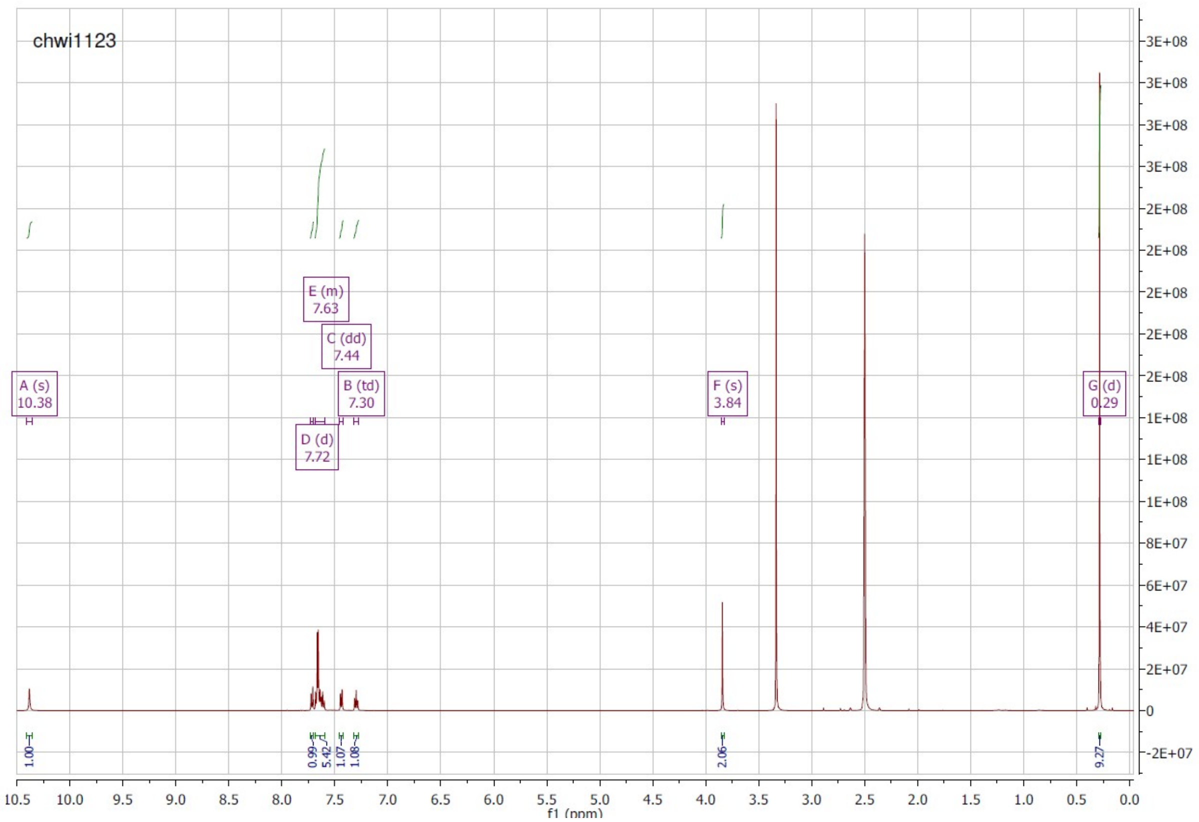

Figure S5. ^{1}H NMR spectrum of species **E** in DMSO-d_6.

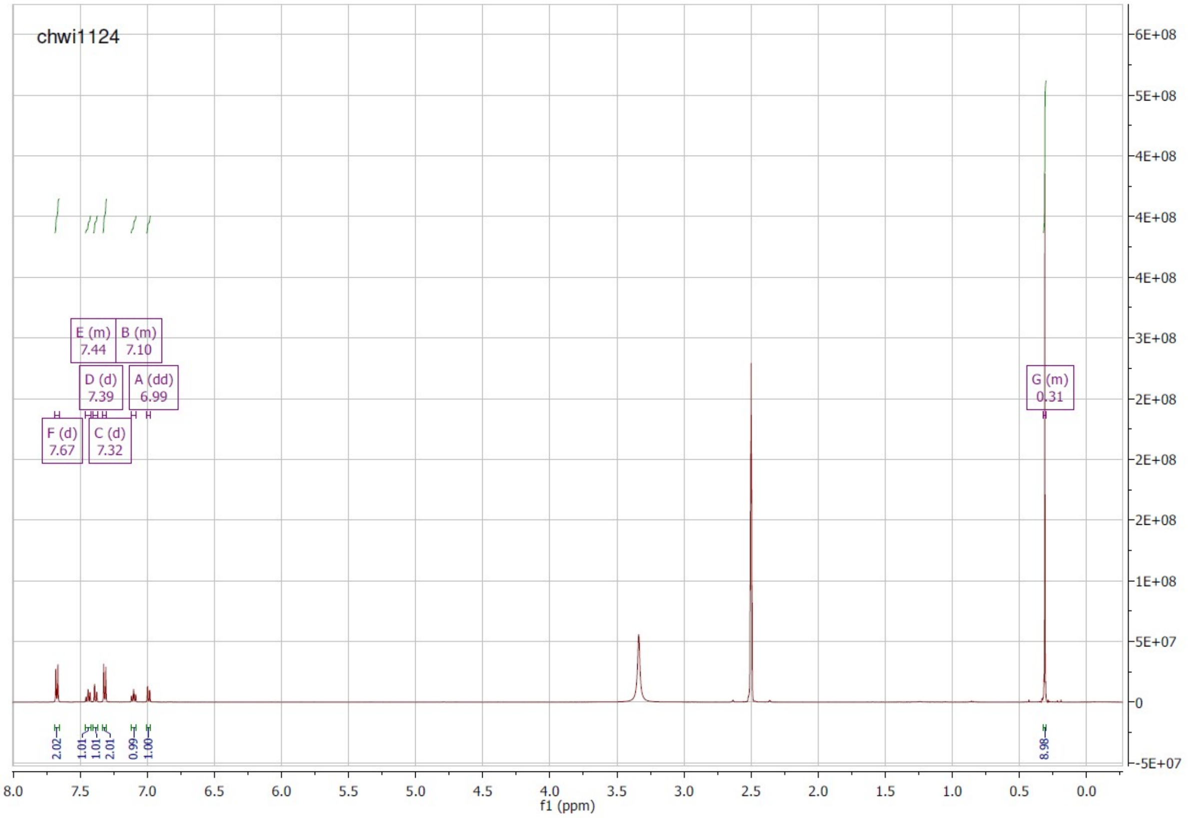

Figure S6. ^{1}H NMR spectrum of species **F** in DMSO-d_6.

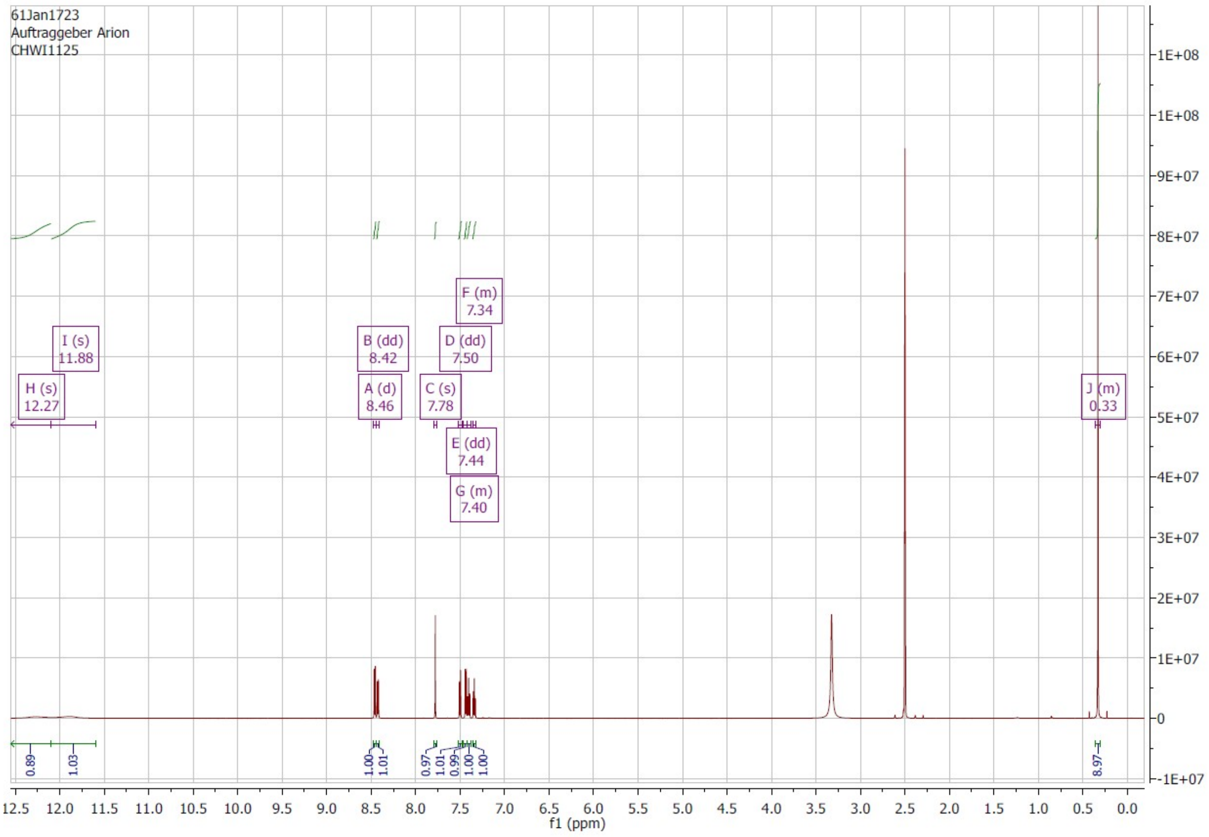

Figure S7. ^{1}H NMR spectrum of species **G** in DMSO-d_6.

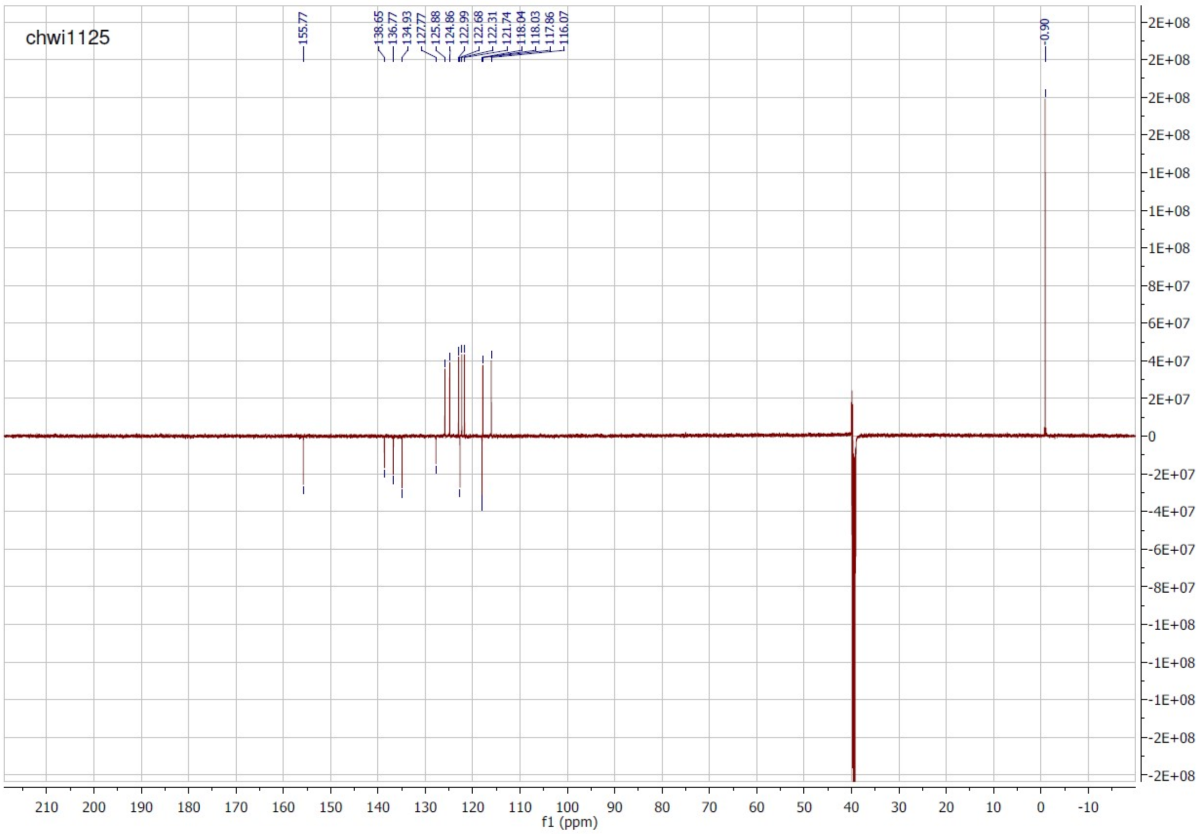

Figure S8. ^{13}C NMR spectrum of species **G** in DMSO-d_6.

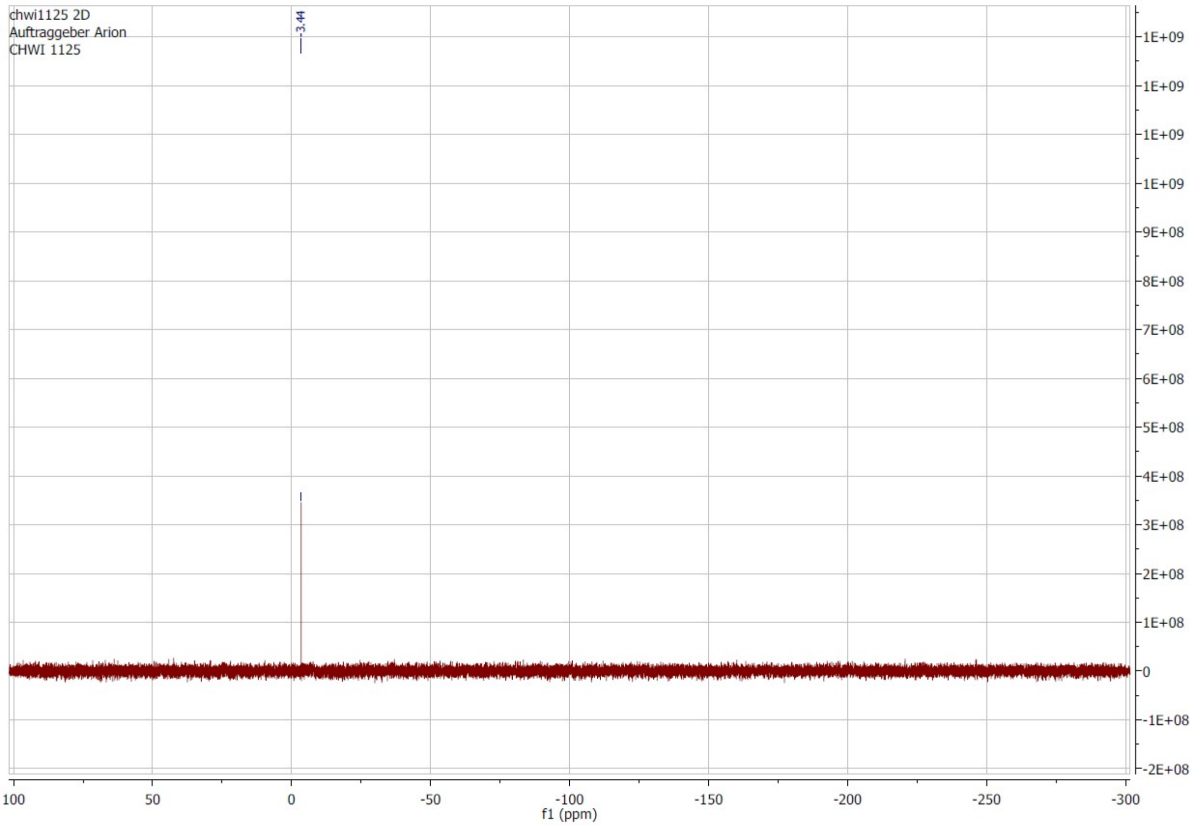

Figure S9. ^{29}Si NMR spectrum of species **G** in DMSO-d_6.

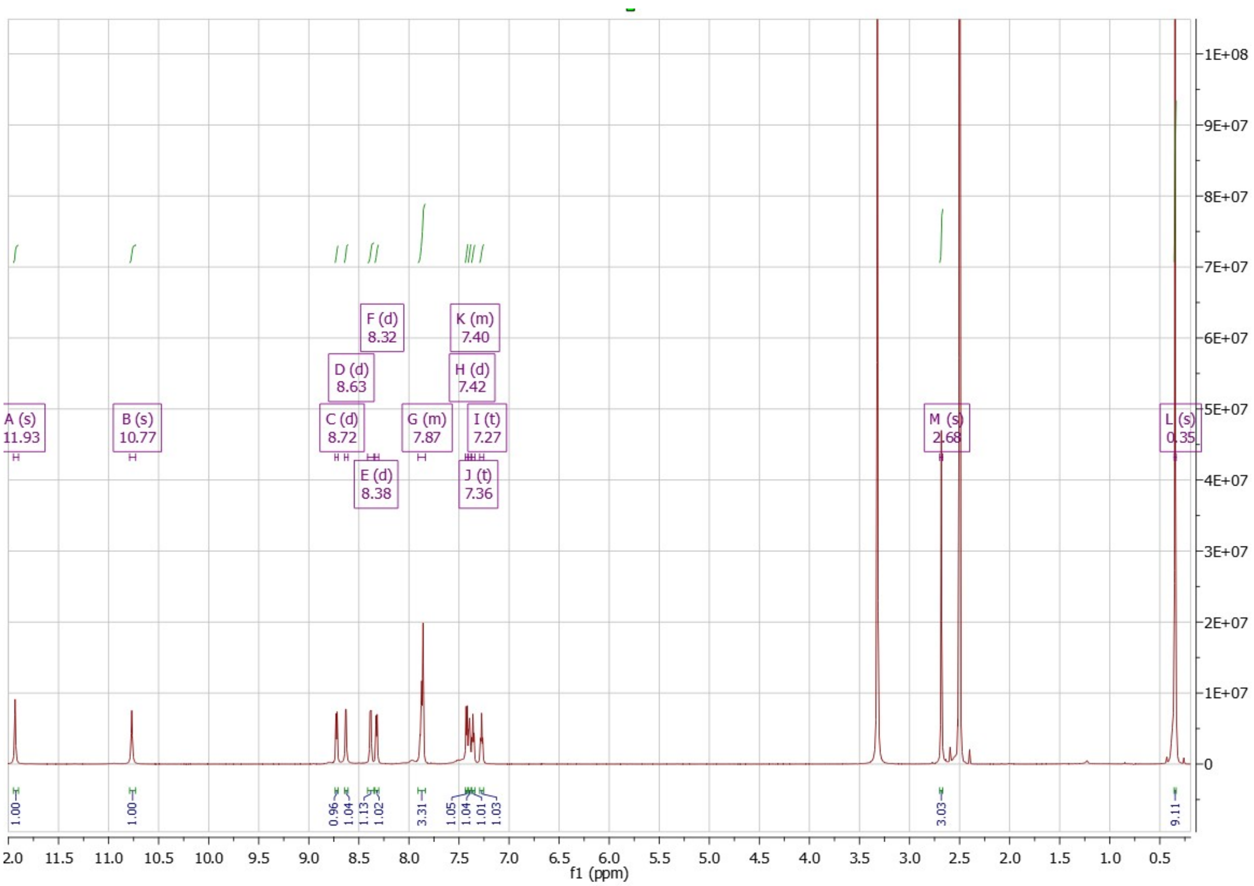

Figure S10. ^{1}H NMR spectrum of **HL^{TMS}** in DMSO-d_6.

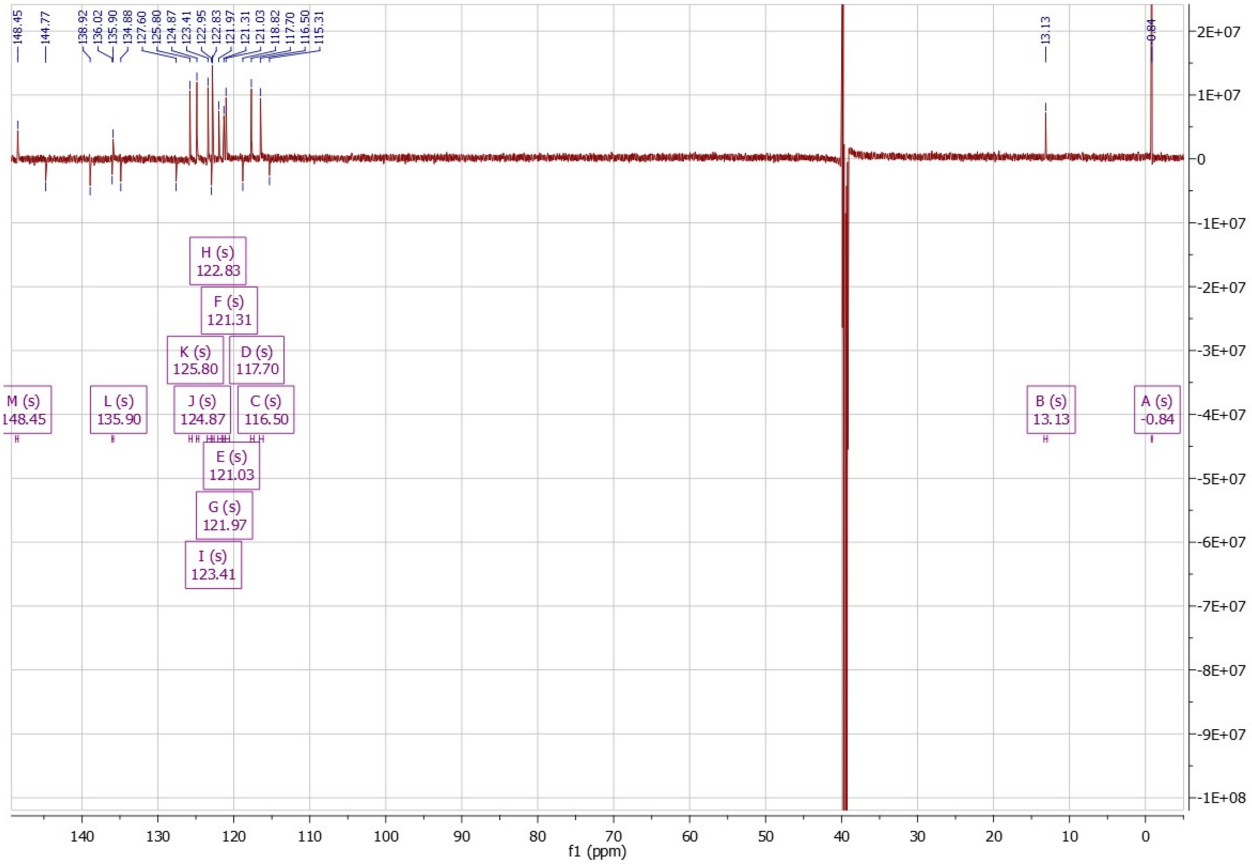

Figure S11. ^{13}C NMR spectrum of **HL^{TMS}** in DMSO-d_6.

ESI-MS for organic compounds

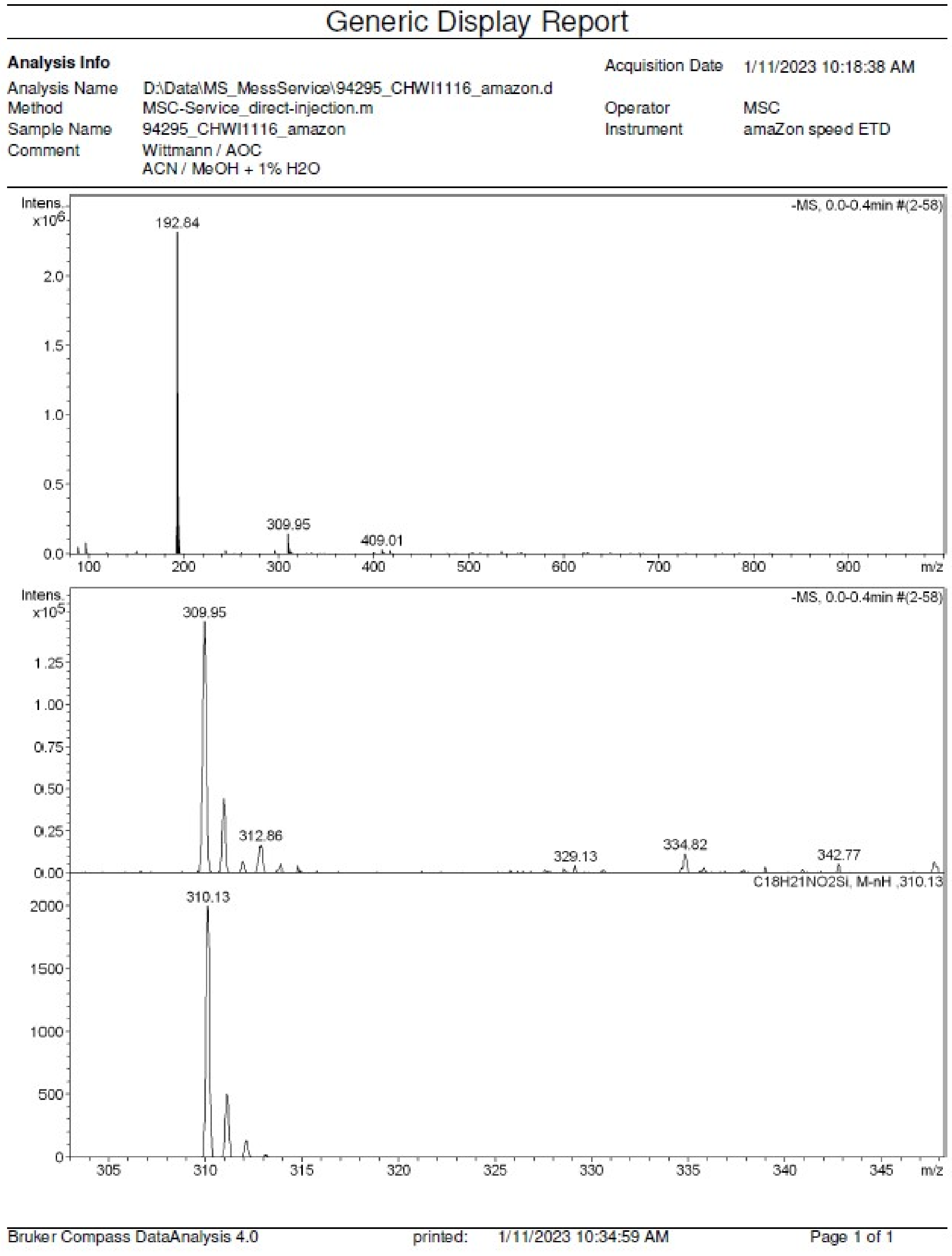

Figure S12. ESI(+) mass spectrum of **B**.

Generic Display Report

Analysis Info

Analysis Name	D:\Data\MS_MessService\94396_CHWI 1120_amazon.d	Acquisition Date	1/12/2023 2:46:45 PM
Method	MSC-Service_direct-injection.m	Operator	MSC
Sample Name	94396_CHWI 1120_amazon	Instrument	amaZon speed ETD
Comment	Wittmann/ AOC<br>ACN / MeOH + 1% H2O		

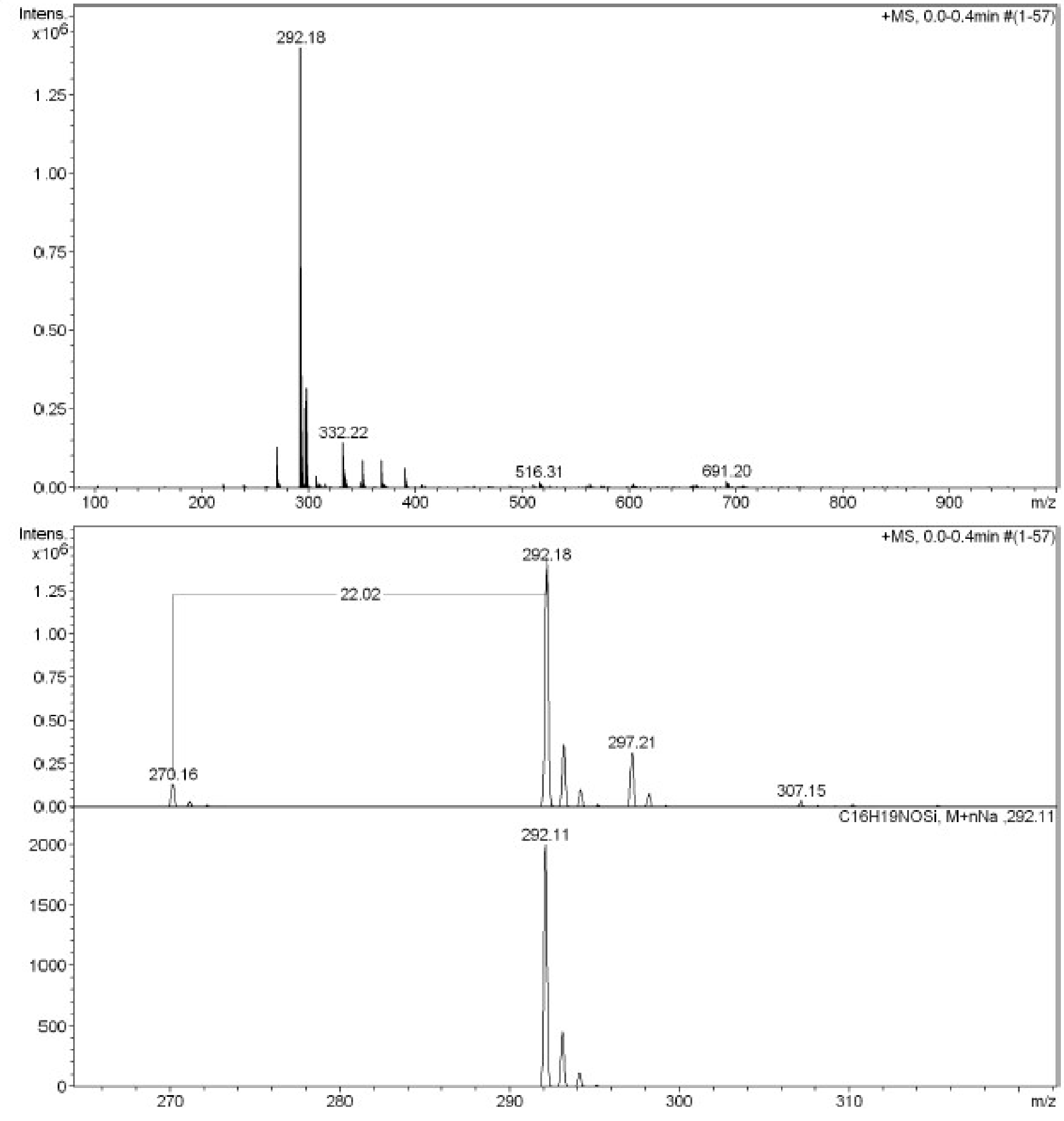

Bruker Compass DataAnalysis 4.0 printed: 1/12/2023 2:50:22 PM Page 1 of 1

Figure S13. ESI(+) mass spectrum of **C**.

Generic Display Report

**Analysis Info**		Acquisition Date	1/13/2023 11:11:30 AM
Analysis Name	D:\Data\MS_MessService\94424_CHWI1121_amazon.d		
Method	MSC-Service_direct-injection.m	Operator	MSC
Sample Name	94424_CHWI1121_amazon	Instrument	amaZon speed ETD
Comment	Wittmann / Anorg.Chem.<br>ACN / MeOH + 1% H2O		

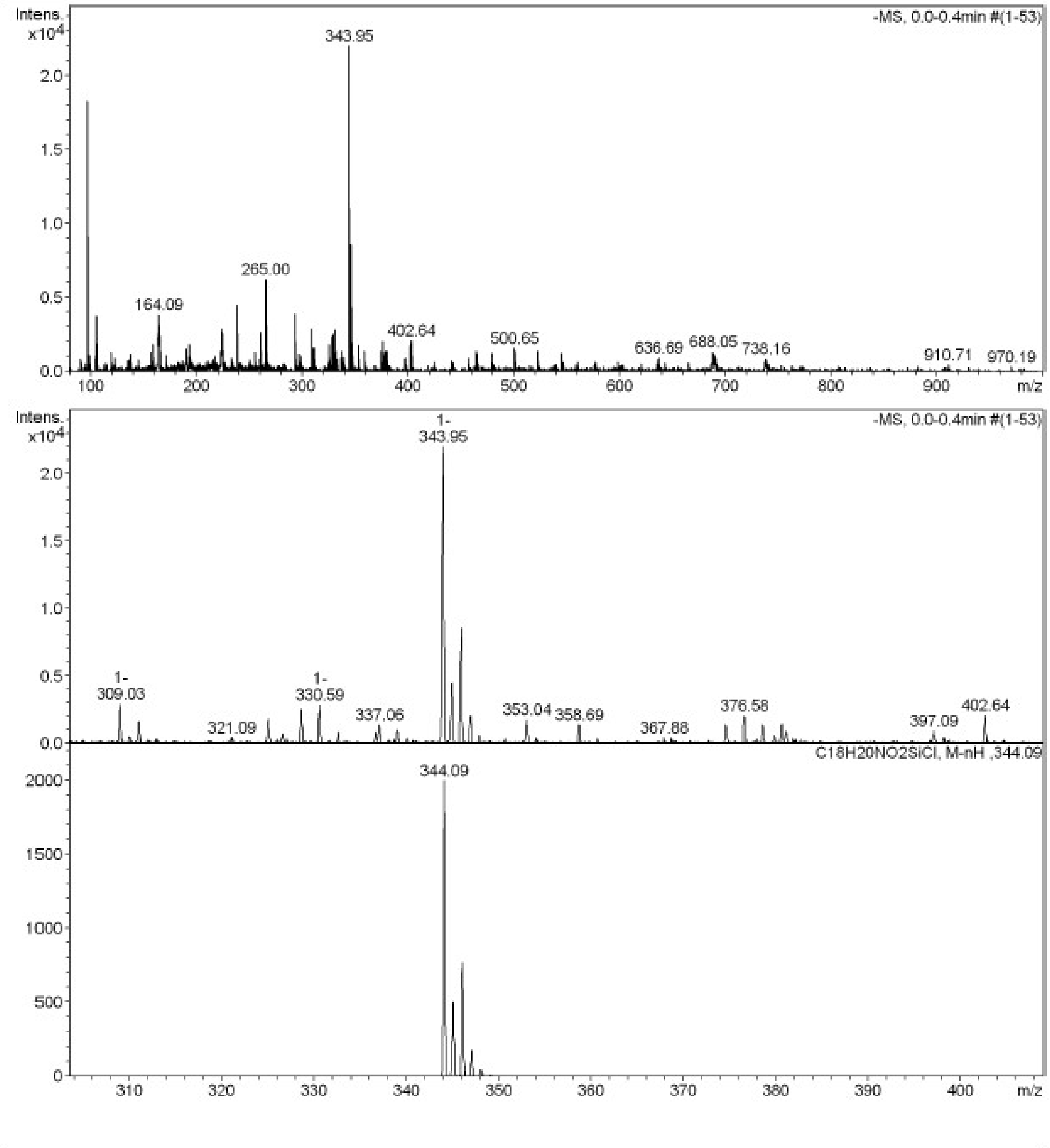

Bruker Compass DataAnalysis 4.0 printed: 1/13/2023 11:15:10 AM Page 1 of 1

Figure S14. ESI(+) mass spectrum of **D**.

Generic Display Report

Analysis Info

Analysis Name	D:\Data\MS_MessService\94472_CHWI1124_amazon.d	Acquisition Date	1/17/2023 11:34:31 AM
Method	MSC-Service_direct-injection.m	Operator	MSC
Sample Name	94472_CHWI1124_amazon	Instrument	amaZon speed ETD
Comment	Wittmann / Anorg.Chem.<br>ACN / MeOH + 1% H2O		

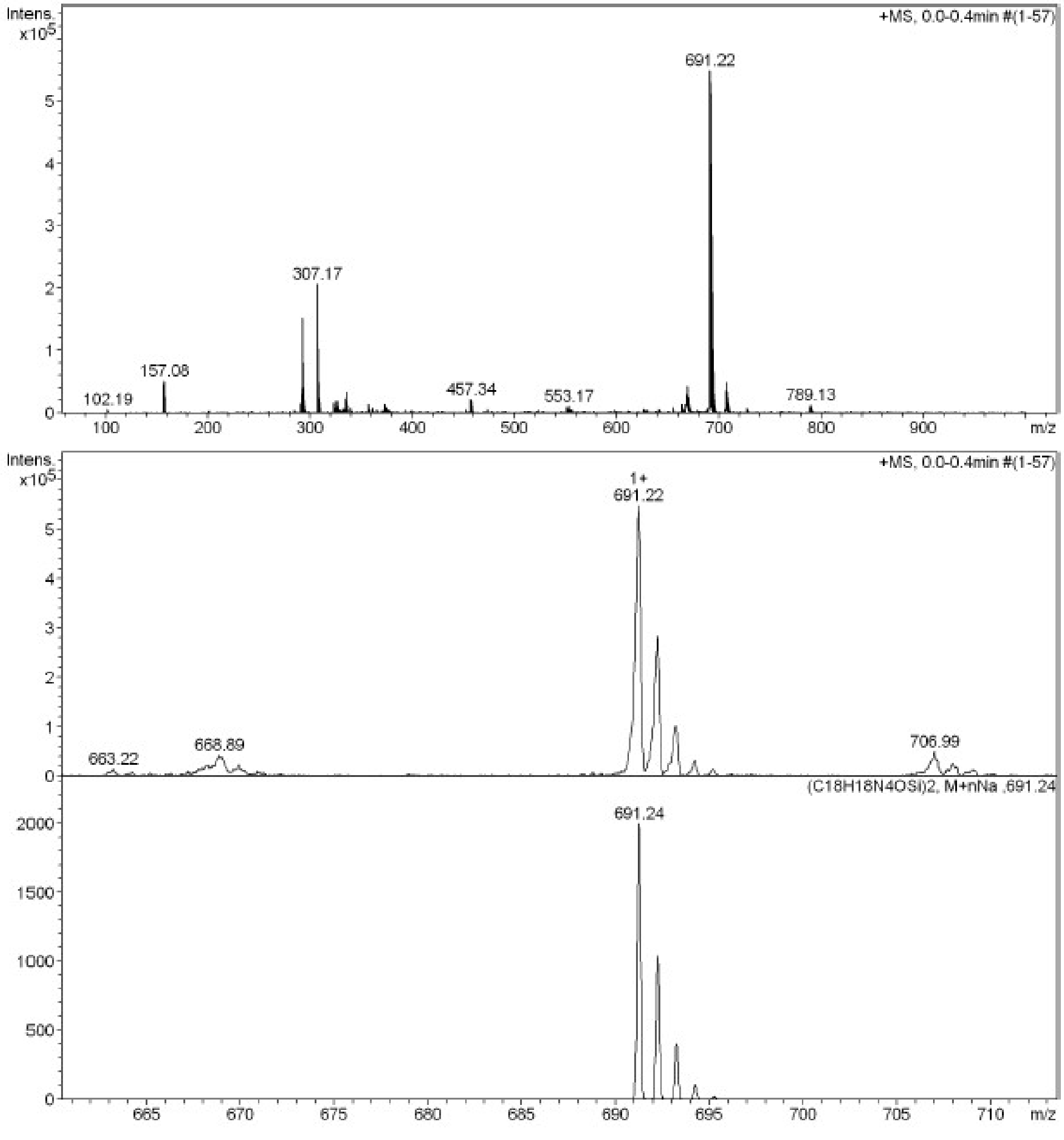

Bruker Compass DataAnalysis 4.0 printed: 1/17/2023 11:40:08 AM Page 1 of 1

Figure S15. ESI(+) mass spectrum of **F**.

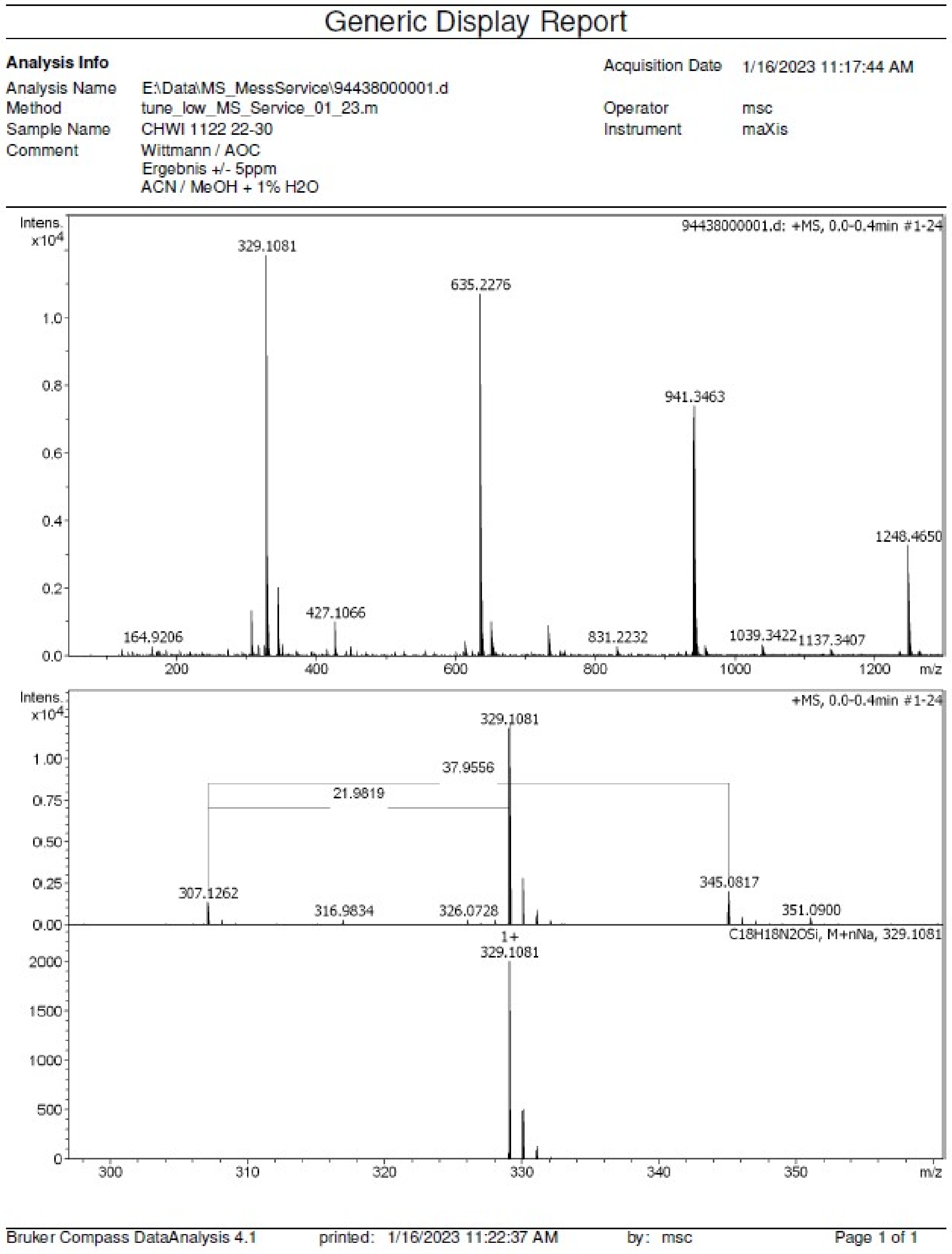

Figure S16. ESI(+) mass spectrum of **G**.

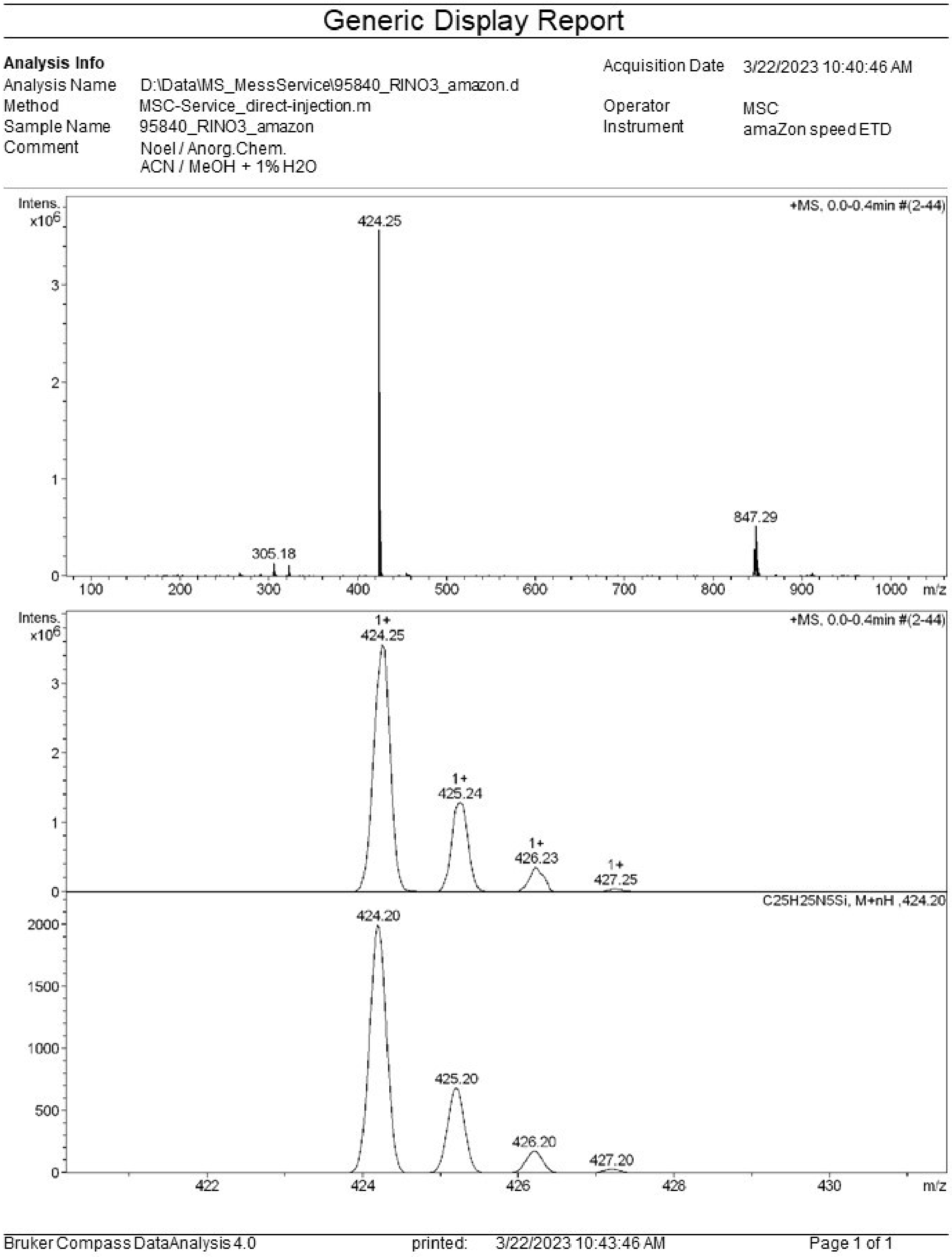

Figure S17. ESI(+) mass spectrum of $\mathbf{HL^{TMS}}$.

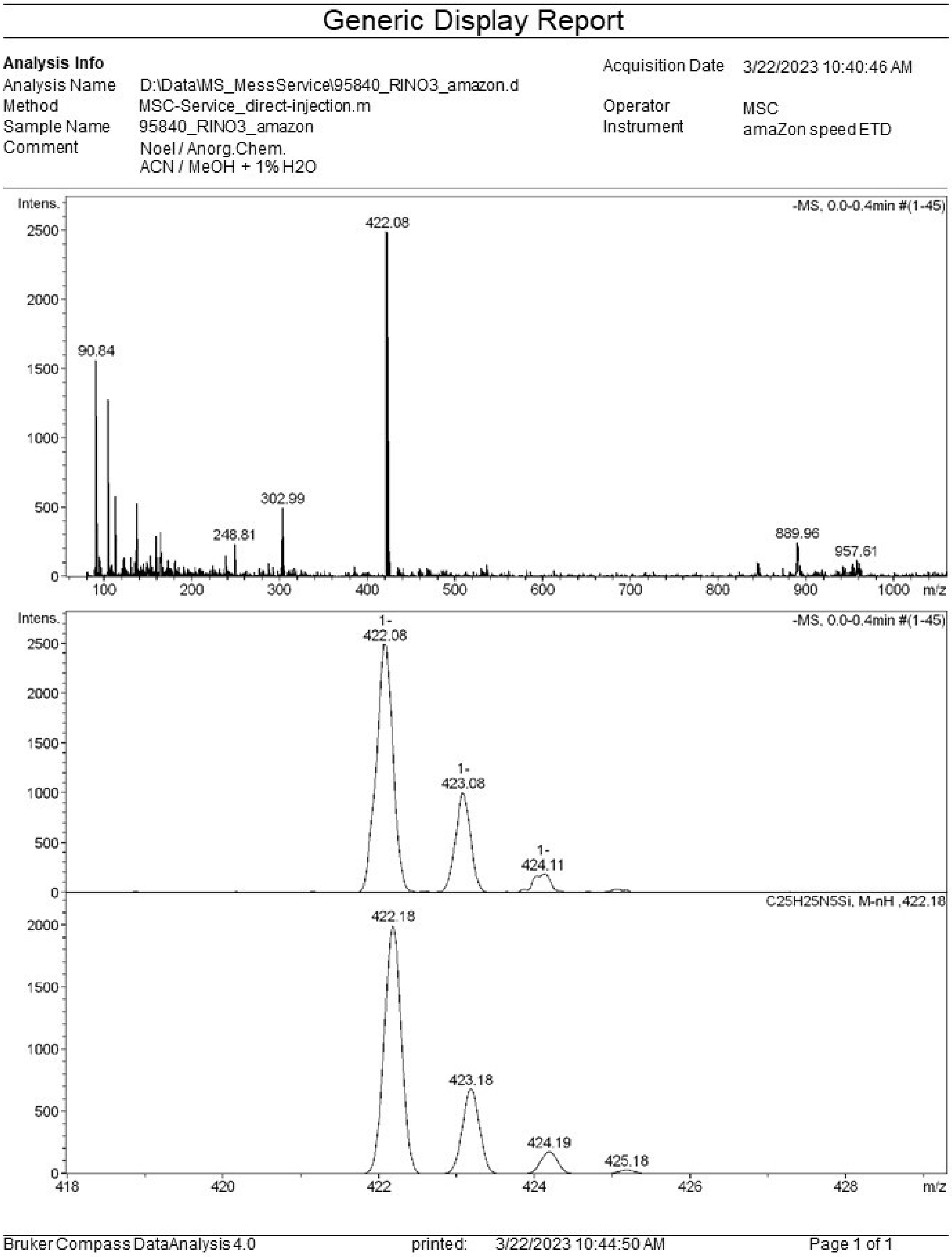

Figure S18. ESI(−) mass spectrum of $\mathbf{HL^{TMS}}$.

NMR spectra of Zn(II) complex 2

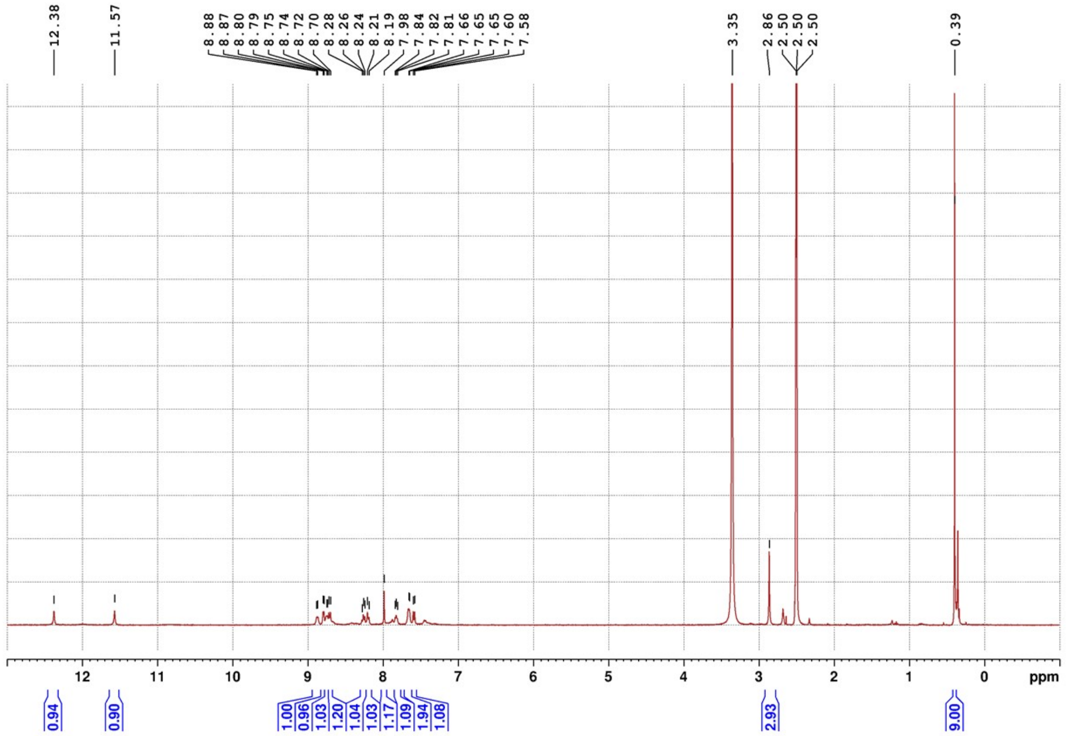

Figure S19. ^{1}H NMR spectrum of **2** in DMSO-d_6.

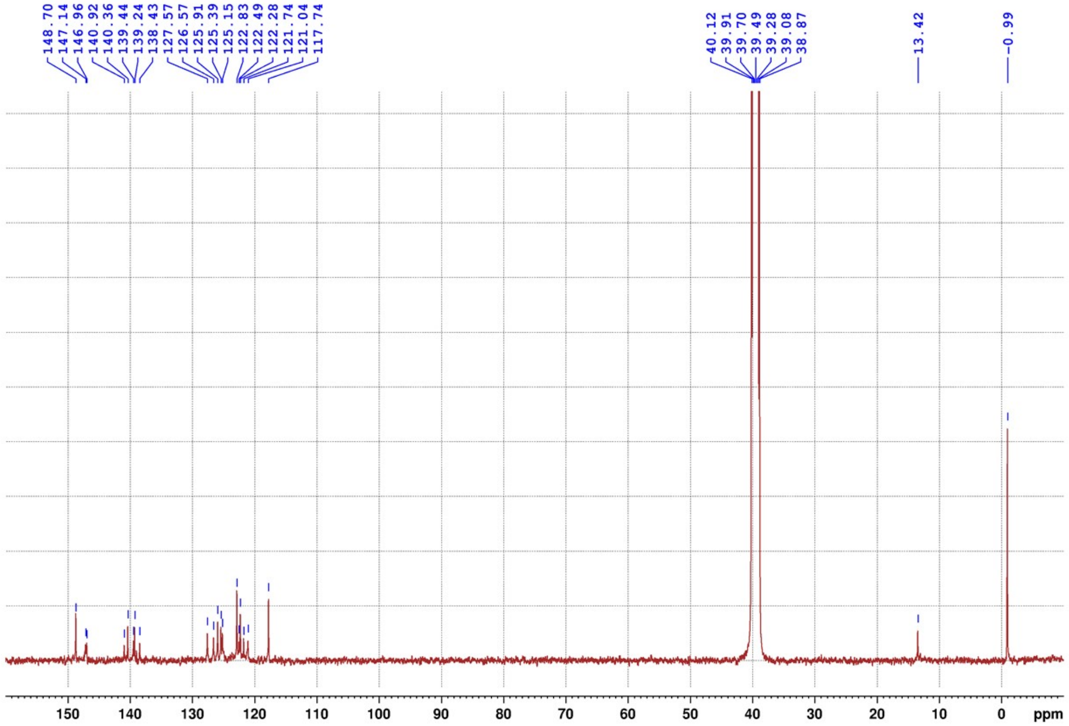

Figure S20. ^{13}C NMR spectrum of **2** in DMSO-d_6.

ESI mass spectra of complexes 1 and 2

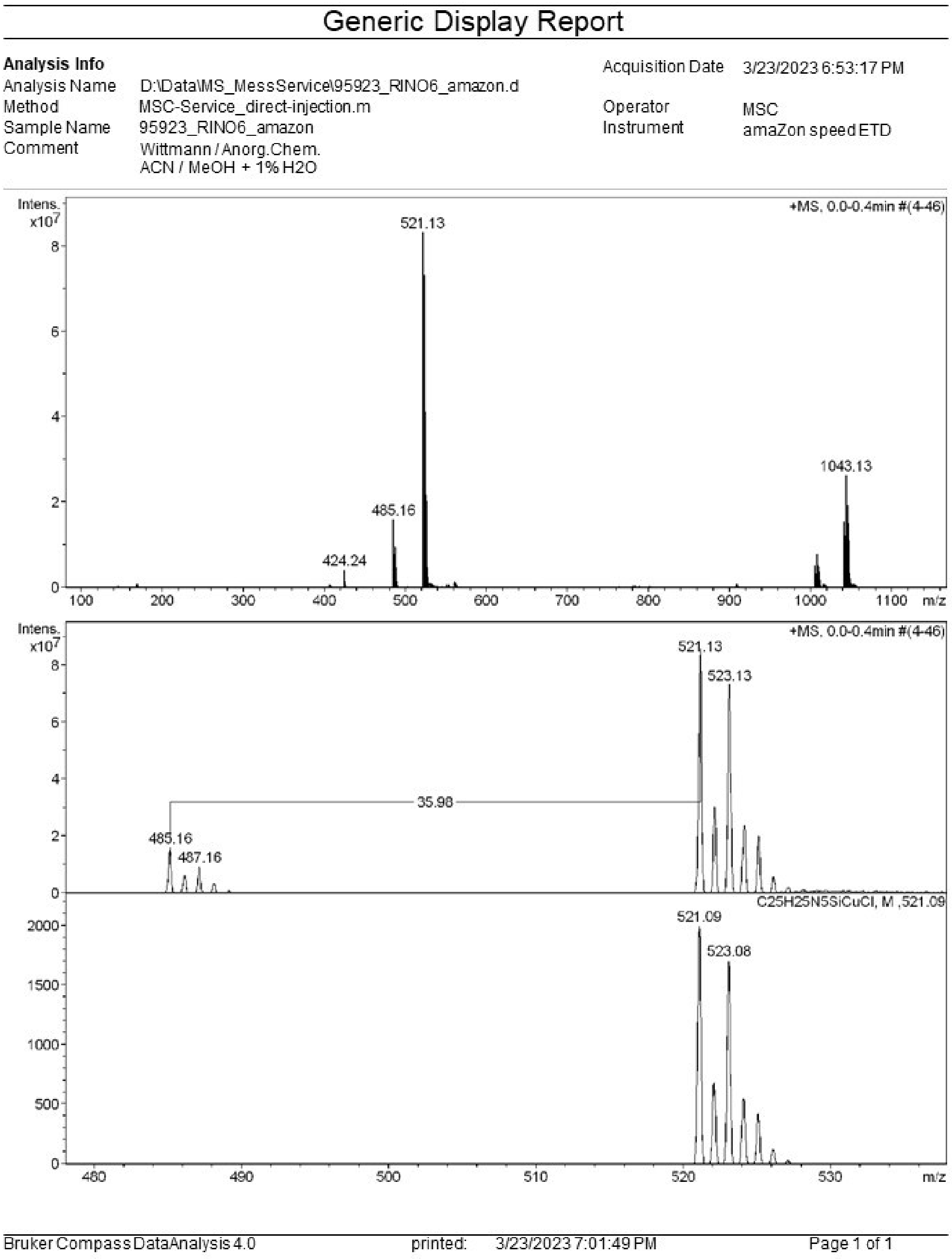

Figure S21. ESI(+) mass spectrum of **1**.

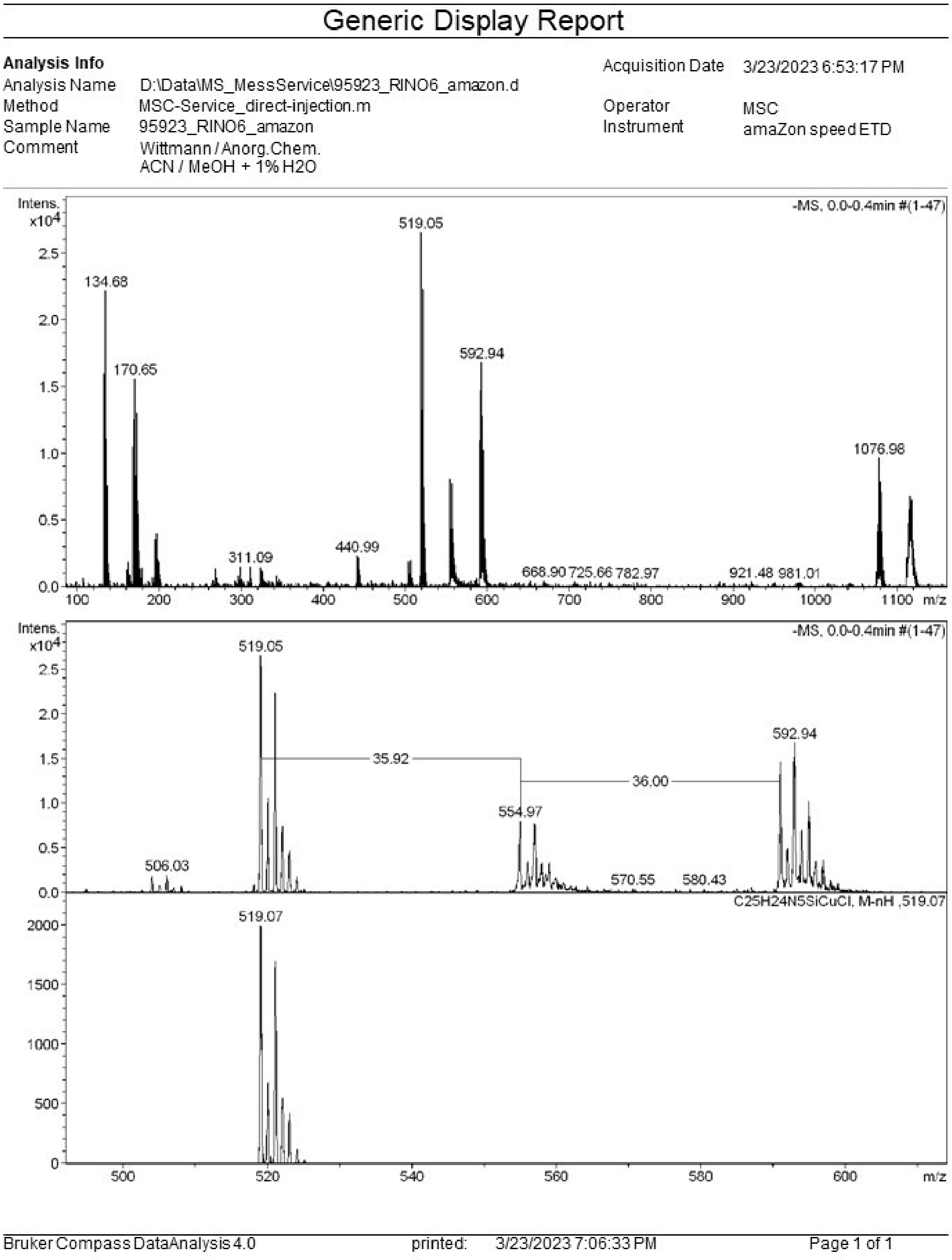

Figure S22. ESI(−) mass spectrum of **1**.

Generic Display Report

**Analysis Info**		Acquisition Date	4/26/2023 5:54:13 PM
Analysis Name	D:\Data\MS_MessService\96569_CHWI1157_amazon.d		
Method	MSC-Service_direct-injection.m	Operator	MSC
Sample Name	96569_CHWI1157_amazon	Instrument	amaZon speed ETD
Comment	Wittmann / Anorg.Chem.<br>ACN / MeOH + 1% H2O		

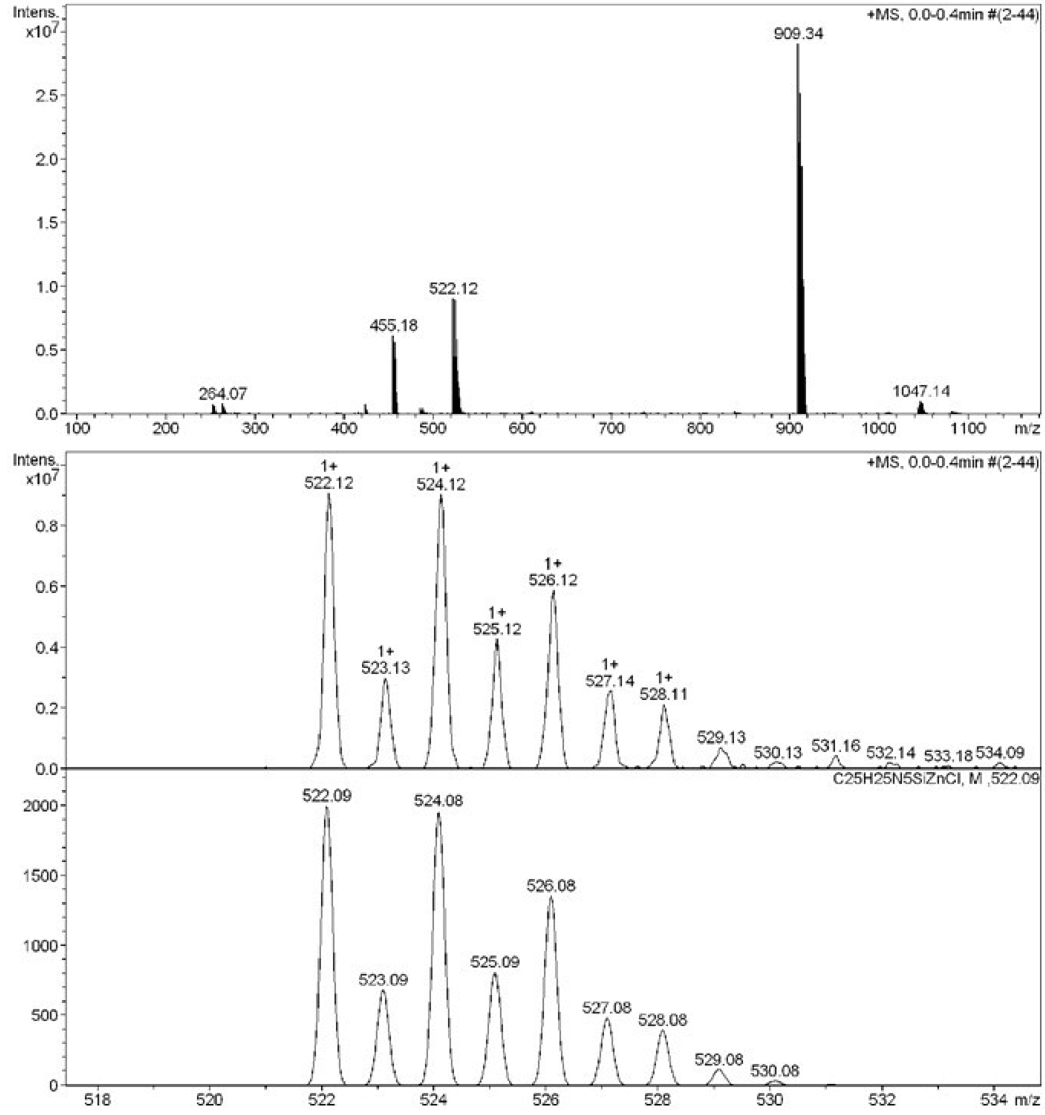

Bruker Compass DataAnalysis 4.0 printed: 4/26/2023 6:00:47 PM Page 1 of 1

Figure S23. ESI(+) mass spectrum of **2**.

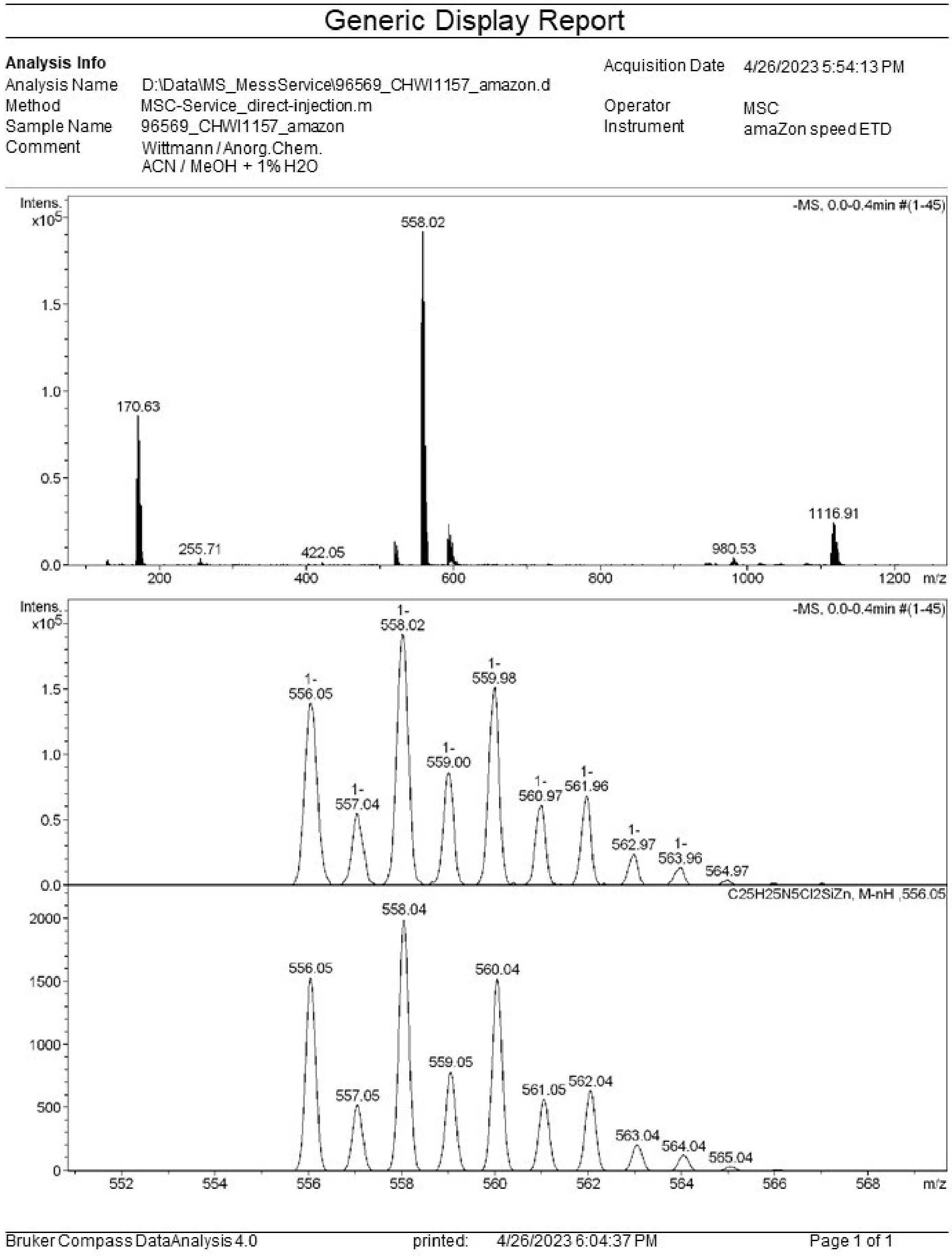

Figure S24. ESI(−) mass spectrum of **2**.

Crystallographic data

Table S1. Crystallographic data and refinement details for complexes studied.

Compound	**1**	**2**
empirical formula	$C_{25}H_{25}Cl_2CuN_5Si$	$C_{25}H_{25}Cl_2N_5SiZn$
fw	558.03	559.86
space group	monoclinic, $P2_1/c$	triclinic, $P\bar{1}$
a, Å	13.1421(7)	7.2600(14)
b, Å	14.3009(7)	12.210(2)
c, Å	14.7259(9)	15.930(3)
α,°		72.75(3)
β, °	94.735(5)	81.99(3)
γ, °		85.37(3)
V [Å^3]	2758.2(3)	1334.3(5)
Z	4	2
λ [Å]	0.71073	0.02508
ρ_{calcd}, g cm^{-3}	1.344	1.393
cryst size, mm^3	0.10 × 0.07 × 0.02	0.005 × 0.0013 × 0.0005
T [K]	100(2)	294(2)
μ, mm^{-1}	1.051	
$R_1{}^a$	0.0854	0.2068
$wR_2{}^b$	0.2770	0.4689
GOF^c	0.894	1.382
CCDC no.	2378782	2378723

^{a} $R_1 = \Sigma||F_o| - |F_c||/\Sigma|F_o|$. ^{b} $wR_2 = \{\Sigma[w(F_o{}^2 - F_c{}^2)^2]/\Sigma[w(F_o{}^2)^2]\}^{1/2}$. ^{c} GOF = $\{\Sigma[w(F_o{}^2 - F_c{}^2)^2]/(n - p)\}^{1/2}$, where n is the number of reflections and p is the total number of parameters refined.

Stability in solution

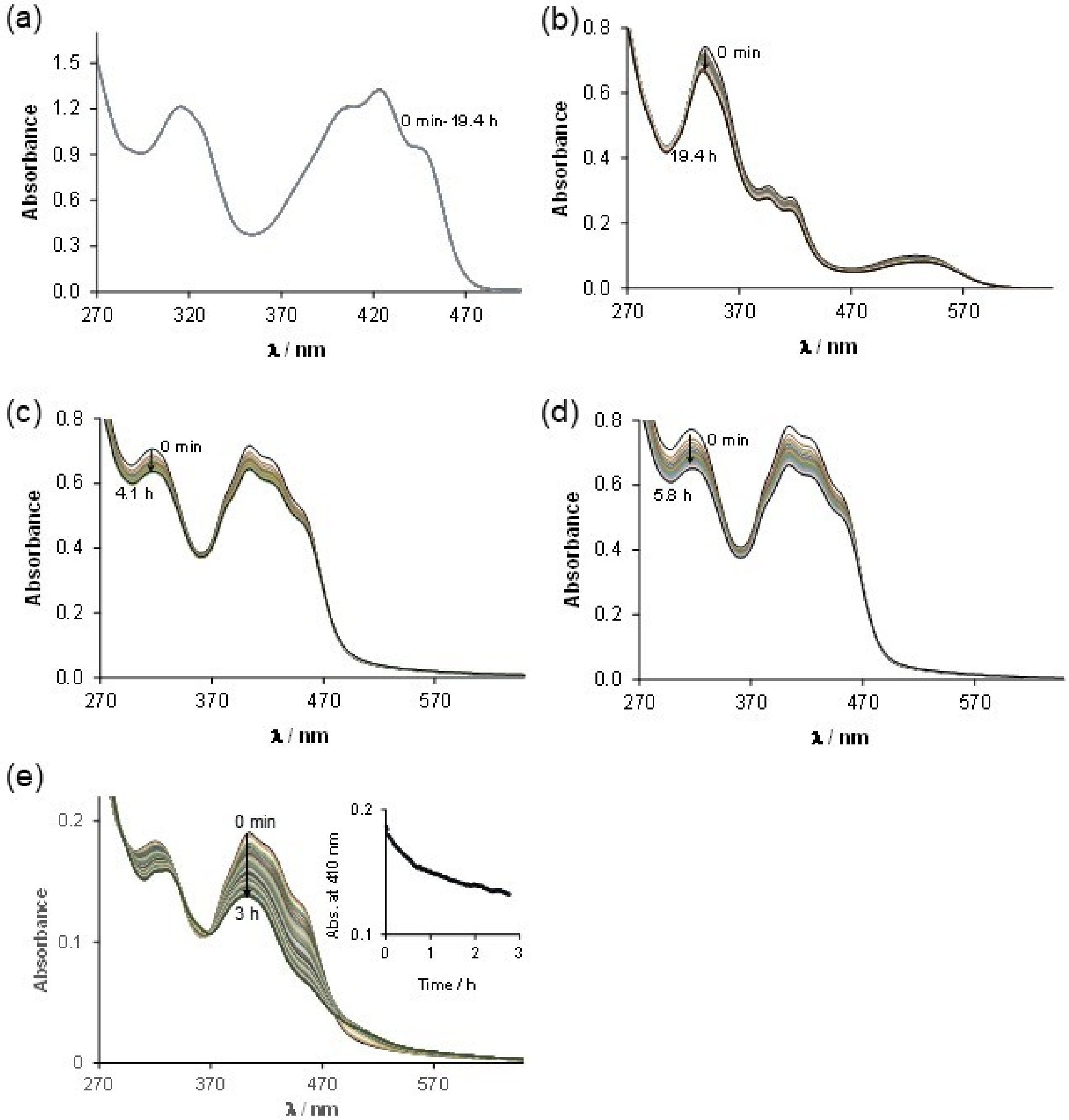

Figure S25. UV−vis absorption spectra recorded over time for $\mathbf{HL^{TMS}}$ in (a) DMSO, at pH (b) 2.0, (c) 7.4 (d) 11.7 and (e) at pH 7.4 under constant broad-band irradiation with a diode array photometer (D_2 + W lamp); {c = 50 µM (a-d) 12 µM (e); ℓ = 1 cm; T = 25.0 °C}.

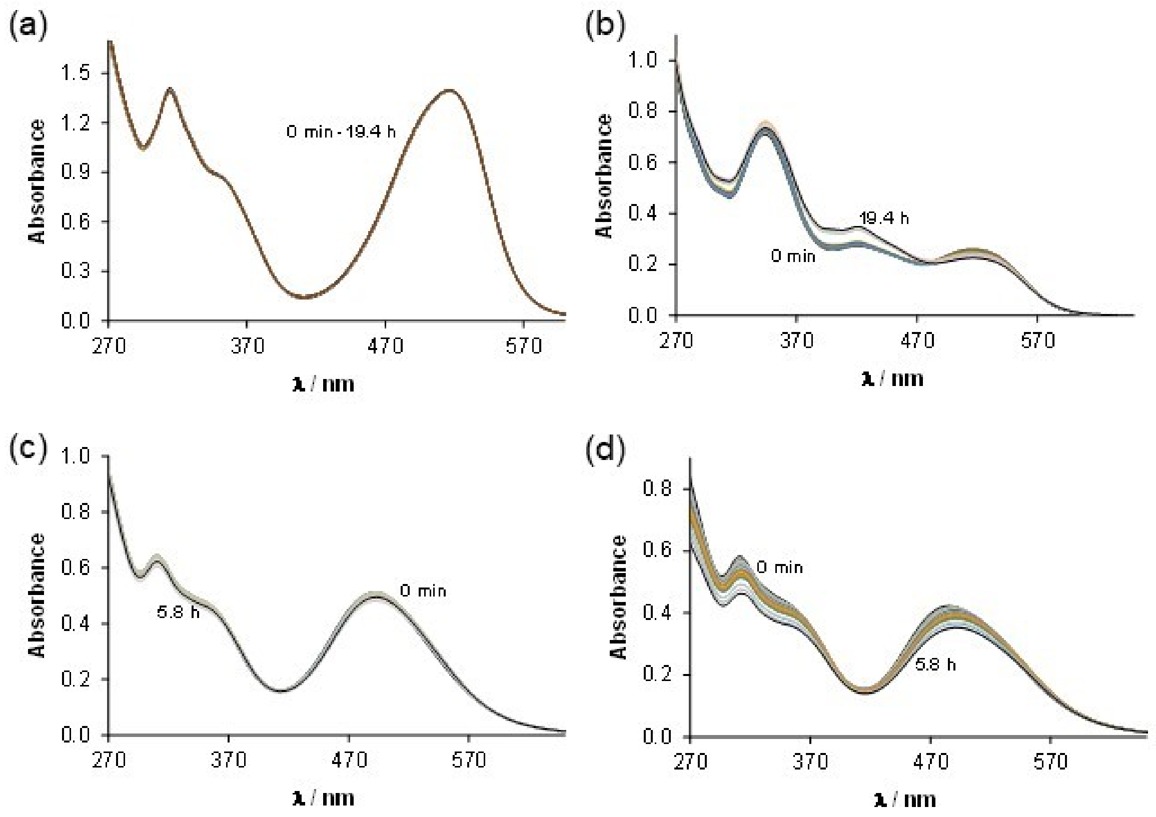

Figure S26. UV–vis absorption spectra recorded for **1** in a) DMSO, at pH b) 2.0, c) 7.4 and d) 11.7 over time; {c = 50 μM; ℓ = 1 cm; T = 25.0 °C}.

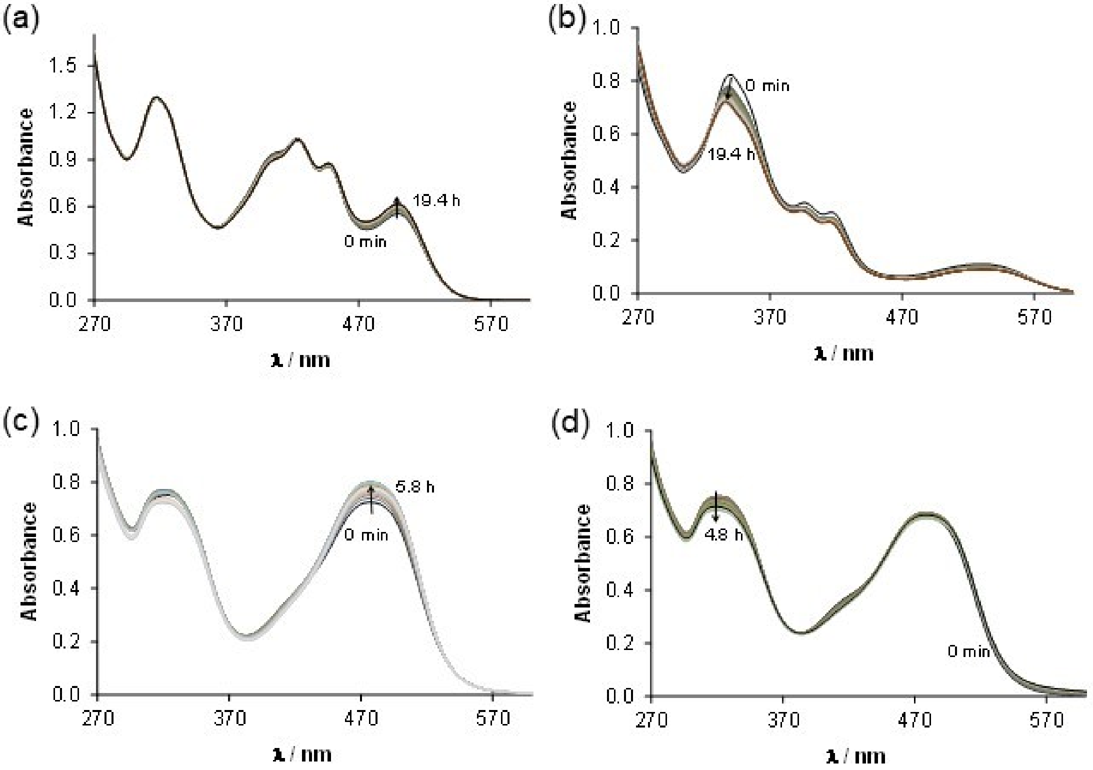

Figure S27. UV–vis absorption spectra recorded for **2** in (a) DMSO, at pH (b) 2.0, (c) 7.4 and (d) 11.7 over time; {c = 50 µM; ℓ = 1 cm; T = 25.0 °C}.

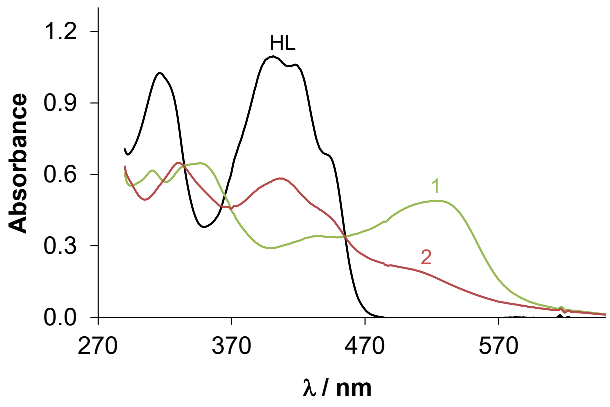

Figure S28. UV–vis absorption spectra recorded for the ligand $\mathbf{HL^{TMS}}$ and its complexes after dissolution in tetrahydrofuran; {c = 50 µM; ℓ = 1 cm; T = 25.0 °C}.

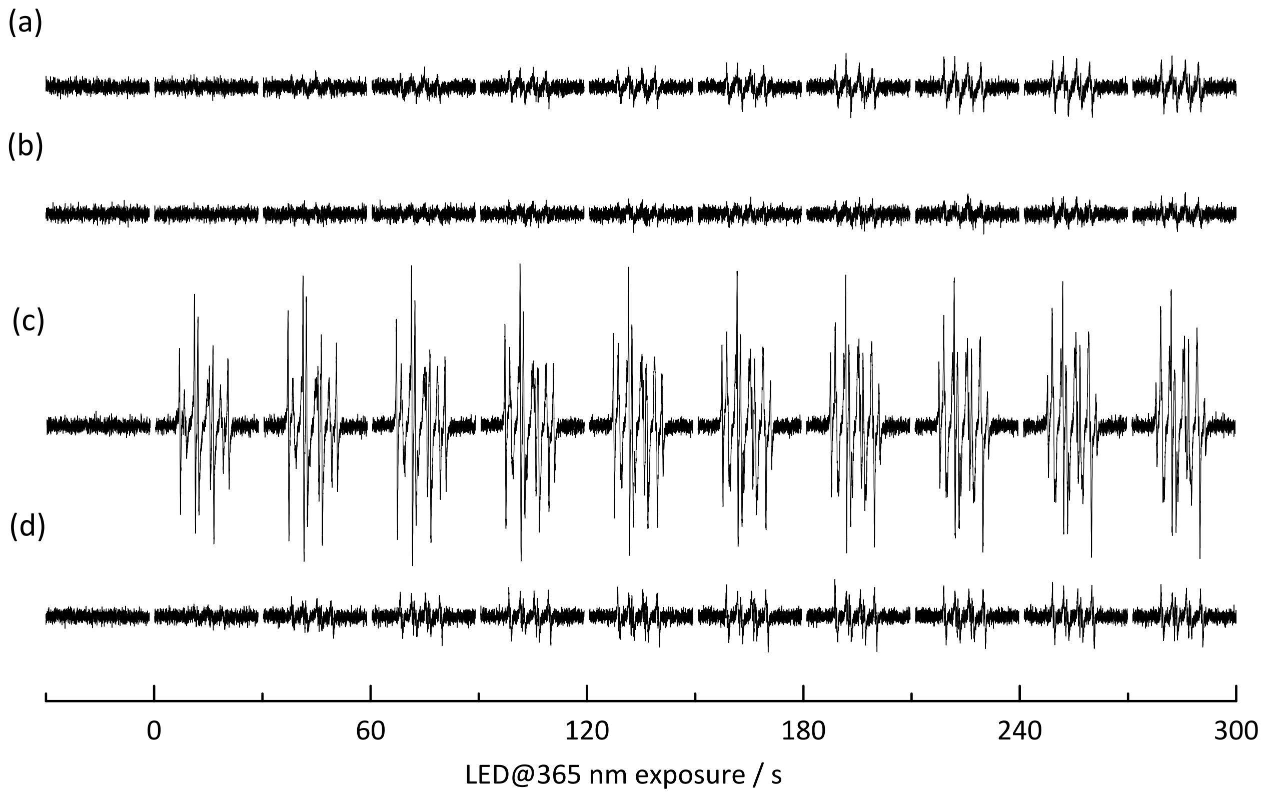

Figure S29. The time-courses of EPR spectra (SW = 10 mT) monitored upon LED@365 nm exposure (irradiance 18 mW cm^{-2}) of aerated (a) **HL^{TMS}**, (b) **1**, (c) **2** in the DMSO and (d) **2** in the 50% (*v/v*) DMSO:H_2O solutions containing spin trap DMPO. Initial concentrations: *c*(sample) = 33 μM and *c*(DMPO) = 35 mM.

NCI-60 One-Dose Screening

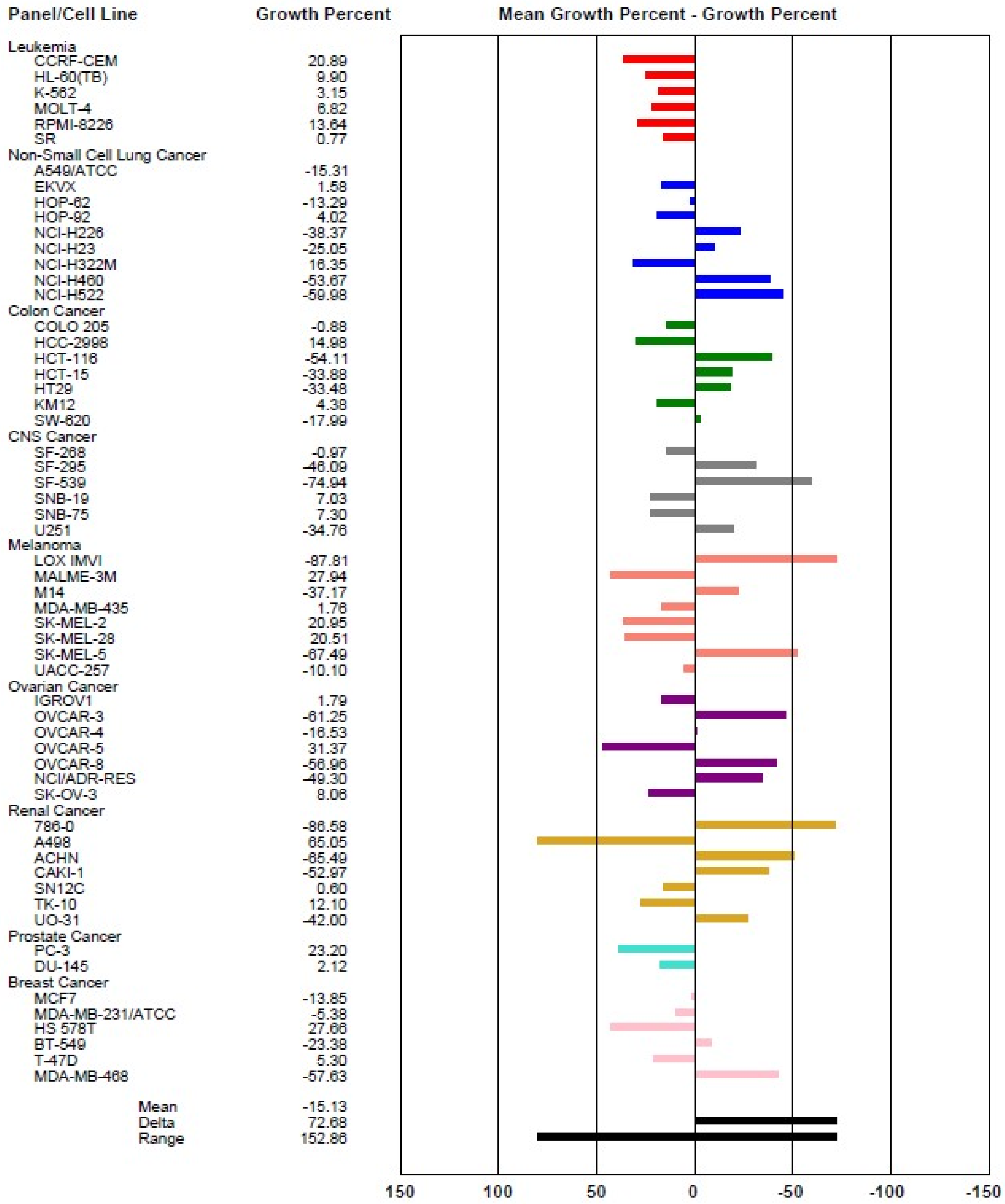

Figure S30. One-dose mean graph for **HL^{TMS}**.

Panel/Cell Line	Growth Percent
**Leukemia**	
CCRF-CEM	11.45
HL-60(TB)	20.29
K-562	2.87
MOLT-4	3.72
RPMI-8226	7.38
SR	4.20
**Non-Small Cell Lung Cancer**	
A549/ATCC	-88.32
EKVX	-90.58
HOP-62	-64.37
HOP-92	-73.56
NCI-H226	-89.82
NCI-H23	-92.99
NCI-H322M	-89.31
NCI-H460	-49.07
NCI-H522	-88.23
**Colon Cancer**	
COLO 205	-68.68
HCC-2998	-94.90
HCT-116	-78.77
HCT-15	-88.91
HT29	-83.97
KM12	-94.26
SW-620	-64.95
**CNS Cancer**	
SF-268	-80.64
SF-295	-92.42
SF-539	-93.66
SNB-19	-98.08
SNB-75	-75.86
U251	-91.52
**Melanoma**	
LOX IMVI	-90.91
MALME-3M	-92.56
M14	-93.48
MDA-MB-435	-87.68
SK-MEL-2	-96.02
SK-MEL-28	-96.61
SK-MEL-5	-97.49
UACC-257	-96.59
**Ovarian Cancer**	
IGROV1	-38.92
OVCAR-3	-94.31
OVCAR-4	-92.67
OVCAR-5	-96.29
OVCAR-8	-86.73
NCI/ADR-RES	-51.73
SK-OV-3	-91.87
**Renal Cancer**	
786-0	-89.55
A498	-97.87
ACHN	-97.55
CAKI-1	-97.62
RXF 393	-88.57
SN12C	-90.23
TK-10	-95.45
UO-31	-97.51
**Prostate Cancer**	
PC-3	-78.30
DU-145	-96.27
**Breast Cancer**	
MCF7	-91.69
MDA-MB-231/ATCC	-80.73
HS 578T	11.42
BT-549	-92.88
T-47D	-62.30
MDA-MB-468	-83.96
Mean	-74.85
Delta	23.23
Range	118.37

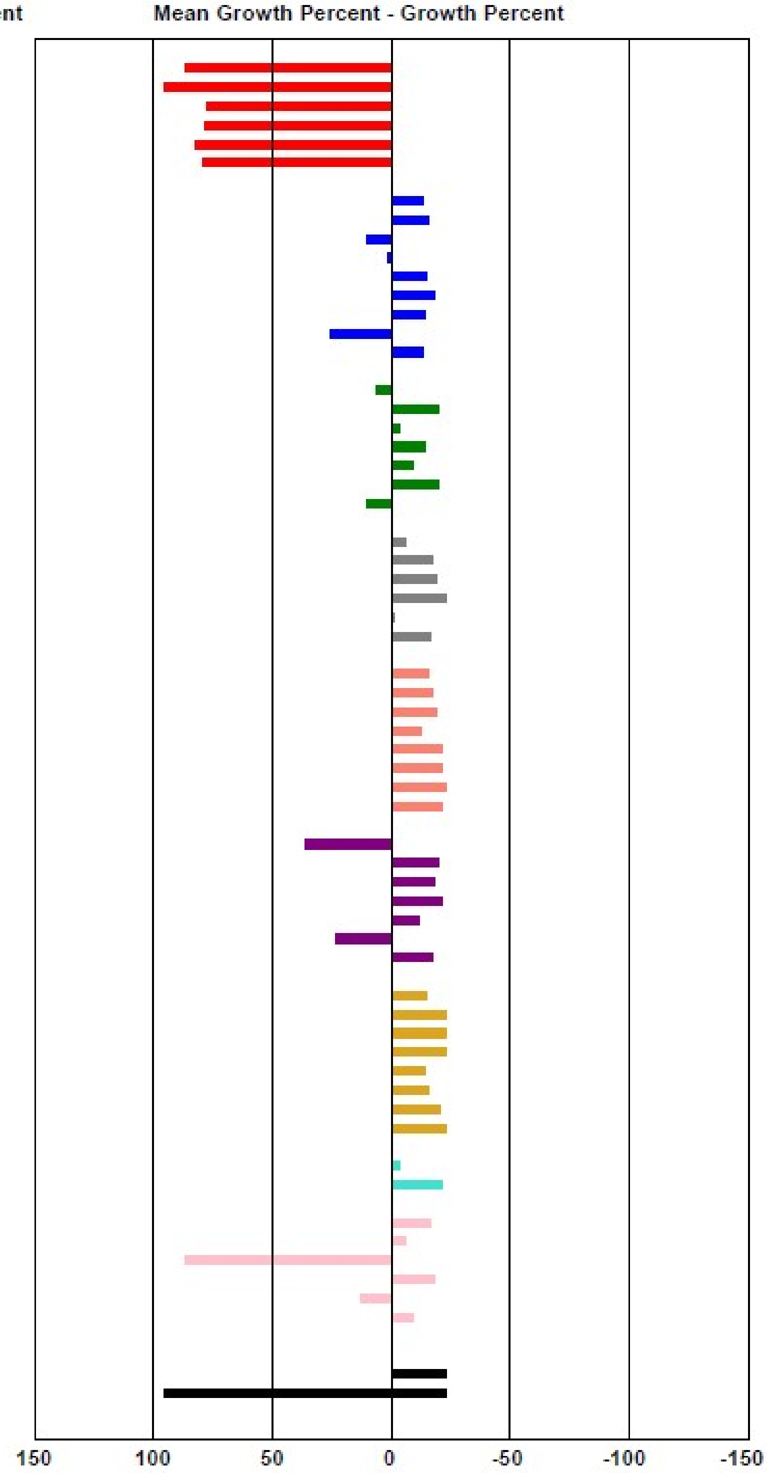

Figure S31. One-dose mean graph for **1.**

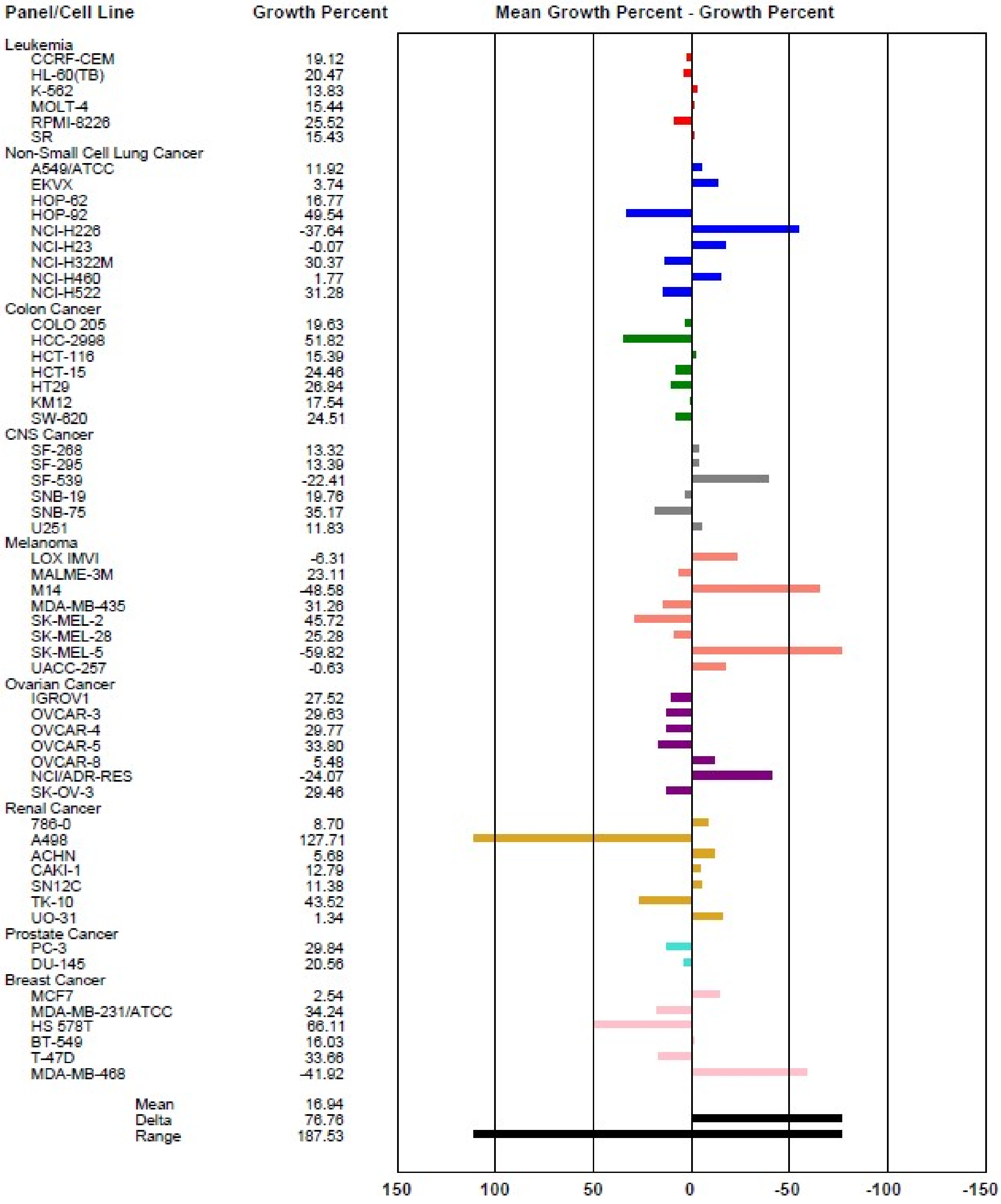

Figure S32. One-dose mean graph for **2**.

NCI-60 Five-Dose Screening

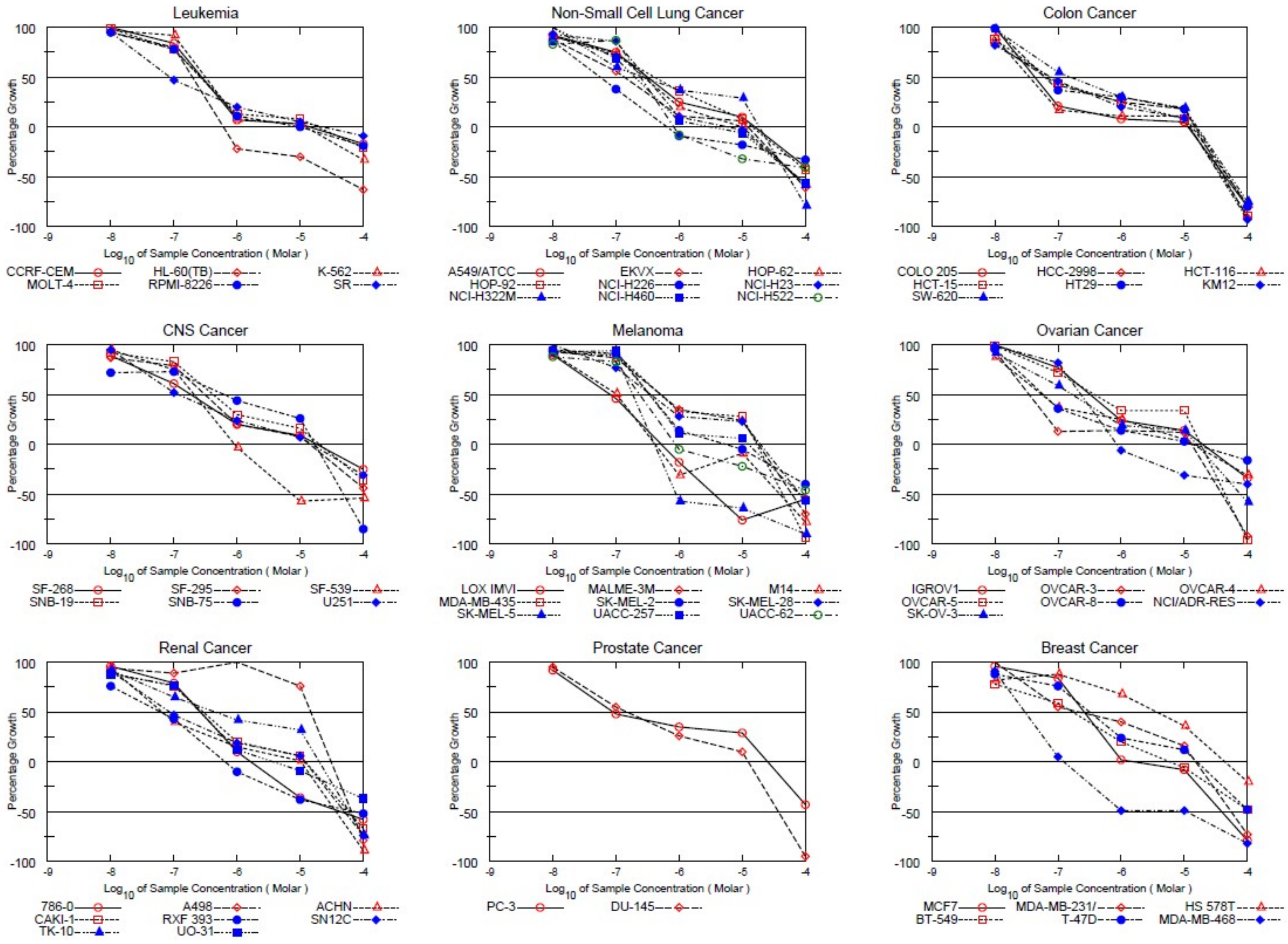

Figure S33. Dose response curves for **HL^{TMS}**.

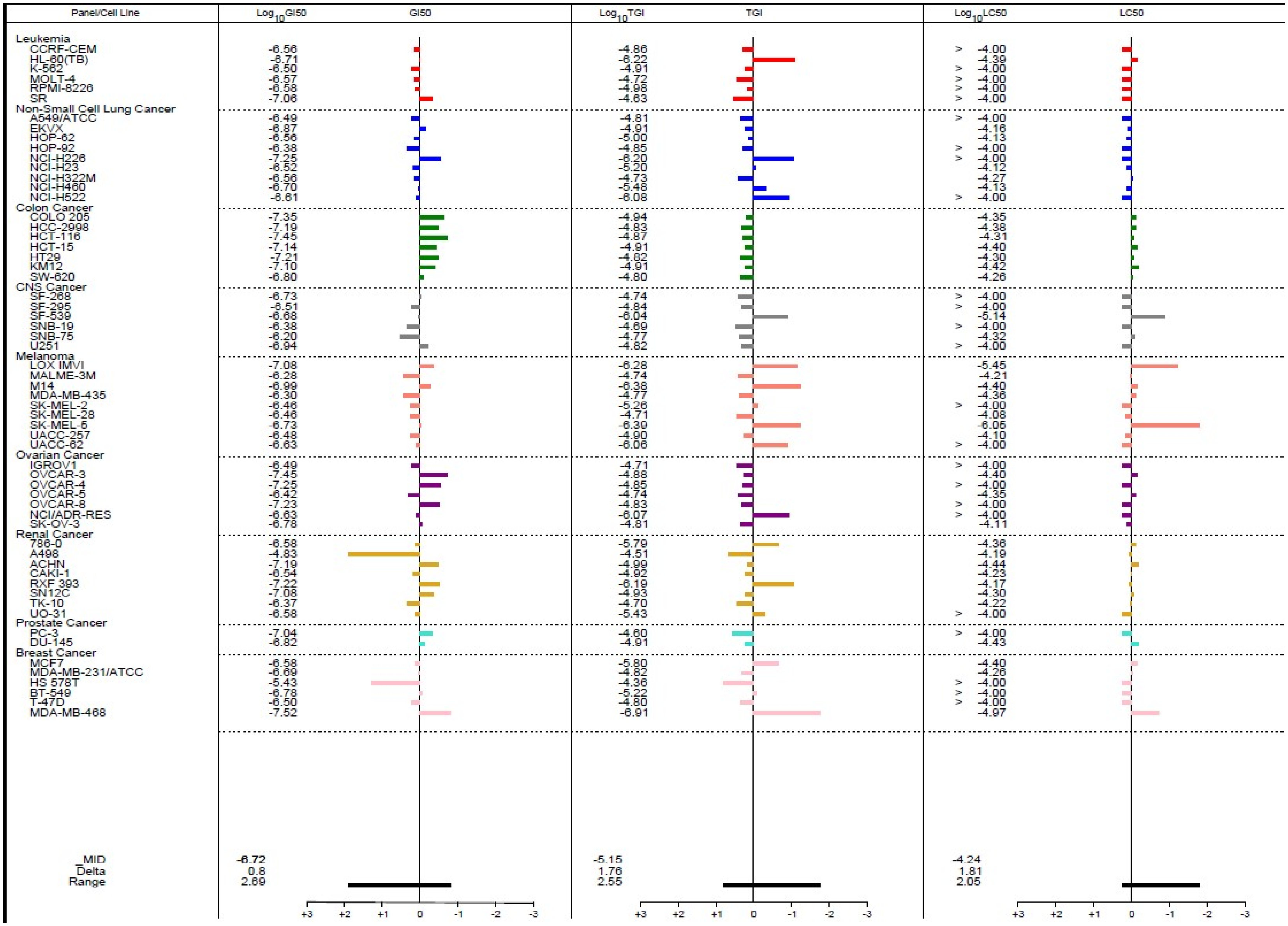

Figure S34. Five-dose mean graph for **HL^{TMS}**.

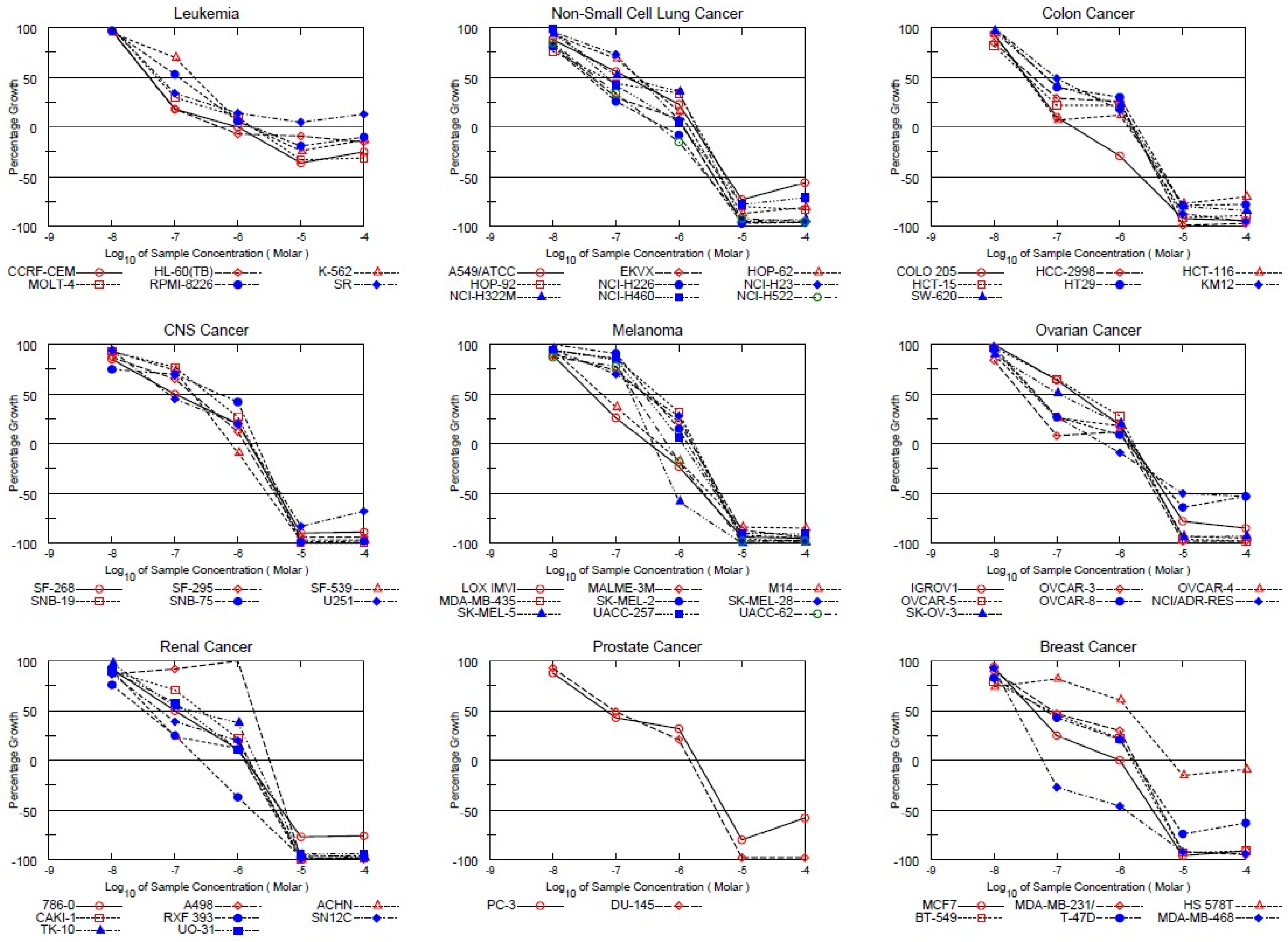

Figure S35. Dose response curves for **1**.

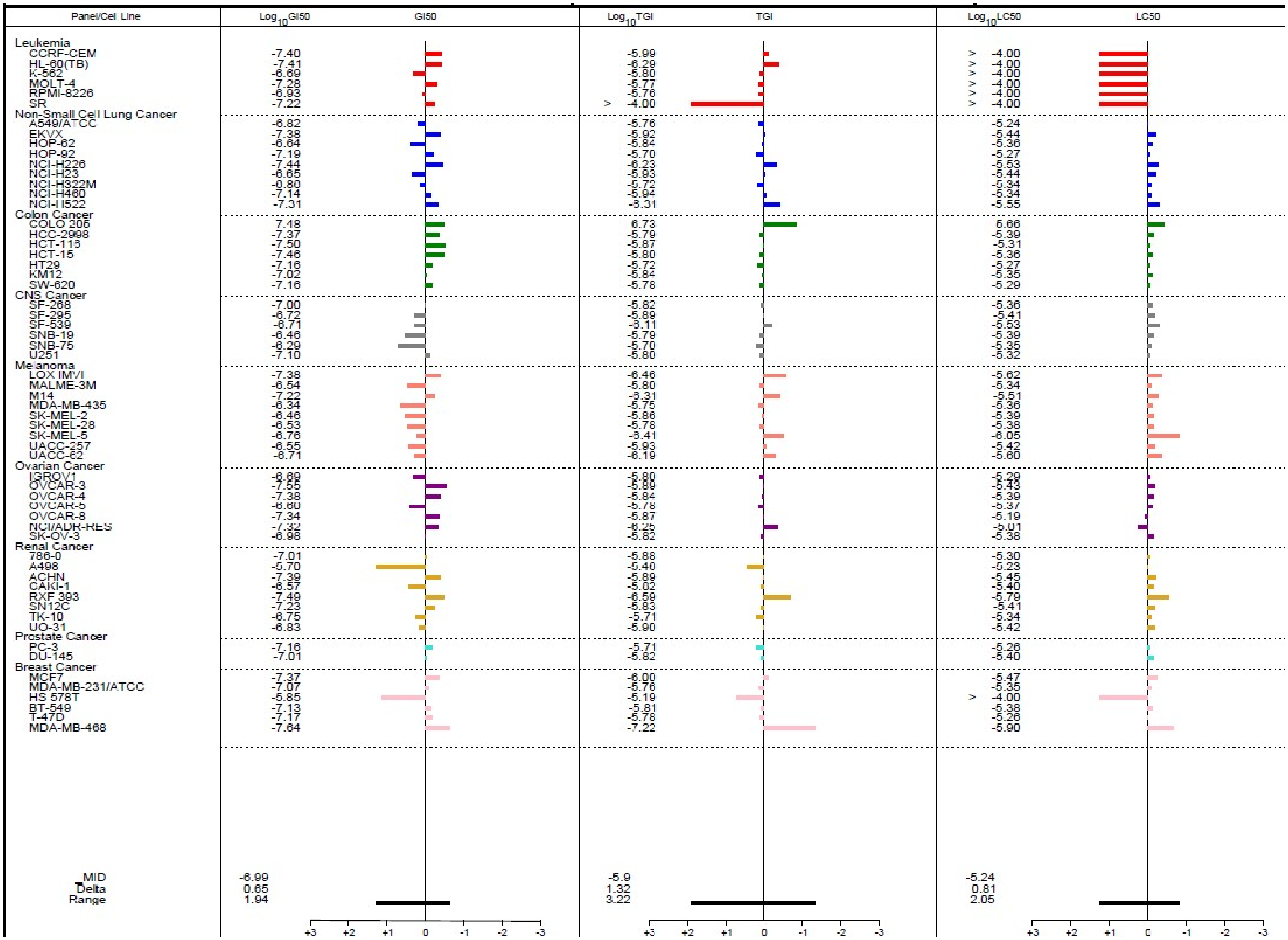

Figure S36. Five-dose mean graph for **1**.

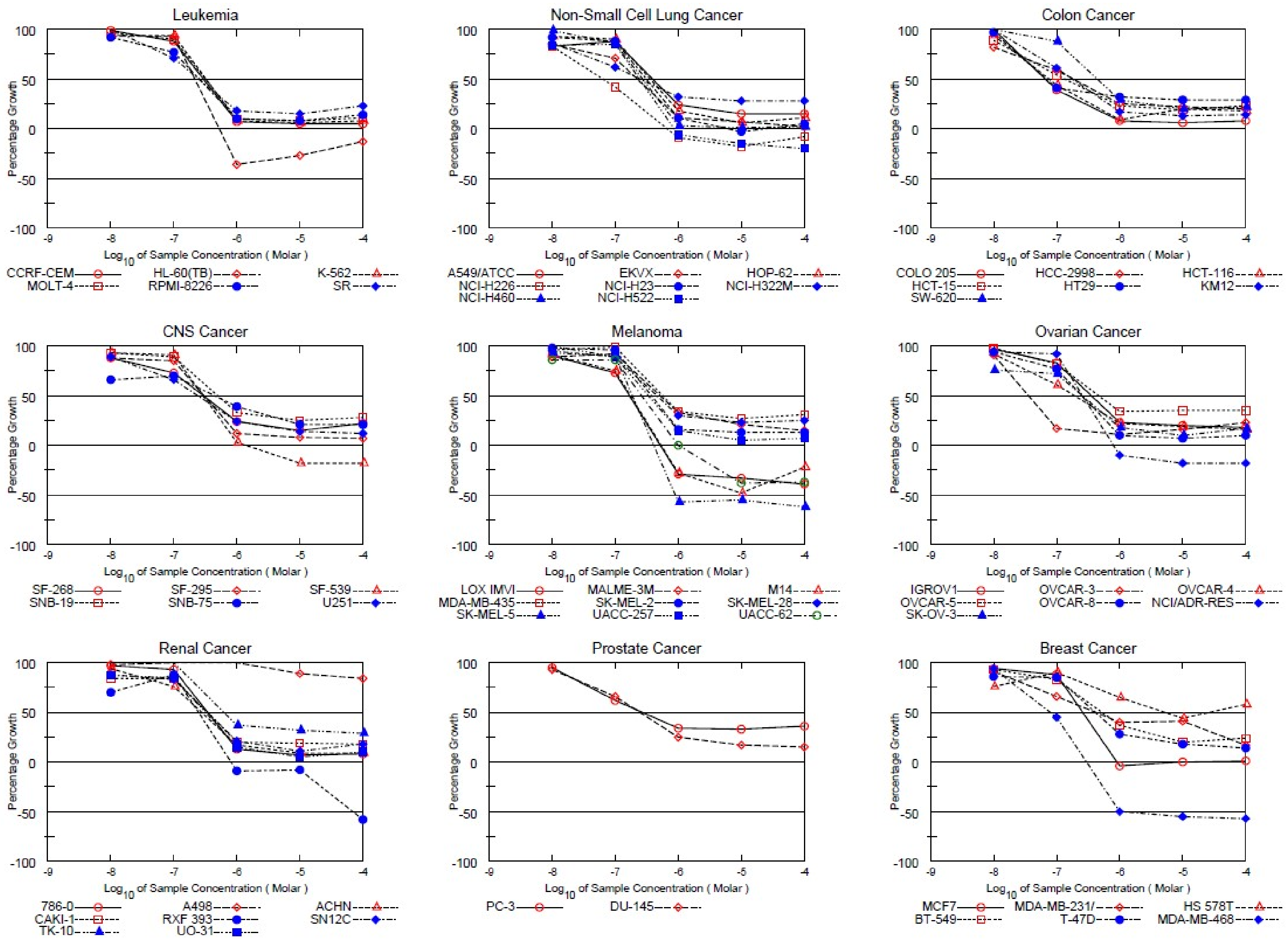

Figure S37. Dose response curves for **2**.

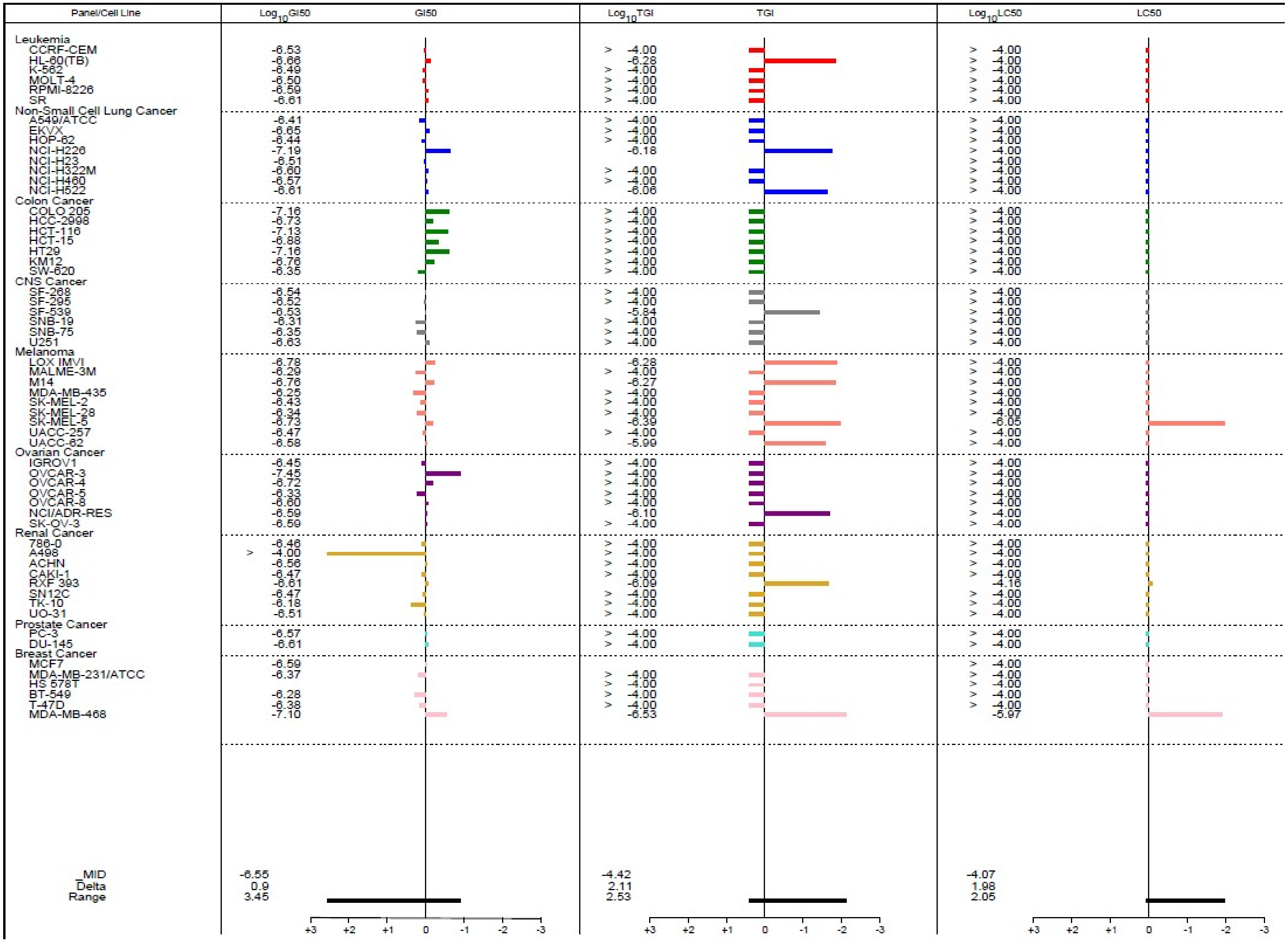

Figure S38. Five-dose mean graph for **2**.
